# Integrated genome-wide analysis of expression quantitative trait loci aids interpretation of genomic association studies

**DOI:** 10.1186/s13059-016-1142-6

**Published:** 2017-01-25

**Authors:** Roby Joehanes, Xiaoling Zhang, Tianxiao Huan, Chen Yao, Sai-xia Ying, Quang Tri Nguyen, Cumhur Yusuf Demirkale, Michael L. Feolo, Nataliya R. Sharopova, Anne Sturcke, Alejandro A. Schäffer, Nancy Heard-Costa, Han Chen, Po-ching Liu, Richard Wang, Kimberly A. Woodhouse, Kahraman Tanriverdi, Jane E. Freedman, Nalini Raghavachari, Josée Dupuis, Andrew D. Johnson, Christopher J. O’Donnell, Daniel Levy, Peter J. Munson

**Affiliations:** 1 The Framingham Heart Study and the Division of Intramural Research, National Heart, Lung and Blood Institute, National Institutes of Health, 73 Mt. Wayte Avenue, Suite 2, Framingham, MA 01702 USA; 2 Mathematical and Statistical Computing Laboratory, Center for Information Technology, National Institutes of Health, Bethesda, MD USA; 3 Institute for Aging Research, Hebrew SeniorLife, Harvard Medical School, Boston, MA USA; 4 National Center for Biotechnology Information, National Library of Medicine, National Institutes of Health, Bethesda, MD USA; 5 Department of Neurology, Boston University School of Medicine, Boston, MA USA; 6 School of Public Health, Harvard University, Boston, MA USA; 7 DNA Sequencing and Genomics Core, National Institutes of Health, Bethesda, MD USA; 8 Department of Medicine, University of Massachusetts Medical School, Worchester, MA USA; 9 National Institute on Aging, National Institutes of Health, Bethesda, MD USA; 10 Department of Biostatistics, School of Public Health, Boston University, Boston, MA USA; 11 Cardiology Section, Department of Medicine, Boston VA Healthcare, Boston, MA USA; 12 Section of Biomedical Genetics, Department of Medicine, Boston University School of Medicine, Boston, MA USA; 13 National Institutes of Health, Bldg 12A, Room 2003, Bethesda, MD 20892-5626 USA

## Abstract

**Background:**

Identification of single nucleotide polymorphisms (SNPs) associated with gene expression levels, known as expression quantitative trait loci (eQTLs), may improve understanding of the functional role of phenotype-associated SNPs in genome-wide association studies (GWAS). The small sample sizes of some previous eQTL studies have limited their statistical power. We conducted an eQTL investigation of microarray-based gene and exon expression levels in whole blood in a cohort of 5257 individuals, exceeding the single cohort size of previous studies by more than a factor of 2.

**Results:**

We detected over 19,000 independent lead *cis*-eQTLs and over 6000 independent lead *trans*-eQTLs, targeting over 10,000 gene targets (eGenes), with a false discovery rate (FDR) < 5%. Of previously published significant GWAS SNPs, 48% are identified to be significant eQTLs in our study. Some *trans*-eQTLs point toward novel mechanistic explanations for the association of the SNP with the GWAS-related phenotype. We also identify 59 distinct blocks or clusters of *trans*-eQTLs, each targeting the expression of sets of six to 229 distinct *trans*-eGenes. Ten of these sets of target genes are significantly enriched for microRNA targets (FDR < 5%). Many of these clusters are associated in GWAS with multiple phenotypes.

**Conclusions:**

These findings provide insights into the molecular regulatory patterns involved in human physiology and pathophysiology. We illustrate the value of our eQTL database in the context of a recent GWAS meta-analysis of coronary artery disease and provide a list of targeted eGenes for 21 of 58 GWAS loci.

**Electronic supplementary material:**

The online version of this article (doi:10.1186/s13059-016-1142-6) contains supplementary material, which is available to authorized users.

## Background

Implementation of high-resolution genotyping has led to a wave of genome-wide association studies (GWAS) of hundreds of phenotypes relevant to human health and disease [1]. Yet, the vast majority of the single nucleotide polymorphisms (SNPs) from GWAS that are associated with clinical traits and diseases reside in non-coding regions [2, 3]. This means that most disease-associated SNPs do not directly influence protein structure or function, but instead may act on phenotypes by affecting expression of local (*cis*) or distant (*trans*) gene targets (eGenes). Thus, characterizing the relations of DNA sequence to RNA expression is a critical step toward a better mechanistic understanding of disease, and ultimately toward improvements in diagnosis, prevention, and treatment. This endeavor begins with analysis of variation in messenger RNA (mRNA) expression levels associated with genotypic variation to identify expression quantitative trait loci (eQTLs) across the human genome [4].

The measurement of transcriptome-wide expression levels has facilitated several genome-wide eQTL studies [1, 4–8]. The sample sizes of some earlier eQTL studies, however, may have limited their statistical power [9], although a recent study [8] utilized a cohort of more than 2000 individuals and a previous study [5] used multiple cohorts totaling over 5000 individuals in meta-analysis. Of note, prior studies did not report results *trans-*eQTLs genome-wide. We report results of a microarray-based genome-wide eQTL study, considering both *cis* and *trans* elements, in whole blood samples from over 5000 participants in the Framingham Heart Study (FHS) [10, 11], a multi-generational community-based prospective study. To our knowledge, our study utilizes the largest, single-site study to date, and reports both gene-level and exon-level *cis*-eQTLs and *trans-*eQTLs genome wide.

## Results

Characteristics of the study sample [10, 11] are provided in Table 1. Participants in the FHS Third Generation cohort were about 20 years younger than those of the FHS Offspring cohort at the time of blood collection for RNA isolation. White blood cell counts and their proportions also differed between the cohorts.

**Table 1:** Demographic characteristics

Characteristic	Offspring cohort (*n* = 2240)	Generation 3 cohort (*n* = 3017)	*P* value*
Males (%)	45.1%	46.8%	0.2291
Age, in years	66.4 ± 9.0	46.4 ± 8.8	1.36E-895
White blood cell count (× 10^3^/mL)^b^	6.2 ± 1.3	6.0 ± 1.5	2.57E-7
Neutrophil (%)^b^	59.7 ± 7.9	58.7 ± 7.7	8.63E-7
Lymphocyte (%)^b^	27.1 ± 7.5	28.8 ± 6.9	2.20E-17
Monocyte (%)^b^	9.2 ± 1.9	8.6 ± 2.0	5.79E-22
Eosinophil (%)^b^	3.3 ± 1.6	3.1 ± 1.9	0.0039
Basophil (%)^b^	0.8 ± 0.2	0.8 ± 0.3	0.0019
Platelet count (× 10^3^/mL)^b^	253.0 ± 36.5	247.5 ± 51.5	6.34E-6

*P values are from two-sample t-tests. For sex phenotype, the *P* value is from Fisher’s exact test

^b^CBC values are imputed based on actual measurements of 2274 samples within the Generation 3 cohort

Out of 39 million imputed SNPs, we found 8.5 million with a minor allele frequency (MAF) ≥ 0.01 and imputation quality R^2^ ≥ 0.3 (See “Methods” for further details). Of these, we identified 2.2 million *cis-*eQTLs and 160 thousand *trans-*eQTLs at a nominal false discovery rate (FDR) < 0.05 (Table 2). We observed no inflation of the genomic control factor [12] (λ = 0.986). The quantile-quantile plot can be found in Additional file 1: Figure S1. We determined that polymorphism-in-probe effects [13], which occur when the variable position of a polymorphism overlaps an expression probe (Additional file 1: Figure S2), were generally minor, possibly affecting up to about 9.5% of the detected eGenes (see Additional file 1: Supplementary Methods for details). Moreover, these potential artifacts generally could be recognized by inspection of the individual exon-level results corresponding to that gene. Only one of the top 25 *cis-*eQTL-transcript cluster pairs (*C9orf78*, Table 4) was flagged for this artifact and that pair was not replicated in external datasets.

**Table 2:** Number of independent, significant eQTL-gene pairs with number of unique eQTLs or unique genes with *P* value corresponding to indicated FDR cutoff

Pair type	eQTL-TranscriptCluster pairs	Unique eQTLs	Independent pairs	Independent unique lead eQTLs	Unique genes^a^(% available)	*P* value cutoff
Nominal FDR < 0.05						
Cis	4,285,456	2,221,013	19,613	19,239	10,327 (58%)	1.00 E-4*
Trans	216,169	91,559	6741	5749	4958 (28%)	1.41 E-7
Nominal FDR < 0.0005						
Cis	3,698,429	2,008,734	17,452	17,119	9232 (52%)	1.78E-5
Trans	116,960	52,426	1464	888	1025 (6%)	8.82E-10
Available	1.521 E11	8,510,936	1.521 E11	8,510,936	17,873 (100%)	

*P value cutoff corresponding to FDR. Upper bound *P* value for pairs retained in computation was 1E-4, therefore highest attained FDR for *cis-*eQTLs was 0.0024

^a^Transcript cluster ID is used as a proxy for genes. Only 244 genes were represented by more than one Transcript cluster IDs. Approximately 270 Transcript cluster IDs could not be assigned to an Entrez Gene entry

**Fig. 1:**
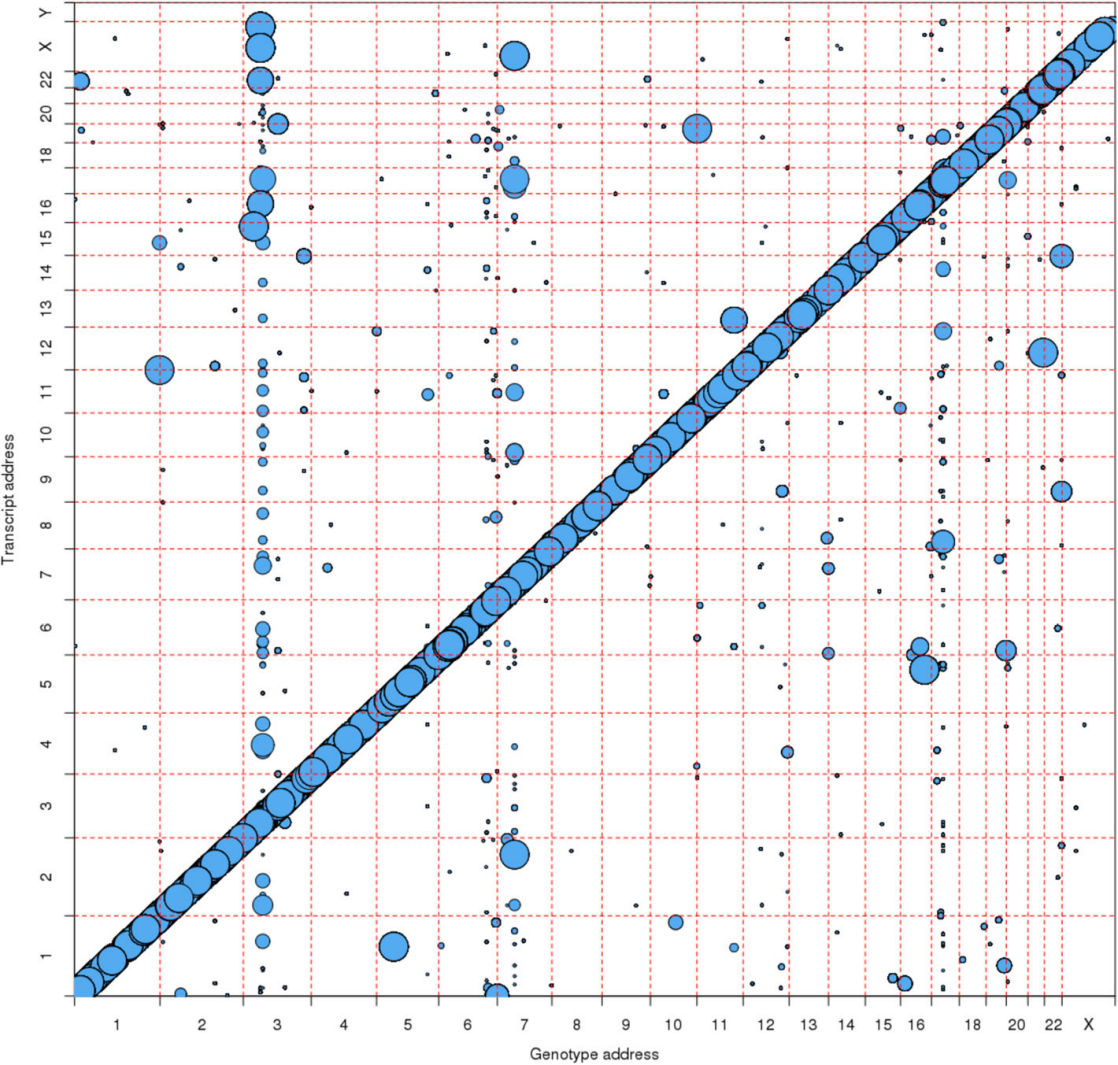
Genomic eQTL location vs. transcript cluster location for highly significant eQTL-gene pairs (FDR < 1E-8). Bubble size is inversely proportional to the FDR. The largest bubble indicates FDR < 1E-100

Recognizing that many of the significant eQTLs were in linkage disequilibrium (LD) with stronger, nearby eQTLs, we pruned our result using a stepwise linear regression procedure that identified a subset of the strongest, independent “lead-eQTLs” for each genetic region (see “Methods”). We found over 19,000 independent, lead *cis-*eQTLs and almost 6000 independent, lead *trans-*eQTLs, targeting over 10,000 *cis-* and almost 6000 thousand *trans-*eGenes. We found an eQTL for over half of the 17,873 measured transcript clusters (Table 2, Fig. 1, and Additional file 1: Figure S3). Use of a stricter nominal cutoff of FDR < 0.0005 reduced the number of independent *cis-*eQTLs and the number of targeted *cis-*eGenes by about 8% (Table 2). The stricter cutoff had a much larger effect on the number of *trans-*eGenes, reducing them by almost fivefold.

*Cis-*eQTLs are frequently defined as targeting expression of genes within 1 megabase (Mb) of the transcription start site (TSS). Others have noted that *cis-*eQTLs may be detected beyond the 1 Mb threshold [8]. We modified our definition of *cis-*eQTLs to include all eQTLs falling in an uninterrupted block around the TSS, provided there are no included gaps greater than 1 Mb in size. *Trans-*eQTLs were defined as those that target genes on other chromosomes or genes outside the contiguous *cis-* blocks (see “Methods” for details). We found long-range *cis-*eQTL blocks up to 10 Mb in width, e.g. for gene *BTN3A2* on chromosome 6. Such long-range *cis-*eQTLs were found for 255 transcript clusters, 75 of which, including *BTN3A2*, were located in the HLA region of chromosome 6; 22 were identified on chromosome X including gene *ITM2A* (8.7 Mb width), and 18 were found on chromosome 3 including gene UBA7 (7 Mb width). While some blocks may result from extended LD structure in the genome, others may point to extended patterns of regulatory sites. Our results support the conclusions of Kirsten et al. [8] who observed *cis-* associations extending to up to 5 Mb. In each contiguous region of eQTLs, we defined the “lead” eQTL as that which displayed the strongest association with its target transcript cluster, as defined by *P* value. The lead eQTL is the most likely causal eQTL, and for *cis-*eQTLs, its position relative to the TSS could be readily studied. For some eQTL blocks, we found that not all significant eQTLs were in LD with the primary lead eQTL but that secondary, independent lead eQTLs also could be found after accounting for the primary lead eQTL. Stepwise regression, including primary and successive independent lead eQTLs, determined a set of mutually independent lead eQTLs for each block (see “Methods” for details).

### Benefits of a large cohort

Use of a very large cohort size for eQTL analysis provided obvious benefits in terms of greater statistical power for discovery. To better quantify the value of cohort size, we considered whether the number of eGenes detected in our study would be detected with a smaller cohort. We repeated the full analysis using only the FHS Offspring cohort subset (*n* = 2240) and separately, only the FHS Third Generation cohort subset (*n* = 3017). Overall (Additional file 1: Table S1), we found that as the sample size dropped by roughly half, the number of unique *cis-*eGenes fell roughly proportionately, while the number of *trans-*eGenes declined to a much greater degree. Conversely, we concluded that our large sample size allowed for detection of many novel *cis-* and *trans-*eGenes. We found that our full cohort allowed detection of roughly 60% more *cis-*eGenes than did either smaller cohort. The full cohort detected three times to five times more *trans-*eGenes than did the smaller cohorts. It is clear that even with the current large cohort size, we have not yet detected all *cis-*eQTLs. We also found that the number of lead eQTLs (primary and secondary) per detected eGene increased using the full cohort (Additional file 1: Table S1). This demonstrates the power of the larger cohort to detect possible multiple SNPs on the pathways affecting expression.

As an example of the biological relevance of increasing the number of detected eGenes, consider the SNP rs1354034, a very strong GWAS hit for platelet count and platelet volume [14]. Using the full cohort, we detected 136 *trans-*eGenes that are targeted by variation at this locus. At least 27 of these genes are indeed known to be platelet-specific [15]. Analysis restricted to the smaller FHS Offspring cohort alone detected only 30 transcript clusters, 11 of which are platelet specific. Thus, increasing the sample size to include both FHS cohorts more than doubled the list of platelet-related genes. Further, when we consider the overlap of detected eQTLs with the GWAS catalog (see “Clinical relevance,” below), we found that restricting the analysis to the smaller cohort reduced the overlap by 33%. Thus, the full, large cohort clearly has greater power to annotate clinically relevant SNPs.

### Replication and validation

We assessed our results by three methods: (1) internal validation; (2) replication of previously published results (replication rate); and (3) the proportion of our results seen in earlier published studies (validation rate). Splitting our large sample into two roughly equally sized cohorts demonstrated an internal replication rate of 75% for *cis-*eQTL-transcript cluster pairs and 41% for *trans-*eQTL-transcript cluster pairs at the gene level, with 100% of the replicated pairs showing the same direction of change in expression (Additional file 1: Table S2).

We were able to replicate high proportions of eQTLs published in two previous eQTL studies even though they used different expression platforms. We replicated 69% of eligible *cis-*eQTL and 62% of *trans-*eQTL-transcript cluster pairs reported by Westra et al. [5] and 66% of *cis-*eQTL and 29% of *trans-*eQTL-transcript cluster pairs reported by Liang et al. [6]. We were able to replicate 59% of *cis-* and 56% of *trans-* results from a more recent study that used RNA-sequencing (RNAseq) methodology to report lead eQTLs [7]. These rates are 13 and 300,000 times the expected rates, for *cis-* and *trans-*eQTLs, respectively. The *P* values for these rates are <1E-200. We were able to replicate 36% of eligible *cis-*eQTL-transcript cluster pairs and 5.2% of *trans-*eQTL pairs from the largest, homogenous eQTL study available to date [8]. The replication rates are 78 and 30,000 times the expected rates, for *cis-*eQTLs and *trans-*eQTLs, respectively. The replication rates for the latter study might have been attenuated because of differences in RNA source (peripheral blood mononuclear cells versus whole blood) and use of different expression platforms (Illumina HT array versus the Affymetrix Exon array).

We explored external validation of our independent eQTL-transcript cluster pairs in two published studies and in seven datasets across multiple tissues in the NCBI Molecular QTL Repository [2, 4–6] (referred to as “Multiple studies” in Table 3) and in two more recent studies [7, 8] (see “Methods” for details). As expected, the *cis* external validation rates (Table 3) were lower than our internal validation rates. For Multiple Studies, we validated 54% of eligible lead *cis-*eQTL-transcript cluster pairs from our study, but only validated 2% of lead *trans-*eQTL results. The direction of effect matched in 89% of validated pairs. The RNAseq-based eQTL study of Battle et al. [7] reported only the lead variant for each targeted transcript. Since we did not expect perfect alignment with our lead eQTLs, we relaxed our matching criteria to count situations where our lead eQTL was in strong LD (R^2^ > 0.8) with their lead variant. Using this approach, we achieved external validation for 25% of our lead *cis-*eQTL-eGenes pairs but only for 4% of our lead *trans* pairs. When comparing our results with those of Kirsten et al. [8] using the same approach, we validated 58% of our eligible, independent lead *cis-*eQTLs and 6% of our *trans-*eQTLs. We observed that 85% of lead cis-eQTLs and 93% of lead trans-eQTLs validated by Kirsten et al. [8] also showed the same direction of effect as did our study. The detection rate and validated detection rate is dependent on the number of available probesets for the transcript, rising to a plateau when more than about 20 probesets are available (Additional file 1: Figure S4). Imperfect validation rates reflect a combination of factors: the potentially novel discoveries in our dataset as a result of the larger homogeneous sample size, the use of multiple genotyping chips of lower density in the comparison studies, the lack of imputation in one other study, differences among populations, and difficulties in accurately comparing transcript expression levels measured with different platforms.

**Table 3:** Number of independent, significant pairs validated in previous studies

Pair type	Comparison study	Eligible lead eQTL-gene pairs^a^	Validated pairs^a^ (rate)	Expected pairs (rate)
Cis	Multiple studies [2, 4–6]	10,584	5700 (54%)	90 (0.8%)
	Battle et al. [7]	11,466	2911 (25%)	10 (0.08%)
	Kirsten et al. [8]	11,179	6503 (58%)	919 (8%)
Trans	Multiple studies [2, 4–6]	1777	40 (2%)	0.0007 (0%)
	Battle et al. [7]	2596	102 (4%)	0.0001 (0%)
	Kirsten et al. [8]	2337	135 (6%)	0.03 (0%)

^a^See “Methods” and Additional file 1: Supplementary Methods for details

*P* values (comparing Validated to Expected pairs are based on Poisson distribution) are all <1E-200

**Fig. 2:**
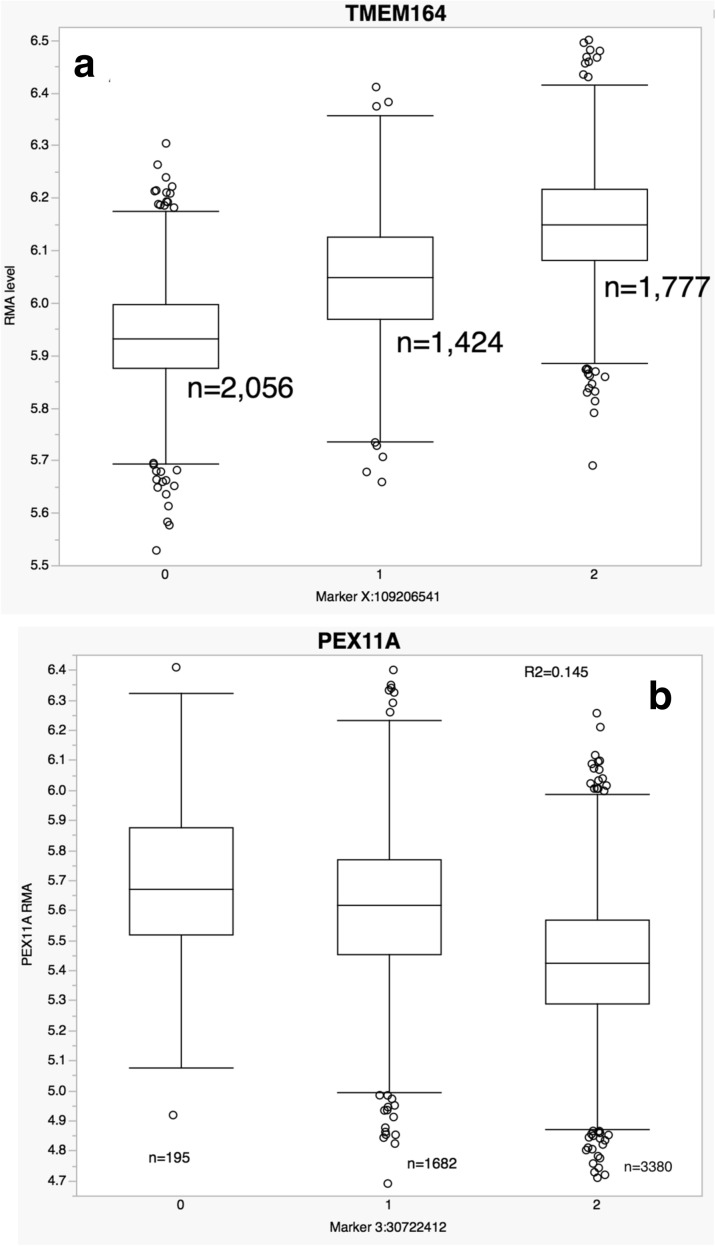
*Box plots* of very strong *cis-*eQTL or *trans-*eQTL-transcript cluster pairs. **a** rs2499412 – TMEM164 (cis, R^2^ = 47%). **b** rs3773654 – PEX11A (trans, R^2^ = 16%). *y-axis*: expression level in RMA units; *x-axis*: imputed major allele count

The top 25 lead *cis-*eQTL and *trans-*eQTL transcript cluster pairs, ranked by percent of variance explained (R^2^), are presented in Table 4. Illustrative box-plots for a *cis-*eQTL and a *trans-*eQTL are given in Fig. 2. For the top *cis-*eQTL gene pair (rs12231872 with *CLEC12A*) more than half of the variation in expression of the eGene was explained by the *cis-*eQTL. Likewise, the top *trans-*eQTL (rs6592965) explained over 22% of the variation in expression of the corresponding *trans-*eGene (*SLC38A5*). Interestingly, ten of the 25 (or 40%) top *cis-*eQTLs were in significant LD with or were themselves GWAS hits (at *P* < 5E-8), providing support for the idea that genetically determined effects on gene expression have phenotypic consequences. Perhaps more noteworthy is the finding that 17 of the 25 (68%) top *trans-*eQTLs were also either GWAS hits or in significant LD with GWAS hits. Again, this supports the notion that not just *cis-*eQTL but also *trans-*eQTL effects may explain the mechanism of action of these genetic variants. We found external validation for 18 of 25 (72%) top *cis-*eQTL-eGene pairs in at least one of four published datasets [5–8], a high rate that perhaps should be expected for such prominent associations. We found evidence of external validation for only six of 25 (24%) top *trans-*eQTL pairs, perhaps because few published studies have reported full genome-wide *trans-*eQTL results.

**Table 4:** Top 25 non-redundant gene level *cis*-eQTL and top 25 *trans*-eQTL-transcript cluster pairs

eQTL marker position	Rs ID	Transcript cluster ID	Trans Chr	Gene symbol	R^2^	Beta	Cluster number
Top *Cis-*eQTL pairs							
12:10118747	rs12231872	3404530 R	12	*CLEC12A*	57%	−0.15 [G]	
15:48596713	rs74011998 G	3593065	15	*SLC12A1*	56%	−0.15 [T]	
5:96252589	rs2910686 H G	2821347 R	5	*ERAP2*	55%	−0.11 [T]	
1:207280764	rs12063500 G	2377165 R	1	*C4BPA*	54%	−0.32 [C]	
6:31238135	rs1050317 G	2948887	6		50%	−0.3 [A]	
6:32576341	rs9271093 G	4048241 R	6	*HLA-DRB5*	50%	−0.45 [A]	
22:42498204	rs12157818	3947310 R	22	*C22orf32*	49%	0.16 [C]	
6:26354100	rs67509210 G	2899333 R	6	*BTN3A2*	48%	0.19 [G]	
7:150480007	rs1985881	3079172 R	7	*TMEM176B*	48%	−0.17 [C]	
4:6697822	rs3822260 H G	2717078	4	*S100P*	48%	0.24 [C]	
22:45744854	rs8136319	3948543 R	22	*FAM118A*	48%	−0.2 [G]	
6:167382449	rs434093 G	2984884 R	6	*RNASET2*	48%	0.12 [T]	21
1:17421764	rs2076613	2398820	1	*PADI2*	47%	0.08 [T]	
X:109206541	rs2499412	3987029	X	*TMEM164*	47%	0.11 [G]	
4:47858518:ATAG_		2768273	4	*NFXL1*	47%	−0.1 [R]	
5:64858687	rs432206 H	2859667 R	5	*CENPK*	47%	−0.14 [C]	
7:150478052	rs6464101 H	3031624 R	7	*TMEM176A*	47%	−0.15 [G]	
1:109706880	rs647294 H	2350489 R	1	*KIAA1324*	47%	0.14 [G]	
6:32575544	rs9271061 G	4048265 R	6	*HLA-DRB1*	46%	−0.55 [T]	
5:102118794	rs2431321 H	2822215 R	5	*PAM*	46%	−0.08 [T]	
6:32603854	rs9272302 G	2903219 R	6	*HLA-DRB6*	46%	−0.77 [C]	
7:26952139	rs2960785 H	3042610 R	7	*SKAP2*	46%	0.08 [C]	
1:43265985	rs2816599	2409069 R	1	*CCDC23*	45%	−0.31 [C]	
9:132588337	rs7470675	3227121 S	9	*C9orf78*	45%	0.17 [T]	
7:75247329	rs1186222	3057370 R	7	*HIP1*	45%	0.09 [C]	
Top *Trans-*eQTL Pairs							
7:50427982	rs6592965 G	4007437 R	X	*SLC38A5*	22%	0.09 [G]	25
17:44026739	rs242562 H G	3767230	17	*LRRC37A3*	20%	0.16 [G]	
3:30722412	rs3773654	3638566	15	*PEX11A*	16%	–0.18 [G]	
1:58992071	rs7520008	2414558	1	*DAB1*	12%	0.04 [T]	
3:50078541	rs9814664 H G	3981931	X	*ZCCHC13*	12%	0.11 [T]	9
7:50427982	rs6592965 G	2520069 R	2	*C2orf88*	11%	0.1 [G]	25
1:248039451	rs3811444 H G	3357237 R	11	*JAM3*	11%	0.08 [C]	4
3:49978069	rs6772095 H G	4024310	X	*SOX3*	11%	0.06 [C]	9
16:69973655	rs4985461	2876543	5	*TIFAB*	11%	–0.08 [G]	
7:50427982	rs6592965 G	3724505	17	*MYL4*	9%	0.08 [G]	25
21:44473062	rs11700748 H G	3416019	12	*PRR13*	9%	0.1 [C]	56
5:50106439	rs32396 H G	2359431	1	*LCE1F*	8%	0.08 [G]	
11:617537	rs2740380 G	3864107	19	*PSG7*	8%	0.13 [T]	
3:49971514	rs7613875 H G	3693591	16	*PRSS54*	8%	0.06 [C]	9
3:50008566	rs6446189 G	3939154	22	*RAB36*	8%	0.05 [A]	9
3:56849749	rs1354034 H G	3724545 R	17	*ITGB3*	8%	–0.08 [T]	10
11:108623805:CTAT_		3504617	13	*SKA3*	8%	–0.1 [R]	
17:33875262	rs8073060 H	3089102 R	8	*EPB49*	7%	–0.05 [T]	51
3:56849749	rs1354034 H G	2735759	4	*MMRN1*	7%	–0.07 [T]	10
11:55113534	rs75905900 G	3329983	11	*PTPRJ*	7%	–0.05 [A]	
6:170847101	rs75159687	2390976	1	*LINC00115*	7%	–0.13 [T]	
22:50027210	rs5769712	3581090	14	*TMEM179*	7%	0.06 [C]	58
7:50427982	rs6592965 G	3714729 R	17	*MAP2K3*	7%	–0.05 [G]	25
22:50027210	rs5769712	3203199	9	*TAF1L*	6%	0.07 [C]	58
3:56849749	rs1354034 H G	2476510	2	*LTBP1*	6%	–0.06 [T]	10

R^2^ - Percentage variance explained

Marker position is annotated as chromosome number:location in hg19 coordinate

Beta is regression estimate (log base 2 expression difference per dosage of effect allele), with effect allele in brackets. [R] refers to the reference allele for an indel polymorphism

H = HapMap SNPs

G = in LD with GWAS SNPs as recorded in the NHGRI GWAS catalog

R = In LD with SNP replicated in at least one of the databases associated with references GTEx [1], Westra et al. [5], Liang et al. [6], Kirsten et al. [8]

All 25 *cis-*eQLTs and 25 *trans-*eQLTs are internally validated and have consistent sign of expression change

S = SNP-in-probe problem likely inflates R^2^

The Affymetrix Exon Array provides expression measurements at the transcript cluster level, but also for individual exons within the transcript cluster. At the exon level (Additional file 1: Table S3), we detected many of the same *cis-*eQTLs for individual probesets of the same genes identified at the gene-level. The top exon-level *trans-*eQTLs also duplicated many of the results seen at the gene-level, including many *trans-*eQTLs found to be part of *trans-*eQTL blocks or clusters (discussed in detail below). However, the percentage of variance of expression levels of these exons explained by their *trans-*eQTLs is generally much higher than that for the corresponding gene-level results, probably because the gene-level analysis averages over multiple exons that demonstrate considerable variation.

### Enrichment of lead eQTL location relative to gene structure and neighborhood

A major goal of eQTL studies is to identify true gene transcription regulatory elements. Previous analyses [5, 7, 8] have shown a strong dependence of eQTL position relative to the TSS and transcription end sites (TES) of each gene. We analyzed 8475 protein-coding eGenes with identifiable gene structure and without suspicion of polymorphism-in-probe effects, to identify preferences for locations of all significant eQTLs and of the lead eQTL. We found that lead eQTLs are frequently (for 35% of eGenes) found in the transcribed region of the gene, a ninefold enrichment compared to elsewhere in the 2 Mb region centered on the TSS (Additional file 1: Table S4). The lead eQTL is also frequently (38%) in the upstream *cis-*intergenic region, but less often than expected. The lead eQTL is less frequently (28%) in the downstream *cis*-intergenic region (Additional file 1: Table S4). The distance from upstream lead eQTLs to the TSS follows a multi-exponential decay curve with a median distance of about 27 kb. The distance downstream from the TES to the lead eQTLs follows a similar multi-exponential distribution with a slightly longer median distance of about 31 kb. A graphical representation of the observed distribution of lead eQTL locations is given in Additional file 1: Figure S5. Within the transcribed region, exonic locations are highly enriched (25 fold) for lead eQTLs, more so than for intronic locations (12-fold). The first exon and the 5’- UTR are especially enriched for lead eQTLs (45-fold) while other exons, the 3’-UTR, the first intron and subsequent introns (21-fold, 20-fold, 11-fold, and 8-fold, respectively) show lesser degrees of enrichment (Additional file 1: Table S4). Thus, it is clear that lead *cis-*eQTLs act preferentially through regulatory elements within the first exon, within the 5’-UTR or near the TSS. We also analyzed just the secondary lead eQTLs which show independent significant associations with about half of the targeted transcript clusters. Again, the 5’-UTR again was maximally enriched (30-fold) in these lead eQTLs. The pattern of enrichment was nearly identical but somewhat weaker than that for the primary lead eQTLs, This shows that secondary lead eQTLs also convey important information regarding functional sites.

### Enrichment of lead eQTLs at regulator sites

To further explore the regulatory sites, we compared our results to RegulomeDB [16], a summary of evidence for a regulatory role for each SNP, based on DNAase hypersensitivity, transcription factor binding sites, and biochemically characterized regulatory promoter regions. Specifically, we tested whether our lead *cis-*eQTLs excluding those targeting polymorphism-in-probe transcripts) were enriched for regulatory roles (i.e. low RegulomeDB scores) compared to other *cis-*SNPs within 1 Mb of each transcript start site, having no such evidence. Results, summarized in Additional file 1: Table S5, show strong enrichment of regulatory evidence for all (primary and secondary) lead *cis-*eQTLs (sevenfold enrichment, *P* < 1E-89). The primary lead *cis-*eQTLs alone showed a stronger enrichment (eightfold, *P* < 1E-69), but with a minor attenuation in significance level. This result suggests that lead eQTLs are indeed identifying regulatory sites and that the primary lead eQTLs are the most likely regulatory position in a given neighborhood. Only a barely significant, twofold excess of lead *trans-*eQTLs were found with low RegulomeDB scores, suggesting that at most a modest fraction of *trans-*eQTLs are acting at known regulatory sites.

### Clusters of *trans*-eQTLs

**Fig. 3:**
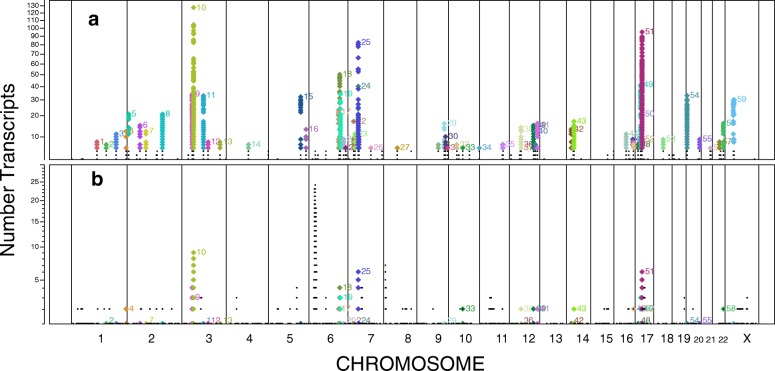
Number of transcript clusters targeted by each *trans-*eQTL. eQTLs targeting six or more extrachromosomal transcript clusters fall into color-coded clusters. **a** Number of extra-chromosomal *trans* targets. **b** Number of intrachromosomal *trans* targets. Note presence of unlabeled clusters on Chr 6, the HLA region, and on Chr 8

Some *trans-*eQTLs are associated with multiple distant transcripts and can be grouped into compact genomic blocks or clusters (see “Methods”). Although such “regulatory hotspots” can arise from confounding factors such as batch effects [17], we used methods that reduce or avoid such spurious associations (see “Methods”). At the gene level, we identified 59 distinct clusters of *trans-*eQTLs, each targeting a set of six to 141 distant transcripts (Table 5, Fig. 3). Studying the targets of these clusters may illuminate the functional roles of these eQTLs. For example, such *trans-*eQTL clusters may be a result of downstream consequences of a variant within a haplotype block [18]. The most prominent *trans-*eQTL clusters are on chromosomes 3 and 17 (Clusters 10, 51, and 52) and are associated with expression of platelet-specific genes, such as *CTTN*, *HIST1H3H*, and *MMD* [15, 19, 20]. SNPs in these clusters were reported to be associated in GWAS [4] with platelet count and mean platelet volume (e.g. rs1354034 and rs12485738 on chromosome 3; rs10512472 and rs16971217 on chromosome 17) [21]. Variation in platelet count or volume would likely cause changes in the proportion of RNA derived from platelets in the whole blood sample and thus, variation in the apparent expression levels of platelet associated genes. We found 13 platelet-related GWAS clusters (Table 5, Additional file 1: Table S6), many of which also had target gene sets enriched with platelet-specific genes. In addition, Cluster 1 may contain an undiscovered platelet-associated variant, as it is associated with enrichment for platelet-related genes.

**Table 5:** Clusters of *trans-*eQTLs with many targets

Cluster	Cluster address (hg19)	Width	N	Ng	Tr	Tr-max	Tr-Ext	Ret	GWAS traits	Top GSEA categories
1	chr1:118158079-118174222	16,144	8	0	10	8	0	1		Platelet
2	chr1:158810312-158827365	17,054	2	0	7	7	0	0		
3	chr1:205040943-205268803	227,861	114	4	14	11	0	5	Mean platelet volume, Platelet counts	.
4	chr1:248038210-248047688	9479	4	3	15	12	2	1	Mean corpuscular volume, Platelet counts, Red blood cell traits	.
5	chr2:8740722-8766192	25,471	23	0	24	21	0	2		Catabolic process
6	chr2:60708597-60727629	19,033	19	24	18	15	7	1	Beta thalassemia/hemoglobin E disease, F-cell distribution, Fetal hemoglobin levels, Sickle cell anemia (hemolysis)	.
7	chr2:85715315-85809703	94,389	51	1	15	12	0	0	Prostate cancer	Neutrophil
8	chr2:160326049-160674656	348,608	381	0	26	21	1	0		Liver cancer progression, fibroblast cell response, Neutrophil, MicroRNA targets
9	chr3:49734040-50308718	574,679	435	2	38	34	0	0	HDL cholesterol, Menarche (age at onset)	Dendritic cells response
10	chr3:56829892-56953738	123,847	185	9	128	127	85	3	Mean platelet volume, Platelet counts	Platelet, hemostasis, Neutrophil
11	chr3:100905910-101550022	644,113	544	0	37	33	17	0		Genes in band chr19q13, transcription pathway
12	chr3:121350573-121471367	120,795	35	0	8	8	0	1		
13	chr3:176869498-176928657	59,160	104	0	9	8	0	0		
14	chr4:103391275-103540762	149,488	77	1	7	7	0	0	Ulcerative colitis	.
15	chr5:148198999-148213506	14,508	42	0	35	32	0	0		Neutrophil, kidney glomeruli cell response
16	chr5:173287763-173363889	76,127	9	2	14	13	0	5	Crohn’s disease, Waist–hip ratio	.
17	chr6:135402339-135459837	57,499	27	24	26	23	7	6	Beta thalassemia/hemoglobin E disease, Cholesterol, total, F-cell distribution, HbA2 levels, Hematocrit, Hematological parameters, Hematology traits, Hodgkin's lymphoma, Mean corpuscular hemoglobin, Mean corpuscular hemoglobin concentration, Mean corpuscular volume, Mean platelet volume, Other erythrocyte phenotypes, Platelet counts, Red blood cell traits, White blood cell count, White blood cell types	CD71+, MicroRNA targets
18	chr6:139764573-139844429	79,857	48	9	73	50	13	17	Adiponectin levels, HDL cholesterol, Mean corpuscular hemoglobin, Mean corpuscular volume	CD71+, Neutrophil, MicroRNA targets
19	chr6:144190433-144674657	484,225	172	0	43	34	1	0		Neutrophil, MicroRNA targets
20	chr6:159498130-159539213	41,084	14	0	10	9	0	0		Hematopoetic cells response, Neutrophil
21	chr6:167362976-167403400	40,425	6	3	7	6	0	0	Crohn’s disease, Graves’ disease, Inflammatory bowel disease	Hematopoetic cells response
22	chr7:28716154-28874761	158,608	11	0	19	17	0	0		Neutrophil
23	chr7:33035342-33096644	61,303	5	0	12	11	0	5		.
24^a^	chr7:50366637-50417632	50,996	3	0	40	40	2	2		Dendritic cell vs monocyte response, Neutrophil
25^a^	chr7:50367656-50671350	303,695	292	6	93	82	19	17	Acute lymphoblastic leukemia (B-cell precursor), Acute lymphoblastic leukemia (childhood), Mean corpuscular volume, Red blood cell traits	CD71+, MicroRNA targets
26	chr7:106367604-106373718	6115	8	6	6	6	0	1	Mean platelet volume	.
27	chr8:61595671-61658073	62,403	10	0	6	6	0	0		
28	chr9:99087217-99192919	105,703	12	1	7	7	0	0	Height	Bone marrow progenitor cells response
29	chr9:126971204-127002414	31,211	20	0	20	16	1	0		Kidney glomeruli cells response, Neutrophil, MicroRNA targets
30	chr9:131561110-131645659	84,550	58	0	10	10	0	0		Hematopoetic cells response, Neutrophil
31	chr9:140655551-140696524	40,974	14	0	7	6	0	0		.
32	chr10:37976990-38692432	715,443	221	0	9	7	0	0		.
33	chr10:65016174-65104500	88,327	18	6	6	6	1	0	Mean platelet volume, Platelet counts, Triglycerides	Platelet
34	chr11:212408-247028	34,621	15	1	6	6	1	0	Platelet counts	Coagulation, Platelet
35	chr11:108021205-108367453	346,249	248	3	8	7	3	0	Melanoma, Response to metformin, Response to metformin in type 2 diabetes (glycemic)	Neutrophil
36	chr12:54584330-54584330	1	1	0	7	7	0	0		Genes with certain promoter motif
37^a^	chr12:54622328-54733728	111,401	6	2	7	6	0	0	Mean platelet volume, Platelet counts	Genes in band chr14q24
38^a^	chr12:54668471-54761931	93,461	27	3	29	14	2	0	Mean platelet volume, Platelet counts	Platelet
39	chr12:111833788-112896839	1,063,052	25	35	17	15	7	0	Beta-2 microglobulin plasma levels, Blood pressure, Celiac disease, Celiac disease and Rheumatoid arthritis, Cholesterol, total, Chronic kidney disease, Coronary artery disease or ischemic stroke, Coronary artery disease or large artery stroke, Coronary heart disease, Diastolic blood pressure, Eosinophil counts, Hematocrit, Hematological parameters, Hemoglobin, Hypothyroidism, Ischemic stroke, LDL cholesterol, Mean platelet volume, Platelet counts, Red blood cell traits, Retinal vascular caliber, Rheumatoid arthritis, Systolic blood pressure, Tetralogy of Fallot, Type 1 diabetes, Type 1 diabetes autoantibodies, Upper aerodigestive tract cancers, Urate levels, Vitiligo	Interferon signaling, Cytokine signaling, Immune system
40	chr12:122216910-122366612	149,703	3	5	12	12	0	0	Mean platelet volume, Platelet counts	Platelet, Neutrophil
41	chr12:129277164-129305613	28,450	56	1	17	16	0	0	Systemic lupus erythematosus	.
42	chr14:24888248-24904602	16,355	19	0	13	13	0	3		
43	chr14:35372518-35847784	475,267	360	2	18	17	1	0	Psoriasis	Pancreatic cancer cell response, Neutrophil, MicroRNA targets
44	chr16:57045349-57070617	25,269	31	0	11	11	1	0		IFNG responsive
45	chr16:87749634-87770469	20,836	6	0	9	9	0	0		
46^a^	chr16:89802396-89992238	189,843	4	14	11	8	0	0	Freckling, Hair color, Homocysteine levels, Vitiligo	.
47^a^	chr16:89919081-89993206	74,126	102	9	9	8	0	0	Basal cell carcinoma, Blond vs. brown hair color, Freckles, Hair color, Non-melanoma skin cancer, Red vs non-red hair color, Skin sensitivity to sun, Sunburns, Tanning	.
48	chr17:15899871-16239832	339,962	137	0	9	7	1	0		Beta catenin signaling, Neutrophil
49	chr17:26887271-27323322	436,052	252	0	44	41	0	11		PBMC response, CD71+, Neutrophil, MicroRNA targets
50	chr17:33763678-33819401	55,724	22	0	23	21	0	1		PBMC response, MicroRNA targets
51^a^	chr17:33823254-34067892	244,639	360	2	141	95	8	26	Platelet counts, Mean platelet volume	PBMC response, CD71+, Platelet, MicroRNA targets
52^a^	chr17:33885467-33899846	14,380	3	2	11	9	0	0	Platelet counts, Mean platelet volume	Platelet, PBMC vs. Tcells
53	chr18:43716475-43856354	139,880	121	0	10	9	0	2		
54	chr20:4139757-4188267	48,511	70	0	40	33	0	8		PBMC response, CD71+
55	chr20:62529985-62565833	35,849	54	0	9	9	0	1		Glioma cell response
56	chr21:44471469-44492843	21,375	7	3	6	6	1	0	Blood trace element (Se levels), Homocysteine levels, Obesity-related traits	.
57	chr22:32311619-32326021	14,403	17	0	8	8	0	0		Hematopoetic cell response, Neutrophil
58	chr22:49967868-50069539	101,672	128	0	18	16	0	0		
59	chrX:40802981-40847852	44,872	52	0	31	30	0	7		CD71+

^a^Overlapping clusters

*N* number of significant eQTLs in cluster, *Ng* number of GWAS SNPs in cluster, *Tr* number of transcript clusters targeted by any *trans-*eQTL in cluster, *Tr-max* max number of transcript clusters targeted by single *trans-*eQTL in cluster, *Tr-Ext* maximum number of transcripts validated in one or more of five previous results [5–8, 59–62], *Ret* number of early reticulocyte specific transcripts targeted by *trans-*eQTLs in cluster, *GSEA Category* selected GSEA themes over-represented in cluster targets, *GSEA* gene set enrichment analysis

We also identified several *trans-*eQTL clusters that target *trans-*eGenes related to other blood cell types. For example, seven clusters (17, 18, 25, 49, 51, 54, and 59; Table 5) appear to target expression of six to 27 genes specific to CD71+ early erythrocytes or reticulocytes [21] (significantly enriched, Fisher’s exact test *P* values 1.8E-33 to 1.2E-7). Of these, three (Clusters 17, 18, and 25 on chromosomes 6 and 7) contain SNPs with known associations in GWAS with red blood cell traits, including hematocrit and hemoglobin (e.g. rs668459 on chromosome 6 [22] and rs12718597 on chromosome 7 [23]). Thus, these clusters may arise from effects of the genetic variant on hematopoiesis or related pathways.

Fourteen of the clusters include eQTL-gene pairs that have been observed in previous studies [5–8] of whole blood or the peripheral blood mononuclear cells (PBMC) fraction (Table 5, Column 8), including Clusters 4, 6, 10, 11, 17, 18, 25, 29, 33, 34, 35, 38, 39, and 51. Eight of the 13 previously mentioned GWAS platelet-related clusters are among these. Another, Cluster 6, targets 18 *trans-*eGenes (seven previously validated) and contains GWAS hits for blood-related diseases and traits. Two more (Clusters 11 and 29) show enrichment of neutrophil-specific target genes. Clusters 18 and 25 include GWAS SNPs for mean corpuscular volume (e.g. rs668459 and rs12718597, respectively) and are enriched in reticulocyte specific target genes. Finally Cluster 35 with eight *trans-*eGenes, of which three are validated (Table 5, Additional file 1: Table S7), includes GWAS hits for melanoma and response to metformin, but the relationship of these eight genes to either phenotype is unclear.

### Target genes of trans-eQTL clusters may suggest mechanism of action

Clusters might arise as a result of factors other than changes in proportion of blood cell types. Examination of the sets of target genes of *trans-*eQTL clusters (Additional file 1: Table S7) may suggest a functional mechanism at play in the regulation of *trans-*eGenes. We found examples of enrichment of genes annotated as targets of transcription factors, as targets of microRNAs (miRNAs), and for several signaling pathways (Table 5, Gene set enrichment analysis (GSEA)). Some *trans-*eQTL clusters target transcripts [24] sharing a common promoter binding site motif [24], suggesting that certain transcription factor pathways are modified by genetic variants within the cluster (Additional file 1: Table S8). For example, Clusters 10 and 51 target an over-abundance of genes with promoter regions containing motifs specific to transcription factors *SP1* and *NRF1*. Indeed, transcription factor *SP1* has recently been shown to regulate platelet formation in mouse [25]. Changes in activities of these transcription factors may mediate the effect of genetic variants in these clusters on platelet formation and dynamics.

### miRNAs may mediate effects of trans-eQTLs

miRNAs that are encoded near an eQTL and bind to a *trans-*eGene might be a part of the mechanism underlying *trans-*eQTLs, as miRNAs are known to modify the expression or degradation of their target mRNAs. GSEA [26] of the genes targeted by each cluster revealed that variants in several clusters target a significant number of genes that are themselves targets of specific sets of miRNAs (at FDR < 0.05; Additional file 1: Table S9). For example, Cluster 51 targets the expression of 141 genes (Additional file 1: Tables S6 and S7) including 13 genes (*PPM1A*, *TSPAN5*, *APP*, *PIM1*, *COPS2*, *CSDE1*, *WDTC1*, *AP2A1*, *CARM1*, *FURIN*, *EPB49*, *FAM134A*, and *SH3BGRL2*) known [26] to be targets of a small set of miRNAs (miR-15A, miR-16, miR-15B, miR-195, miR-424, miR-497), a highly significant enrichment (FDR < 0.0001). Access to the measured miRNA expression data from the same whole blood samples [27] allowed us to compare the expression levels of five of these six miRNAs (miR-497 was not measured) with expression levels of the 13 genes. We found that all of these gene expression levels, except *CSDE1*, were correlated with expression levels of each of the five measured miRNAs (genome-wide FDR < 0.001). In addition, 119 of the 141 mRNA levels targeted by Cluster 51 were correlated with measured levels of at least one of these miRNAs at FDR < 0.001 (Additional file 1: Table S9). This provides suggestive evidence that one or more of these miRNAs may be involved in the mechanism of action of the corresponding genetic variants.

Cluster 39 contains SNPs associated in GWAS with almost two dozen traits or diseases (Additional file 2: Table S10) and *trans-*eQTLs targeting a similar number of distinct genes. The variant rs3184504 within *SH2B3* on chromosome 12 and its proxy rs653178 lie within this cluster and were previously observed to be *cis-*eQTLs for *SH2B3* and *trans-*eQTLs for six interferon-γ signaling transcripts and nine toll-like receptor signaling genes [5]. In our study, these two SNPs are associated with the *trans* expression levels of four of six previously reported [28] interferon-γ signaling genes and with five additional genes (*GBP3*, *GBP5*, *GBP7*, *FCGR1A*, and *FCGR1B*). We confirmed only one of nine previously reported toll-like receptor signaling genes, possibly a result of differences in the expression measurement platforms employed. Also, we found a much stronger *cis* association of rs3184504 with *ALDH2* and *OAS2* compared to *SH2B3*, although the latter harbors this eQTL in its coding region.

### Comparison to published trans-eQTL blocks or clusters

A recent study of human eQTLs in blood-derived RNA also noted extensive clusters of *trans-*eQTLs. Kirsten et al. [8] reported finding almost 849 unique *trans-*eQTLs with two or more targets, corresponding to about 175 loci. Our more restrictive definition of a *trans-*cluster requiring six or more *trans-*targets identified 753 *trans-*eQTLs in 59 loci or *trans-*clusters. However, the overlap of these two approaches was not extensive. Among our 59 *trans-*clusters of eQTLs, we found 14 harboring eQTLs also found by Kirsten et al. [8], with one or more of the identical targets (Table 5, Additional file 1: Figure S6). Of these, 12 could be readily identified as related to platelets or red blood cell components by the GWAS hits they contained. Of the remaining two clusters, Cluster 29 contains no GWAS related traits, but includes targets related to neutrophils. Cluster 35 includes GWAS hits for melanoma and response to metformin but is otherwise cryptic.

Kirsten et al. [8] highlighted ten eQTLs, each in LD with one or more GWAS hits and each with at least three, mostly novel, associated *trans-*eGenes. Of these, one (rs10512472) falls into our Cluster 51, the second strongest platelet-related cluster, for which we found 141 *trans-*eGenes including five of the nine target genes found in their study. Overall, we provide strong support for only one of their ten highlighted *trans-*clusters. This modest level of replication might be attributable to differences in the underlying cohorts, differences in the tissue RNA source, or other technical factors, or may point to platform-specific limitations in defining *trans-*clusters themselves.

### *Trans*-eQTLs not in clusters

Some of the *trans-*clusters, e.g. clusters 18 and 25, may be the direct result of variation of cell type in the whole blood samples, such as reticulocyte content, for which inadequate data were available to compensate. However, of the 5749 lead *trans-*eQTLs (Table 2), 90% (5212) are not found in any of our *trans-*clusters, suggesting that the majority of detected *trans-*eQTLs are not simply the result of uncompensated cell type variation. Rather, other mechanistic explanations should be sought, including *cis-*expression of transcription factors or miRNAs not measured in our assay, or other rarer transcribed molecules such as long non-coding RNAs having as yet unidentified effects elsewhere in the genome. Of the 5212 *trans-*eQTLs not found in clusters, 15 are found in the GWAS catalog [14] (Additional file 2: Table S10) but only a small fraction (15 of 5212 or 0.2%) were validated in earlier studies, although many were internally validated.

### Clinical relevance

Among 7057 SNPs that were associated (at *P* < 5E-8) with 942 phenotypes in the NHGRI-EBI GWAS Catalog [14], 3381 or 48% were significant eQTLs, related to 654 distinct phenotypes. This coverage represents two times the number expected by chance (1696, *P* < 2E-16, Fisher’s exact test). Limiting our results to only the lead eQTLs and variants with > 80% LD, we saw smaller but more significant coverage of 15% (observed 1028, expected 367, *P* < 2E-277). Of these 1028 lead eQTLs, 922 (or 13%), were *cis-*eQTLs; 200 (or 3%) were *trans-*eQTLs. The full list of eQTL GWAS hits is provided in Additional file 2: Table S10. The significant coverage of the GWAS Catalog makes our eQTL library valuable for exploring hypotheses regarding putative functional mechanisms.

The CARDIoGRAMplusC4D consortium completed a GWAS meta-analysis of 60,801 coronary artery disease or myocardial infarction (CAD/MI) cases and 123,504 controls and identified 58 genomic loci associated with CAD/MI [29]. Solid explanations for individual mechanisms of effect, however, were provided for only a handful of these loci. When the risk variant lies in the exon of a gene or its UTR, it is likely that the host gene is in the effect pathway. However, only four of the 58 CAD/MI GWAS SNPs reside in the exons or UTRs of genes (Additional file 1: Table S11). Two of these are missense variants (rs3184504 in *SH2B3* and rs11556924 in *ZC3HC1*). The polymorphism rs964184 lies in the 3’ UTR of *ZPR1* and rs7528419 lies in the 3’ UTR of *CELSR2* and downstream of *SORT1*. Musunuru et al. [30] demonstrated that rs12740374, a perfect proxy for the risk variant rs7528419, is responsible for changes in *SORT1* expression in liver and alters plasma LDL-cholesterol levels in mouse. For 36 CAD/MI associated loci, the lead risk variants reside in intronic regions of genes, making their contribution to the effect pathways less clear, though expression level or transcript splicing variation might play a role. For the remaining 18 loci, the lead risk variants fall tens to hundreds of kilobases from the nearest gene. Several of the nearest genes, such as *LDLR* at the 19p13.2 locus*,* encode proteins with known roles in the biology of CAD/MI, such as lipid metabolism or regulation. Others, such as the lead risk variant for the *PMAIP1-MC4R* locus are close to known obesity risk variants. Nikpay et al. [29] also noted that a cluster of such genes with documented roles in vessel wall biology can be identified among their CAD/MI GWAS results.

We posit that eQTLs can aid in identifying causal genes or pathways represented by “risk SNPs” from GWAS. Indeed, 19 of the 58 CAD/MI risk loci were previously reported by Nikpay et al. [29], Schunkert et al. [31], and the CARDIoGRAMplusC4D Consortium [32] to contain *cis-*eQTLs for nearby eGenes. The roles of several of the targeted eGenes have been confirmed in animal or in vitro experiments. For example, rs264 at 8p21.3, intronic to *LPL* (lipoprotein lipase), correlates with *LPL* expression in monocytes [32]. Mutations in *LPL* cause LPL protein deficiency resulting in type 1 hyperlipoproteinemia [33]. rs264 is also strongly associated with circulating triglyceride and HDL cholesterol levels [34].

We performed a comprehensive eQTL analysis of these 58 CAD/MI risk loci by intersecting the published GWAS SNPs with the significant eQTLs in our study and identified candidate causal genes for 21 (36%) of the risk SNPs. Eleven CAD/MI risk SNPs or a SNP in strong LD with them, were also lead eQTLs in our study. Another ten CAD/MI risk SNPs were found to be significant eQTLs, but not the lead eQTL at that locus (see “Methods”). We confirmed that ten genes at nine loci mentioned in Nikpay et al. [29] were targeted by *cis-*eQTLs, specifically at the *ABO*, *IL6R*, *LDLR*, *LPL*, *REST-NOA1*, *SORT1*, *SWAP70*, *UBE2Z*, and *VAMP5-VAMP8-GGCX* loci (Additional file 1: Table S12). These *cis-*eQTLs were highly significant, with *P* values ranging from 4 × 10^−5^ to <10^−300^ and often coincided with or were in extremely strong LD with a lead eQTL in our study. However, since our study was based on RNA derived from whole blood, failure to confirm previously observed eQTLs may stem from the tissue specificity of expression control [1].

Among the 58 GWAS SNPs for CAD/MI, we found 24 more *cis-*eQTL-eGene pairs (Additional file 1: Table S12) not mentioned by Nikpay et al. [29]. The strongest (*P* < 1E-455) eQTL, rs1412445, is in the third intron of *LIPA* (lipase A) transcript variant 1 and was a *cis-*eQTL for *LIPA* expression. This eQTL was described by Wild et al. [35] who attributed its effect on CAD through endothelial dysfunction. Lipase A catalyzes the hydrolysis of cholesteryl esters and triglycerides. Mutations can result in LAL deficiency, a disease leading to dyslipidemia and cholesteryl ester storage disease [33]. A link of LAL deficiency to premature heart disease and stroke has also been reported [36]. The second strongest of these eQTLs is rs149268645 in the WDR12 locus, a perfect proxy of the risk lead variant for the CAD/MI risk variant rs6725887. This eQTL targets *FAM117B* (*P* < 1E-80), although it is not a lead eQTL for this gene. Another perfect proxy *cis-*eQTL at this locus (rs149846585) targets expression of *CARF* (or *ALS2CR8*, *P* < 1E-40) and is the lead eQTL for that gene. The third strongest (*P* < 1E-53) eQTL, rs11191582, is in strong LD with the risk variant rs11191416 in the *CYP17A1-CNNM2-NT5C2* locus and targets the expression of *NT5C2*, although the eQTL is not the lead eQTL for that gene. *NT5C2* was recently described as a *cis-*eQTL target in the context of aneurysm susceptibility [37]*.* At this same locus, our *cis-*eQTL, rs4409766 targeting AS3MT was also found by Pierce et al. [38] in the context of arsenic metabolism. Our *cis-*eQTL rs17115100 targeting *WBP1L* was also found for this locus (Additional file 1: Table S12). We also identified potentially novel, strong *cis-*eQTLs for *SNF8* and *ATP5G1* at the *UBE2Z* locus, and *OAS2* at the *SH2B3* locus where the CAD/MI GWAS risk SNP was in very strong LD with our lead eQTL (Additional file 1: Table S12).

Two very strong *cis-*eQTLs were confirmed at the *VAMP5-VAMP8-GGCX* locus, targeting *cis-*eGenes *VAMP8* and *GGCX*. The CAD/MI risk SNP rs7568458 was in tight LD with our lead eQTL for *VAMP8* and for *GGCX*. The gene *GGCX* codes for a protein that carboxylates glutamate residues of vitamin K-dependent proteins and in turn can affect coagulation and may prevent of vascular calcification and inflammation [33]. Thus, a hypothetical causal pathway leading to inflammation may be triggered by variants at this locus, in particular through variation in one or both *cis-*eGenes. The lead CAD/MI risk SNP at the VAMP5-VAMP8-GGCX locus (rs7568458) was also in tight LD with *trans-*eQTLs targeting five eGenes (*CASP5*, *DPEP3*, *CRISPLD2*, *SLC26A8*, *PKN2*; Additional file 1: Table S12). The *trans-*eGene, *CASP5*, expression level was previously shown to be associated with blood pressure [39]. The *VAMP5-VAMP8-GGCX* locus itself coincides with our *trans* Cluster 7 (Table 5). The top GSEA term for the eGenes in this cluster was “neutrophils” (Table 5) suggesting that the *trans-*eGenes associated with this cluster are associated with altered neutrophil concentration or activity. Thus, possibly multiple causal pathways may operate here, one through *cis-*eQTL activity on *VAMP8* and *GGCX*, and another through one or more of the *trans-*eGenes such as *CASP5*.

The CAD/MI GWAS risk SNP rs3184504 at the *SH2B3* locus is in tight LD with a *cis-*eQTL targeting the expression of *OAS2* and *SH2B3*, and also is in tight LD with *trans-*eQTLs targeting 16 *trans-*eGenes (Additional file 1: Table S12). The *SH2B3* locus coincides with our *trans* Cluster 39 (Table 5). The Top GSEA categories for the 17 *trans-*eGenes in Cluster 39 include interferon signaling, cytokine signaling, and immune system (Table 5). The *SH2B3* CAD/MI GWAS risk SNP resides in a GWAS hot spot, showing strong associations with numerous diseases and phenotypes including red blood cell traits, platelet volume, and eosinophil counts, as well as CAD, blood pressure, and stroke [14]. Using the same eQTL data, Huan et al. [39] extensively studied lead variant rs3184504 in the context of blood pressure and found *SH2B3* to be a “key driver” gene of a blood pressure gene regulatory network. They found that many of the *trans-*eGenes for rs3184504 were themselves significantly related to blood pressure. It is interesting to note that one of these hypertension-related *trans-*eGenes, *ATP2B1*, is also a *cis-*eGene of the lead CAD/MI GWAS risk SNP at the *ATP2B1* locus (Additional file 1: Table S12). Thus, the pathways implicated in hypertension at the *SH2B3* locus may intersect with pathways at the *ATP2B1* locus.

We were able to confirm that *REST* is a target of a *cis-*eQTL in the *REST-NOA1* locus on chromosome 4. However, we also observed that the CAD/MI GWAS SNP at this locus, rs17087335, is in tight LD with lead *trans-*eQTLs targeting expression of *trans-*eGenes *GDAP1* (ganglioside induced differentiation associated protein 1 on chr 8; *P* < 1E-20) and *CACNA1E*. (calcium voltage-gated channel subunit alpha1 E on chr 1; *P* < 1E-7, Additional file 1: Table S12). We speculate that these *trans-*eQTLs may point to a molecular mechanism underlying this CAD/MI risk locus.

### Molecular QTL browser

**Fig. 4:**
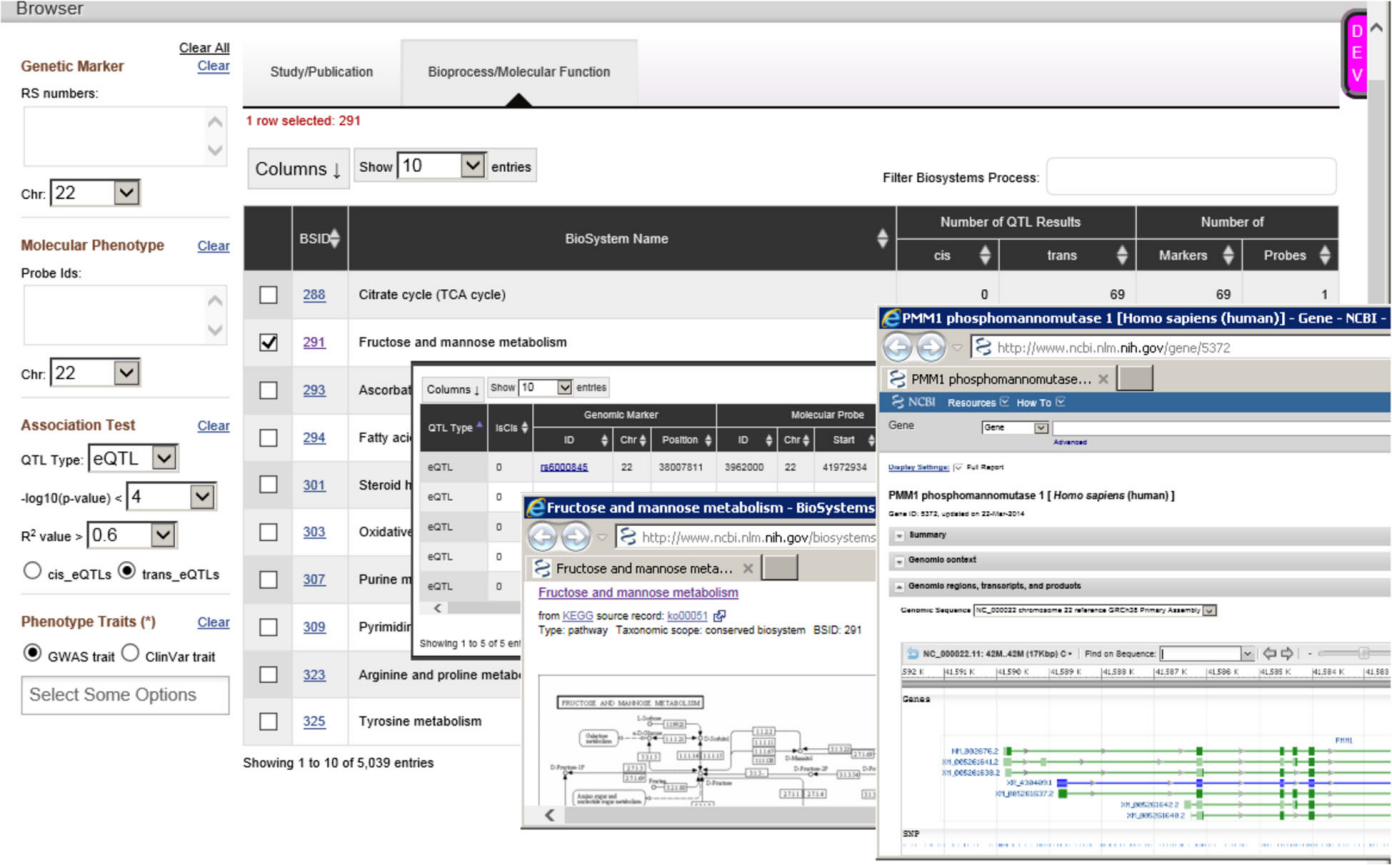
*Screenshot* of NCBI molecular QTL browser

To make our results more user-friendly and accessible, we have made them freely available via the NCBI Molecular QTL Browser (https://preview.ncbi.nlm.nih.gov/gap/eqtl/studies/), which serves as a resource for data on association between genetic variation and molecular phenotypes. The browser links our results to multiple resources including eQTLs identified in other studies. Importantly, users may specify *P* value cutoffs and other filtering criteria. Users of the Molecular QTL Browser may conduct targeted studies of specific genes based on prior evidence or may wish to do meta-analysis of multiple eQTL studies, where more permissive *P* value cutoffs may be appropriate. To support meta-analysis and other comparisons across primary studies, the integrated data resource allows for cross-dataset searches and filtering based on genome location or functional annotation (Fig. 4).

## Discussion

We provide the largest, single study and database of *cis*-eQTLs and *trans*-eQTLs to date. We considered several examples of the potential implications of our results for interpreting GWAS findings. Our results also can be used to guide functional studies such as targeted gene knockout experiments and studies of miRNA expression in follow-up of GWAS results. We have illustrated how extensive *cis-*eQTL and *trans-*eQTL data can be used to augment GWAS analysis of a complex disease (CAD/MI). Of the 58 recently reported lead risk variants for CAD/MI [29], we show that 21 contain *cis-*eQTLs targeting 34 genes. Four additional risk variants are *trans-*eQTLs targeting 24 eGenes. Thus, eQTL analysis can provide a rich resource for defining putative causal pathways of risk variants determined in GWAS.

Our genome-wide *trans-*eQTL results provide a new richness of detail regarding *trans-*eQTL clusters and their putative relations to various transcription factors and miRNA targets. Another group [8] recently also carried out a genome-wide *trans-*eQTL analysis, providing a basis for comparison of our complete *trans-*eQTL results. However, their use of a different tissue (PBMC-derived RNA rather than whole blood), use of a different expression platform (an Illumina array rather than the Affymetrix Human Exon Array), and imputation to a different SNP set (HapMap II rather than 1000 Genomes), limit the value of comparisons and explain the low rate of validation (6%).

Although many of the trans clusters may have resulted from uncompensated variation in cell type in the whole blood samples, some clusters could not be so easily explained. Moreover, a large majority (90%) of the lead *trans-*eQTLs did not appear in any cluster, including nine of our top 25 lead *trans-*eQTLs. Thus, we have identified a large number of *trans-*eQTL whose mechanism of action is likely not simply due to cell proportion, but through other mechanisms possibly involving miRNAs, transcription factors, long non-coding RNAs, or as yet unidentified transcribed molecules.

The exon-level expression data permitted us to identify more precisely cases of polymorphism-in-probe, where the genetic variant is directly detected by the expression array and might easily be interpreted as an associated change in overall gene expression. The same exon-level expression data facilitated a search for splicing variants influenced by genetic sequence (sQTLs) [40]. However, the additional noise inherent in the exon-level analysis offsets to some degree the benefits of the additional resolution offered by measuring exon-specific expression.

Our findings on *cis-*eQTL patterns are generally consistent with previous findings. We were able to validate 54–58% of our lead *cis-*eQTL results compared to other studies using microarrays. For a study using next-generation sequencing, the validation rate dropped to 25%. Only about 3–6% of our lead *trans-*eQTL results could be found in previous studies, possibly reflecting the need for very large sample size when generating *trans* results or the dependence on the specific expression platform and tissue being studied. Conversely, we were able to replicate a substantial proportion of previously published eQTL results (up to 69% for *cis* and up to 10% for *trans-*eQTL-gene pairs). Two previous studies [5, 6] have the limitation of combining, via imputation, genotypes from multiple platforms, which might lead to variation in imputation quality across SNPs. We used a single platform with approximately 550 K markers and successfully imputed 8.5 million SNPs. The study of Liang et al. [6] used two less dense genotyping platforms having approximately 100 K and 300 K markers; thus, it is not surprising that we found many more eQTLs especially in regions where our denser genotyping array provided better marker coverage. The genotype array used in the RNAseq-based study [7] was denser than our genotyping array, but the authors did not impute results to the denser 1000 Genomes SNP set. We were able both to replicate and extend the impressive findings of Westra et al. [5]. In our Cluster 39, which contains the highly pleiotropic GWAS SNP rs3184504, Westra et al. [5] observed multiple gamma interferon signaling genes and multiple toll-like receptor signaling genes as targets of this *trans-*eQTL. We were also able to identify this strong *trans-*eQTL and extend its associated transcript list to five additional interferon signaling genes.

The strengths of our study include its large sample size, expression measurement carried out in a single laboratory with rigorous quality control, use of imputation to a dense set of 1000 Genomes SNPs, and extensive attention to controlling for artifacts in the expression data. As a consequence, we found that a substantial proportion of published GWAS SNPs associated with traits or diseases are themselves lead eQTLs for nearby (13%) or distant (3%) genes. We determined that our full sample size detected 60% more target genes than did a subset of about half the original size, showing that many previously undetected eQTLs and target transcripts are probably newly detected with our study and that even more eQTL-eGene pairs remain to be discovered.

The very large proportion of variance explained (R^2^) values for the strongest eQTLs (up to 57% for *cis* and up to 22% for *trans*, Table 4), pointing to the very large influence that these variants can have on expression levels. Such high R^2^ values may also arise due to polymorphism-in-probe instances, but we used an effective procedure for detecting such cases. Of course, it is possible that as yet undiscovered SNPs exist on probes and are responsible for some of these extreme R^2^ values.

A further strength of our study is that the expression array contains far more probes and probesets than the arrays used in some other eQTL studies. For example, the array used in the meta-analysis of Westra et al. [5] (Illumina Human HT12) contains about 49,000 probesets, whereas the gene expression platform of this study, the Affymetrix Human Exon Array, contains almost six times more probesets. The additional probesets allow for the detection of expression changes along the entire length of the transcript, rather than primarily near its 3’ end. These extra probesets also give added protection against polymorphism-in-probe artifact by averaging across the many probes for each transcript.

In addition to conducting this large genome-wide eQTL study, we have created a public resource of *cis-*eQTLs and *trans-*eQTLs at the gene and exon level. Our results are based on a much larger cohort than any previous public eQTL resource, and therefore reflects a higher degree of precision and specificity of eQTLs, eGenes, and eQTL-eGene pairs.

We acknowledge several limitations of our study. The homogeneity of the FHS population may limit the applicability of our results to populations of different ancestries. Lack of population diversity might also increase the size of LD blocks and thereby limit the resolution with which true regulatory sites can be identified. Despite statistical adjustments for imputed blood cell counts, our eQTLs might still reflect cell type admixture effects and might not be comparable to results obtained in other tissues. RNAseq-based methods for determining gene expression offer even higher resolution and may not be subject to the same biases accompanying microarray measurements. However, agreement of our study with a recent RNAseq-based study [7] was comparable to the level of agreement seen with several other microarray-based studies.

## Conclusions

Despite these limitations, our results provide an extensive resource of *cis-*eQTLs and *trans-*eQTLs at the gene and exon level and this information may be useful for elucidating the biological underpinnings of many GWAS SNP associations with disease traits. Our eQTL database will facilitate better understanding of novel pathways and associations across the human genome, which may contribute to new approaches for the detection, treatment, and prevention of diseases.

## Methods

### Study participants

Recruitment procedures and clinical characteristics of participants from the FHS Offspring [10] and Third Generation cohorts [11] have been reported previously. Samples for this study came from 2770 individuals who attended the eighth Offspring cohort examination cycle (2005–2008) and 3341 individuals who attended the second examination cycle (2006–2009) of the Third Generation cohort. Protocols for participant examinations and collection of genetic materials were approved by the Boston Medical Center Institutional Review Board. All participants gave written, informed consent.

### Isolation of RNA from whole blood, preparation, and hybridization

Fasting peripheral whole blood samples (2.5 mL) from FHS participants were collected during examination in PAXgene™ tubes (PreAnalytiX, Hombrechtikon, Switzerland), incubated at room temperature for 4 h for RNA stabilization, and then stored at −80 °C. Total RNA enriched with miRNA was isolated from frozen PAXgene blood tubes by Asuragen, Inc., according to the company’s standard operating procedures for automated isolation of RNA from 96 samples in a single batch on a KingFisher® 96 robot. Tubes were allowed to thaw for 16 h at room temperature. After centrifugation and washing to collect white blood cell pellets, cells were lysed in guanidinium-containing buffer. Organic extraction was performed prior to adding binding buffer and magnetic beads in preparation for the KingFisher run. The purity and quantity of total RNA samples were determined by absorbance readings at 260 and 280 nm using a NanoDrop ND-1000 UV spectrophotometer. The integrity of total RNA was qualified by Agilent Bioanalyzer 2100 microfluidic electrophoresis, using the Nano Assay and the Caliper LabChip system.

#### Preparation of complementary DNA from RNA

RNA samples of 50 ng were amplified using the WT-Ovation Pico RNA Amplification System (NuGEN, San Carlos, CA, USA) as recommended by the manufacturer in an automated manner using the genechip array station (GCAS). In brief, first strand complementary DNA (cDNA) was prepared using a unique first strand DNA/RNA chimeric primer mix and reverse transcriptase. In the second step, DNA/RNA Heteroduplex Double Stranded cDNA was generated which served as the substrate for SPIA amplification – a linear isothermal DNA amplification process developed by NuGEN. In the third step, amplified DNA along with RNA was treated with RNase H to degrade the RNA in the DNA/RNA heteroduplex at the 5’ end of the first cDNA strand which then served as the initiation site for the next round of cDNA synthesis. The process of SPIA DNA/RNA primer binding, DNA replication, strand displacement, and RNA cleavage is repeated, resulting in rapid accumulation of microgram amounts of SPIA cDNA. An aliquot of the SPIA cDNA was used for quantitative polymerase chain reaction (qPCR) analysis.

#### Target labeling and hybridization onto Affymetrix Genechips

Three micrograms of the amplified cDNA from the WT-Ovation Pico amplification step were processed with the WT-Ovation Exon Module in GCAS to produce sense strand ST-cDNA following the manufacturer’s (NuGEN, San Carlos, CA, USA) procedure; 5 μg ST-cDNA was fragmented and labeled with the FL-Ovation™ cDNA Biotin Module using a proprietary two-step fragmentation and labeling process. The first step is a combined chemical and enzymatic fragmentation process that yields single-stranded cDNA products in the base range of 50–100 . In the second step, this fragmented product is labeled via enzymatic attachment of a biotin-labeled nucleotide to the 3-hydroxyl end of the fragmented cDNA generated in the first step. Hybridization, washing, and laser scanning of Affymetrix Human Exon 1.0 ST microarrays were performed according to the manufacturer’s protocol (Affymetrix, Santa Clara, CA, USA). Hybridization was performed at 45 °C overnight, followed by washing and staining using FS450 fluidics station. Scanning was carried out using the 7G GCS3000 scanner.

### Affymetrix human exon 1.0 ST microarray platform

This platform consists of approximately 6 million 25 base probes, grouped into about 300,000 four-probe probesets, each designed to target an exon of a transcript. Multiple probesets are grouped together to represent a set of transcripts from a single gene (called a transcript cluster). Transcript clusters are annotated to genes in a nearly one-to-one fashion. Transcript clusters have from one to several hundred probesets, depending on the length of the transcript, and form the basis of our analysis here. Of the 12,396 transcript cluster IDs which are found to be eGenes (either *cis-* or *trans-*), only 244 are in a many-to-one relationship with an EntrezGene and 282 no longer map to an Entrez gene entry. Thus, a transcript cluster level analysis may be considered a proxy for a gene-level analysis.

### Microarray data collection, quality control, and data adjustment

The intensity values for each gene chip were collected using the robust multi-chip average (RMA) method available in the Affymetrix Power Tools (APT) [41] Software version 1.12.0 (Affymetrix). A total of 287,329 Refseq-core [42] probesets representing 17,873 distinct genes from 6111 samples were extracted from the APT, based on NetAffx annotation version 31 [43]. Samples were excluded based on three factors: (1) values for a quality control (QC) metric, all_probeset_rle_mean ≥ 0.7 [44]; (2) chromosome Y-linked gene expression did not agree with reported sex; and (3) when a DNA/mRNA sample pair mix-up is apparent, based on the top 395 eQTLs with minor allele frequency (MAF) ≥ 0. The remaining 5626 samples with satisfactory results constituted the study samples and were again normalized with RMA, retaining only core-level probesets. We determined that many artifacts in the expression data could be reduced by adjusting for chipping batch, various technical factors provided by Affymetrix APT program for each array hybridization, and for the first principal component (PC1) determined from the centered and unscaled RMA data. The technical adjustment factors were: all_probeset_mean, all_probeset_stdev, neg_control_mean, neg_control_stdev, pos_control_mean, pos_control_stdev. all_probeset_rle_mean, all_probeset_mad_residual_mean, and mm_mean. In addition, we adjusted by ProbesetGroupDiff, which partially accounts for the non-random layout of probes on the Affymetrix Exon Array.

Several additional data adjustments were considered beyond the technical covariates described above. We tested the effects of: (1) including 40 PCs on the unadjusted data; (2) including 20 PEER factors [45] on the unadjusted data; (3) including 20 PEER factors on the adjusted data; and (4) including 40 surrogate variables [46] on the un-adjusted data. The internal validation rate for *cis-*eQTLs (Additional file 1: Table S2) was greatest when 20 PEER factors were used with the adjusted data and this approach was selected as the method of choice.

### Genotyping platform and SNP imputation

Of the 5626 microarray samples passing quality controls, 5257 were previously genotyped using the Affymetrix 500 K and MIPS 50 K platforms [47, 48]. From a total number of 549,781 genotyped SNPs, we removed 137,728 genotyped SNPs on the following filtering criteria: Hardy–Weinberg Equilibrium (HWE) *P* value < 1E-6 (22,018 SNPs), call rate < 96.9% (48,285 SNPs), MAF < 0.01 (66,063 SNPs), map mismatch from Build 36 to Build 37 (82 SNPs), missing a physical location (428 SNPs), number of Mendelian errors > 1000 (25 SNPs), residing outside of chromosomes 1–22 or X (786 SNPs), and duplicates (41 SNPs). This leaves the remaining 412,053 SNPs as input to Minimac [49], an implementation of genotype imputation software, MACH [50]. The 1000-Genomes “cosmopolitan” SNP set [51] was used as the imputation reference platform. Minimac’s GIANT 1000 Genomes Imputation protocol was used, with the SNP phasing options of: −rounds 20 –states 200 –phase –sample 5, yielding a total of 39,315,185 SNPs. Of these, we chose SNPs with imputed quality score (R^2^) ≥ 0.3 and MAF ≥ 0.01, leaving a total of 8,510,936 SNPs for analysis of *cis* and *trans* association, all in hg19 coordinates. The genotyping data are available in dbGaP under study phs000342.v13.p9 (http://www.ncbi.nlm.nih.gov/projects/gap/cgi-bin/study.cgi?study_id=phs000342), which is under the umbrella of the overall FHS study of phs000007.

We performed a principal component analysis (PCA) with 521 unrelated FHS participants (Additional file 1: Figure S7) along with HapMap individuals (CEPH with Northern and Western European ancestry (CEU, Pink), Yoruba from Ibadan, Nigeria (YRI, Red), Han Chinese from Beijing (CHB), and Japanese from Tokyo (JPT). The samples which entered the eQTL study are shown in Additional file 1: Figure S7. The program *smartpca* from the EIGENSOFT package was used to perform the PCA [52]. PCs for additional FHS participants were computed from the PC weights derived from that analysis.

We also charted the effect of imputation R^2^ on validation rates for that eQTL (Additional file 1: Figure S8). Validation rate rises nearly linearly with imputation R^2^ from about 58% (R^2^ < 35%) to ~69% (R^2^ > 85%). However, since the overall average imputation R^2^ for this set is 94%, the lowered validation rate for lower quality imputation has little impact on the final result.

### Whole blood cell counts

Of the 5257 samples, 2181 from the Third Generation cohort had whole blood complete blood cell counts (CBCs, Beckman Coulter, Brea, CA, USA). The cell counts of the remaining samples were imputed using a partial least squares (PLS) prediction based on the gene expression data. Cross-validated estimates of prediction accuracy (R^2^) for the CBC components (WBC, RBC, platelet, neutrophil percent, lymphocyte percent, monocyte percent, eosinophil percent, and basophil percent) were 0.61, 0.41, 0.25, 0.83, 0.83, 0.81, 0.89, and 0.25, respectively. We conducted comparisons between results of using imputed cell counts and those when using measured ones and did not find significant difference. Thus, we used measured cell counts when available and imputed ones when not.

### Statistical analysis

An eQTL is defined to be any SNP with a significant association to the expression level of some transcript.

The analysis required two phases: first, using the mixed-effect modeling package pedigreemm [53] of R version 3.0.1, we removed from the expression data (for 5626 samples) the effects of sex, age, platelet count, white blood cell whole count, and imputed differential count (percentages of lymphocytes, monocytes, eosinophils, and basophils), while accounting for reported familial relationships, and collected the residuals. Next, we computed 20 factors using a Bayesian framework to infer hidden confounding factors (PEER [45]) on the residualized gene expression data. These PEER factors, along with sex, age, and imputed effect allele dosage were used fit to the ResidualizedExpression, in an additive linear model for the 5257 samples:

$$ \mathrm{ResidualizedExpression} = \mathrm{Mean} + \mathrm{Sex} + \mathrm{Age} + \mathrm{Peer}1 + \dots + \mathrm{Peer}20 + \mathrm{EffectAlleleDosage} $$

The model fit was repeated for all 1.5 × 10^11^ SNP:transcript cluster pairs. The algorithm was implement using Graphical Processing Units (GPUs) to accelerate the computation. We collected the effect estimate (β), T-statistics, R^2^, log10 *P* values, and log10 of Benjamini–Hochberg’s [54] FDR for EffectAlleleDosage, after accounting for the other covariates, for each association with *P* values < 1E-4. The FDR computations for *cis* and *trans* were performed separately. To check for the influence of possible apparent inflation of *P* values in just the 2 Mb cis regions, we used the method of Devlin and Roeder [12] to adjust the *P* values such that the genomic control factor λ becomes 1.0. The FDR values for the declared significant cis-eQTLs rose only slightly, but did not exceed the stated cutoff of 5%.

### Enrichment *P* value calculation

In calculating “enrichment,” i.e. observed number divided by expected number, we accounted for the LD structure of the available 8.5 million SNPs by first “pruning” to obtain a set of independent SNPs. Expected numbers were obtained from the relevant 2 × 2 contingency tables, using the pruned set as the basis of comparison, thereby insuring that counts were from nearly independent observations. The pruning was accomplished by first ordering SNPs by the minimal *P* value of that SNP with any gene in the eQTL database, followed by all remaining insignificant SNPs. Starting with the first SNP on the list, we prune subsequent SNPs with LD > 0.3. We then consider the second remaining list member and prune the rest of the list, and so forth until we reach the end of the list. The LD was computed between pairwise SNPs within the FHS dataset. This resulted in a set of about 279,310 independent SNPs.

### Definition of *cis-*eQTL, *trans-*eQTL, primary lead eQTL, and secondary lead eQTLs

An SNP-transcript cluster pair is considered *cis* if the SNP resides within 1 Mb of the TSS on the same chromosome or if the SNP resides in a contiguous block of eQTLs which includes the TSS. A contiguous block of eQTLs targeting a single transcript cluster is a set of significant eQTLs on the same chromosome with no internal gaps greater than 1 Mb. Such blocks ranged in size up to 10 Mb. eQTLs that fall in blocks which did not contain the TSS for its target transcript cluster were defined as “trans.” The “lead eQTL” is the strongest eQTL, judged by *P* value for association, in its block. A secondary lead eQTL may be found for a particular block (and particular transcript) by fitting the regression model:

$$ \begin{array}{c}\mathrm{ResidualizedExpression} = \mathrm{Mean} + \mathrm{Sex} + \mathrm{Age} + \mathrm{Peer}1 + \dots + \mathrm{Peer}20\\ {} + \mathrm{EffectAlleleDosage}\left(\mathrm{primary}\ \mathrm{lead}\ \mathrm{eQTL}\right)\kern0.5em \\ {} + \mathrm{EffectAlleleDosage}\left(\mathrm{test}\ \mathrm{SNP}\right)\end{array} $$

for each test SNP in the current block that has low LD correlation to the primary lead eQTL (R^2^ < 0.36). If the *P* value for the coefficient of the best test eQTL is less than 0.0001, we define this as a secondary lead eQTL. This process is iterated, adding successive secondary lead eQTLs to the regression model until no more low-linkage SNPs are left or the *P* value of the test SNP is above 0.0001. This method is similar to one suggested by Powell et al., but allows the effects of all eQTLs to be re-estimated at each step of the regression [55].

### Internal replication analysis

Results were replicated in two phases: (1) internal replication using two subgroups within the FHS overall study set; and (2) external replication based on published eQTL datasets. In the internal replication stage, we used the FHS Offspring cohort as the discovery dataset and the FHS Third Generation cohort as the replication dataset. We ran the exact same statistical analysis on each dataset and required that the pair satisfy FDR < 5% in both the discovery and replication datasets to be considered replicated. Since both datasets used the same platforms for genotyping and expression, matching markers and transcripts (or probesets) were done directly using the marker ID or the transcript cluster ID.

### External replication and validation analysis

Calculation of external replication rates required that the eligibility of a published eQTL-transcript cluster pair be determined. For Westra et al. [5] and Liang et al. [6], we matched markers reported in those studies by their identifiers or their exact genomic positions, to the available markers in our study. We matched their transcript probes by overlap of their exact genomic start and end addresses with the Affymetrix transcript clusters. Replication rate, then, is the ratio of previously reported results that are found in our study to the previous results eligible to be replicated in our study. For Battle et al. [7], only gene-symbol not genomic location was reported, which was matched to the annotated gene-symbol for the Affymetrix transcript cluster. For Kirsten et al. [8], transcripts were defined by Entrez IDs, which were matched to the annotation of the Affymetrix transcript cluster ID.

Calculation of validation rates starts with determination of which of our eQTLs and which of our transcripts were eligible to be measured in the published study. Validation rate is the number of eligible lead eQTL-transcript cluster pairs in our results which are also validated in the published study divided by the number of eligible pairs. Validation is asserted when we can find a SNP:transcript cluster pair in the external study where the SNP has > 80% LD correlation with our lead eQTL. For Multiple Studies [2, 4–6], we considered a pair to be validated if it was validated by any one of the studies. Since we did not have access to a complete list of SNPs or of transcripts, measurement of which passed quality control in each external study, we had to make some assumptions about eligibility. To be eligible, we required the SNP Rs ID or the SNP genomic address to match exactly, and that the SNP be included in HapMap version 3. We also required that the probes of the transcript on the external study to overlap the probes on the Affymetrix transcript cluster, without regards to the annotated gene-symbols. More details are included in Additional file 1: Supplementary Methods. For Battle et al. [7] eligibility for validation was determined if the SNP was on the Illumina HumanOmni1-Quad_v1 BeadChip which interrogated 1,124,584 SNPs and the transcript was part of the NCBI v37.2 (a.k.a., hg18) *H. sapiens* reference genome. Since Battle et al. [7] reported only the lead-eQTL per transcript, we defined validation if our lead eQTL was in LD with theirs at R^2^ > 80%. For Kirsten et al. [8] eligibility was derived from a file (personal communication from H. Kirsten, 8/31/2016) of which SNPs and which EntrezIDs were used by them in detecting significant SNP-transcript cluster pairs. We also asserted validation if our lead eQTL was in LD with R^2^ > 80% with their results.

Our definition of validation did not consider the direction of change because many studies did not report that direction or did not report which allele was considered as the reference. Kirsten et al. [8] did report sufficient information to make this comparison, however. We reported the mean percentage agreement for all validated lead eQTL-transcript cluster pairs or pairs where the eQTL was in > 80% LD with our lead eQTL.

To determine expected numbers of validated pairs under the random assumption, each study calculated the ratio of number of eligible detected pairs to the possible number of eligible *cis-*eQTL:transcript pairs in the external study. Then, since our lead eQTLs were independent, we multiplied this ratio by the number of eligible pairs to be validated. *P* values for the overlap of ours and previous studies were calculated from 2 × 2 contingency tables, separately for *cis-* and *trans-*. In every case, the *P* values based on Fisher’s exact test were incalculably small and were not separately reported.

Detection rates and validation rates rose with the number of probesets available for each transcript or transcript cluster (see Additional file 2: Table S10), reaching a plateau when about 21 to 25 probesets were available. A probeset consists generally of four 25 base probes on the Affy Exon array. A transcript cluster consists of from one to several hundred probesets. Relative validation rates also rose with increasing expression level (Additional file 1: Figure S9), suggesting that more highly expressed genes are more reliably detected as targets of eQTLs.

### Polymorphism-in-probe analysis

When a SNP appears in the microarray probe, it may appear to modify the expression level of that gene, but actually only modify the binding affinity of the RNA to the probe itself. The Affymetrix Human Exon array is uniquely suited to detecting this artifact since it includes multiple, typically ten, probesets per gene. SNPs affecting the binding affinity at a single probe are unlikely to affect the affinity at other probes, so artifactual expression changes can be detected by comparing exon-level expression to that of the entire gene. We developed a rule to distinguish artifactual from real eSNPs as follows. A SNP located in an Affymetrix probe was declared to be a likely artifactual cause of significance if: (1) the association R^2^ for this SNP was high, greater than 90% of the maximal R^2^ achieved by the lead eQTL, in the gene-level analysis; and (2) the R^2^ in the exon-level analysis for this SNP for its exon was greater than 95% of the maximal R^2^ achieved by any *cis-*SNP for any of the exons in this transcript cluster. In such cases, all eQTLs for this transcript cluster (gene) were marked as likely artifact since most would be in linkage disequilibrium to some extent, with the lead eQTL.

We downloaded the golden path track from the UCSC Genome website for the Affymetrix Exon Array probes, probesets, and transcript cluster addresses in hg19 coordinates. We performed an overlap analysis between the probe coordinates and the addresses of SNPs with imputed quality score (R^2^) ≥ 0.3 and MAF ≥ 0.01. We counted the number of eQTL pairs and the number of SNPs with such an overlap.

### Determination of the genomic control factor

The computation of the genomic control factor would have required storage of at least half of all results, which we estimated at about 4 Petabytes for our dataset. Due to such extensive storage requirements, we rather opted to perform transcriptome-wide eQTL analysis on a random subsample of 100,000 SNPs at the gene level (17,873 transcript clusters). The SNPs were selected from those with imputed quality score (R^2^) ≥ 0.3 and MAF ≥ 0.01, and also within the HapMap SNP set. We stored only the *P* values arising from this analysis. We computed the genomic control factor λ as defined by Devlin and Roeder [12]. Let F(∙) be the upper-tail cumulative distribution function of χ^2^ with degrees of freedom of 1. Then λ = F^−1^(median(*P* values))/F^−1^(0.5).

### Intersection with NHGRI GWAS catalog

We downloaded NHGRI GWAS catalog [14] on 5 June 2016 and filtered out SNP-trait pairs with *P* values > 5e-8, leaving 7057 unique SNPs covering 942 phenotypes. We intersected the GWAS SNPs with our significant eQTLs having FDR < 0.05 and with our lead eQTLs.

### *Trans*-eQTL cluster definition

*Trans-*eQTLs sometimes appeared in narrow blocks or clusters within the genome, affecting numerous distant transcript clusters. To formally define these clusters, we focused only on all SNPs having six or more *trans* associations and excluded all associations that resided in the same chromosome as the SNP. We use a modified K nearest-neighbor (KNN) algorithm [56] as follows. Starting with the lead *trans-*eQTL, i.e. the SNP with the most significant association by *P* value as a centroid, we considered successive eQTLs to the left and the right of the starting SNP (but on the same chromosome) and determined whether to include each new eQTL in the growing cluster according to its “distance” from the cluster. Let *A* be the set of eGenes targeted by the current set of *trans-*eQTLs and *B* be the set of target eGenes of the neighboring eQTL. We computed the distance *d* between sets *A* and *B*, where *d* = 1 - |A∩B|/min(|A|,|B|), where |.| denotes the size of the set. If *d* < 0.7, we combined the neighboring eQTL with the current set of eQTLs into the cluster. Once there are no further SNPs passing the distance cutoff, the eQTLs in the current cluster were recorded and the algorithm restarted with the next available eQTL in the original chromosome not yet included in clusters. The clustering process is iterated until all SNPs on the original chromosome were considered. The clustering process builds a set or block of nearby SNPs which are *trans-*eQTLs for substantially the same set of genes.

### Gene-set enrichment analysis

We performed GSEA [26] to determine putative functions of the genes of each trans-cluster. We used the online “Investigate Gene Sets platform GSEA” at http://software.broadinstitute.org/gsea/msigdb/annotate.jsp, which computes overlaps of the submitted gene lists with a library of pre-established gene lists and provides a significance indicator for the degree of overlap. We selected all categories (C1: positional gene sets, C2: curated gene sets, C3: motif gene sets, C4: computational gene sets, C5: GO gene sets, C6: oncogenic signatures, C7: immunologic signatures) and collected all categories with FDR < 0.05. We separated the categories that correspond to promoters, transcription factors, and miRNA targets.

### Enrichment analysis for CD71+ genes

We gathered from the literature 166 gene symbols that are known to be associated with the CD71+, early erythrocytes, or reticulocyte transcript [21]. We performed one-sided Fisher’s exact test to test for enrichment only on clusters targeting six or more genes in common with these 166 genes.

### MiRNA data collection

The profiling of the miRNA expression, as described in a previous study [27], was performed using the quantitative real-time polymerase chain reaction (qRT-PCR) using the same PAXgene Blood RNA samples from the same set of individuals as in the mRNA expression profiling. The qRT-PCR was performed using a high throughput qRT-PCR instrument BioMark System (Fluidigm, South San Francisco, CA, USA). Blanking was performed for quality control purposes using the BioMark dynamic array platform and pooled samples were repeatedly measured for chip to chip variability, showing excellent reproducibility and no cross-contamination. Threshold cycle (Ct) values as measured by the qRT-PCR instrument were used as measurements of miRNA expression levels. Since Ct values reflect the number of amplification cycles required for the fluorescent signal to exceed the background level, low Ct values indicate higher expression of miRNA, with values over 27 considered as missing due to the possible oversaturation of PCR product.

### MiRNA-mRNA co-expression analysis

The log-2 transformed miRNA Ct values were normalized and adjusted for isolation batch, RNA concentration, RNA quality, and 260/280 ratio (defined as the ratio of the absorbance at 260 and 280 nm; measured using a spectrophotometer). The co-expression analyses between mRNA expression levels and miRNA levels were performed under linear mixed model, adjusting for age, sex, and family structure, using the lmekin function in the kinship package [57], on samples with both miRNA and mRNA (n up to 5357). We excluded miRNA measured in fewer than 400 non-missing values. Genome-wide Benjamini and Hochberg’s [54] FDR was used to correct for multiple comparisons. Only results with genome-wide FDR < 0.001 were considered. The miRNA-mRNA co-expression database is described in Huan et al. [27].

### Cluster miRNA enrichment analysis

To obtain miRNA targets per cluster, we performed GSEA analysis (on miRNA target category) on transcripts targeted by each cluster. We filtered the GSEA results at FDR < 0.05. GSEA may output multiple miRNAs for one cluster. After the GSEA analysis, we confirmed our findings to see if the miRNA-transcript cluster pair are indeed observed in our miRNA-mRNA co-expression database above. We reported the number of confirmed transcripts per cluster.

## Additional files

Additional file 1: Supplementary Materials, including nine figures, Supplementary Methods, and 12 tables. (PDF 1844 kb)

Additional file 2: Table S10. GWAS-associated *cis*-eQTLs and *trans*-eQTLs, that overlap with the NHGRI GWAS catalog (downloaded on 5 June 2016, filtered by association *P* < 5E-8). **Table S10a.** Overlaps with lead eQTLs or with > 80% R2 of lead eQTL. **Table S10b.** Overlaps with all significant eQTLs. (XLSX 1541 kb)

## References

[1] GTEx Consortium (2013). The Genotype-Tissue Expression (GTEx) project. Nat Genet.

[2] ENCODE Project Consortium (2012). An integrated encyclopedia of DNA elements in the human genome. Nature.

[3] Eicher JD, Landowski C, Stackhouse B, Sloan A, Chen W, Jensen N (2015). GRASP v2.0: an update on the Genome-Wide Repository of Associations between SNPs and phenotypes. Nucleic Acids Res.

[4] Fehrmann RSN, Jansen RC, Veldink JH, Westra HJ, Arends D, Bonder MJ (2011). Trans-eQTLs reveal that independent genetic variants associated with a complex phenotype converge on intermediate genes, with a major role for the HLA. PLoS Genet.

[5] Westra H-J, Peters MJ, Esko T, Yaghootkar H, Schurmann C, Kettunen J (2013). Systematic identification of trans eQTLs as putative drivers of known disease associations. Nat Genet.

[6] Liang L, Morar N, Dixon AL, Lathrop GM, Abecasis GR, Moffatt MF (2013). A cross-platform analysis of 14,177 expression quantitative trait loci derived from lymphoblastoid cell lines. Genome Res.

[7] Battle A, Montgomery SB (2014). Determining causality and consequence of expression quantitative trait loci. Hum Genet.

[8] Kirsten H, Al-Hasani H, Holdt L, Gross A, Beutner F, Krohn K (2015). Dissecting the genetics of the human transcriptome identifies novel trait-related trans-eQTLs and corroborates the regulatory relevance of non-protein coding loci†. Hum Mol Genet.

[9] Zhang X, Gierman HJ, Levy D, Plump A, Dobrin R, Goring HH (2014). Synthesis of 53 tissue and cell line expression QTL datasets reveals master eQTLs. BMC Genomics.

[10] Feinleib M, Kannel WB, Garrison RJ, McNamara PM, Castelli WP (1975). The Framingham Offspring Study. Design and preliminary data. Prev Med.

[11] Splansky GL, Corey D, Yang Q, Atwood LD, Cupples LA, Benjamin EJ (2007). The Third Generation Cohort of the National Heart, Lung, and Blood Institute’s Framingham Heart Study: design, recruitment, and initial examination. Am J Epidemiol.

[12] Devlin B, Roeder K (1999). Genomic control for association studies. Biometrics.

[13] Ramasamy A, Trabzuni D, Gibbs JR, Dillman A, Hernandez DG, Arepalli S (2013). Resolving the polymorphism-in-probe problem is critical for correct interpretation of expression QTL studies. Nucleic Acids Res.

[14] Hindorff LA, Sethupathy P, Junkins HA, Ramos EM, Mehta JP, Collins FS (2009). Potential etiologic and functional implications of genome-wide association loci for human diseases and traits. Proc Natl Acad Sci U S A.

[15] Raghavachari N, Xu X, Harris A, Villagra J, Logun C, Barb J (2007). Amplified expression profiling of platelet transcriptome reveals changes in arginine metabolic pathways in patients with sickle cell disease. Circulation.

[16] Boyle AP, Hong EL, Hariharan M, Cheng Y, Schaub MA, Kasowski M (2012). Annotation of functional variation in personal genomes using RegulomeDB. Genome Res.

[17] Joo JWJ, Sul JH, Han B, Ye C, Eskin E (2014). Effectively identifying regulatory hotspots while capturing expression heterogeneity in gene expression studies. Genome Biol.

[18] Westra H-J, Franke L (1842). From genome to function by studying eQTLs. Biochim Biophys Acta.

[19] Eicher JD (2016). Characterization of the platelet transcriptome by RNA sequencing in patients with acute myocardial infarction. Platelets.

[20] Simon LM, Edelstein LC, Nagalla S, Woodley AB, Chen ES, Kong X (2014). Human platelet microRNA-mRNA networks associated with age and gender revealed by integrated plateletomics. Blood.

[21] Wu C, Orozco C, Boyer J, Leglise M, Goodale J, Batalov S (2009). BioGPS: an extensible and customizable portal for querying and organizing gene annotation resources. Genome Biol.

[22] Li J, Glessner JT, Zhang H, Hou C, Wei Z, Bradfield JP (2013). GWAS of blood cell traits identifies novel associated loci and epistatic interactions in Caucasian and African-American children. Hum Mol Genet.

[23] Ganesh SK, Zakai NA, van Rooij FJ, Soranzo N, Smith AV, Nalls MA (2009). Multiple loci influence erythrocyte phenotypes in the CHARGE Consortium. Nat Genet.

[24] Xie X, Lu J, Kulbokas EJ, Golub TR, Mootha V, Lindblad-Toh K (2005). Systematic discovery of regulatory motifs in human promoters and 3’ UTRs by comparison of several mammals. Nature.

[25] Meinders M, Kulu DI, van de Werken HJ, Hoogenboezem M, Janssen H, Brouwer RW (2015). Sp1/Sp3 transcription factors regulate hallmarks of megakaryocyte maturation and platelet formation and function. Blood.

[26] Subramanian A, Tamayo P, Mootha VK, Mukherjee S, Ebert BL, Gillette MA (2005). Gene set enrichment analysis: a knowledge-based approach for interpreting genome-wide expression profiles. Proc Natl Acad Sci U S A.

[27] Huan T, Rong J, Liu C, Zhang X, Tanriverdi K, Joehanes R (2015). Genome-wide identification of microRNA expression quantitative trait loci. Nat Commun.

[28] Croft D, Mundo AF, Haw R, Milacic M, Weiser J, Wu G (2014). The Reactome pathway knowledgebase. Nucleic Acids Res.

[29] Nikpay M, Goel A, Won HH, Hall LM, Willenborg C, Kanoni S (2015). A comprehensive 1,000 Genomes-based genome-wide association meta-analysis of coronary artery disease. Nat Genet.

[30] Musunuru K, Strong A, Frank-Kamenetsky M, Lee NE, Ahfeldt T, Sachs KV (2010). From noncoding variant to phenotype via SORT1 at the 1p13 cholesterol locus. Nature.

[31] Schunkert H, Konig IR, Kathiresan S, Reilly MP, Assimes TL, Holm H (2011). Large-scale association analysis identifies 13 new susceptibility loci for coronary artery disease. Nat Genet.

[32] Deloukas P, Kanoni S, Willenborg C, Farrall M, Assimes TL, CARDIoGRAMplusC4D Consortium (2013). Large-scale association analysis identifies new risk loci for coronary artery disease. Nat Genet.

[33] Pruitt K, Brown G, Tatusova T, Maglott D (2002). The National Center for Biotechnology Information Handbook.

[34] Smith AJP, Palmen J, Putt W, Talmud PJ, Humphries SE, Drenos F (2010). Application of statistical and functional methodologies for the investigation of genetic determinants of coronary heart disease biomarkers: lipoprotein lipase genotype and plasma triglycerides as an exemplar. Hum Mol Genet.

[35] Wild PS, Zeller T, Schillert A, Szymczak S, Sinning CR, Deiseroth A (2011). A genome-wide association study identifies LIPA as a susceptibility gene for coronary artery disease. Circ Cardiovasc Genet.

[36] Reiner Ž, Guardamagna O, Nair D, Soran H, Hovingh K, Bertolini S (2014). Lysosomal acid lipase deficiency--an under-recognized cause of dyslipidaemia and liver dysfunction. Atherosclerosis.

[37] Awad AJ, Bederson JB, Mocco J, Raj T (2016). Expression quantitative trait locus analysis from primary immune cells identifies novel regulatory effects underlying intracranial aneurysms susceptibility. Neurosurgery.

[38] Pierce BL, Kibriya MG, Tong L, Jasmine F, Argos M, Roy S (2012). Genome-wide association study identifies chromosome 10q24.32 variants associated with arsenic metabolism and toxicity phenotypes in Bangladesh. PLoS Genet.

[39] Huan T, Meng Q, Saleh MA, Norlander AE, Joehanes R, Zhu J (2015). Integrative network analysis reveals molecular mechanisms of blood pressure regulation. Mol Syst Biol.

[40] Tryka KA, Hao L, Sturcke A, Jin Y, Wang ZY, Ziyabari L (2014). NCBI’s Database of Genotypes and Phenotypes: dbGaP. Nucleic Acids Res.

[41] Affymetrix. *Affymetrix Power Tools*. (Affymetrix).

[42] Pruitt KD, Brown GR, Hiatt SM, Thibaud-Nissen F, Astashyn A, Ermolaeva O (2014). RefSeq: an update on mammalian reference sequences. Nucleic Acids Res.

[43] Affymetrix (2006). Transcript assignment for NetAffx(TM) Annotations.

[44] Joehanes R, Johnson AD, Barb JJ, Raghavachari N, Liu P, Woodhouse KA (2012). Gene expression analysis of whole blood, peripheral blood mononuclear cells, and lymphoblastoid cell lines from the Framingham Heart Study. Physiol Genomics.

[45] Stegle O, Parts L, Durbin R, Winn J (2010). A bayesian framework to account for complex non-genetic factors in gene expression levels greatly increases power in eQTL Studies. PLoS Comput Biol.

[46] Leek JT, Storey JD (2007). Capturing heterogeneity in gene expression studies by surrogate variable analysis. PLoS Genet.

[47] Cupples LA, Arruda HT, Benjamin EJ, D’Agostino RB, Demissie S, DeStefano AL (2007). The Framingham Heart Study 100 K SNP genome-wide association study resource: overview of 17 phenotype working group reports. BMC Med Genet.

[48] Karasik D, Dupuis J, Cho K, Cupples LA, Zhou Y, Kiel DP (2010). Refined QTLs of osteoporosis-related traits by linkage analysis with genome-wide SNPs: Framingham SHARe. Bone.

[49] Howie B, Fuchsberger C, Stephens M, Marchini J, Abecasis GR (2012). Fast and accurate genotype imputation in genome-wide association studies through pre-phasing. Nat Genet.

[50] Li Y, Willer CJ, Ding J, Scheet P, Abecasis GR (2010). MaCH: using sequence and genotype data to estimate haplotypes and unobserved genotypes. Genet Epidemiol.

[51] McVean GA, Altshuler DM, Durbin RM, Abecasis GR, Bentley DR, Chakravarti A (2012). An integrated map of genetic variation from 1,092 human genomes. Nature.

[52] Price AL, Butler J, Patterson N, Capelli C, Pascali VL, Scarnicci F (2008). Discerning the ancestry of European Americans in genetic association studies. PLoS Genet.

[53] Vazquez AI, Bates DM, Rosa GJM, Gianola D, Weigel KA (2010). Technical note: an R package for fitting generalized linear mixed models in animal breeding. J Anim Sci.

[54] Benjamini Y, Hochberg Y (1995). Controlling the false discovery rate: a practical and powerful approach to multiple testing. J R Stat Soc Ser B Methodol.

[55] Powell JE, Henders AK, McRae AF, Kim J, Hemani G, Martin NG (2013). Congruence of additive and non-additive effects on gene expression estimated from pedigree and SNP data. PLoS Genet.

[56] Altman NS (1992). An introduction to kernel and nearest-neighbor nonparametric regression. Am Stat.

[57] Therneau TM, Atkinson B. Kinship package version 1.1.3.

[58] Zhang X, Joehanes R, Chen BH, Huan T, Ying S, Munson PJ (2015). Identification of common genetic variants controlling transcript isoform variation in human whole blood. Nat Genet.

[59] Schadt EE, Molony C, Chudin E, Hao K, Yang X, Lum PY (2008). Mapping the genetic architecture of gene expression in human liver. PLoS Biol.

[60] Montgomery SB, Sammeth M, Gutierrez-Arcelus M, Lach RP, Ingle C, Nisbett J (2010). Transcriptome genetics using second generation sequencing in a Caucasian population. Nature.

[61] Gibbs JR, van der Brug MP, Hernandez DG, Traynor BJ, Nalls MA, Lai SL (2010). Abundant quantitative trait loci exist for DNA methylation and gene expression in human brain. PLoS Genet.

[62] Stranger BE, Nica AC, Forrest MS, Dimas A, Bird CP, Beazley C (2007). Population genomics of human gene expression. Nat Genet.

