# Supplementary material for: Integrated genome-wide analysis of expression quantitative trait loci aids interpretation of genomic association studies
Source: Genome Biol. 2017 Jan 25;18:16. doi: 10.1186/s13059-016-1142-6 (PMC5264466; doi:10.1186/s13059-016-1142-6)
Supplement: Supplementary file 1 — Supplementary Materials, including nine figures, Supplementary Methods, and 12 tables. (PDF 1844 kb) [file 13059_2016_1142_MOESM1_ESM.pdf]

Supplementary Materials

Integrated genome-wide analysis of expression Quantitative Trait Loci aids interpretation of genomic association studies

Roby Joehanes[1,2,3*], Xiaoling Zhang[1,10,12*], Tianxiao Huan[1], Chen Yao[1], Sai-xia Ying[2], Quang Tri Nguyen[2], Cumhur Yusuf Demirkale[2], Michael L. Feolo[4], Nataliya R. Sharopova[4], Anne Sturcke[4], Alejandro A. Schäffer[4], Nancy Heard-Costa[5], Han Chen[6,10], Po-ching Liu[7], Richard Wang[7], Kimberly A. Woodhouse[7], Kahraman Tanriverdi[8], Jane E. Freedman[8], Nalini Raghavachari[9], Josée Dupuis[1,10], Andrew D. Johnson[1], Christopher J. O'Donnell[1,11], Daniel Levy[1*], Peter J. Munson[2*]

[1]The Framingham Heart Study, Framingham, MA, USA and the Division of Intramural Research, National Heart, Lung and Blood Institute, National Institutes of Health, Bethesda, MD, USA
[2]Mathematical and Statistical Computing Laboratory, Center for Information Technology, National Institutes of Health, Bethesda, MD, USA
[3]Institute for Aging Research, Hebrew SeniorLife, Harvard Medical School, Boston, MA, USA
[4]National Center for Biotechnology Information, National Library of Medicine, National Institutes of Health, Bethesda, MD, USA
[5]Department of Neurology, Boston University School of Medicine, Boston, MA, USA
[6]School of Public Health, Harvard University, Boston, MA, USA
[7]DNA Sequencing and Genomics Core, National Institutes of Health, Bethesda, MD, USA
[8]Department of Medicine, University of Massachusetts Medical School, Worchester, MA, USA
[9]National Institute on Aging, National Institutes of Health, Bethesda, MD, USA
[10]Department of Biostatistics, School of Public Health, Boston University, Boston, MA, USA
[11]Cardiology Section, Department of Medicine, Boston VA Healthcare, Boston, MA, USA
[12]Section of Biomedical Genetics, Department of Medicine, Boston University School of Medicine, Boston, MA, USA
*Denotes equal contributions

Fig. S1. Quantile-quantile plot of p-values for eQTL analysis. Performed on 100,000 subsampled SNPs, with λ=0.9859 and median of observed p-value = 0.50303. A total of 1,787,300,000 p-values are plotted.

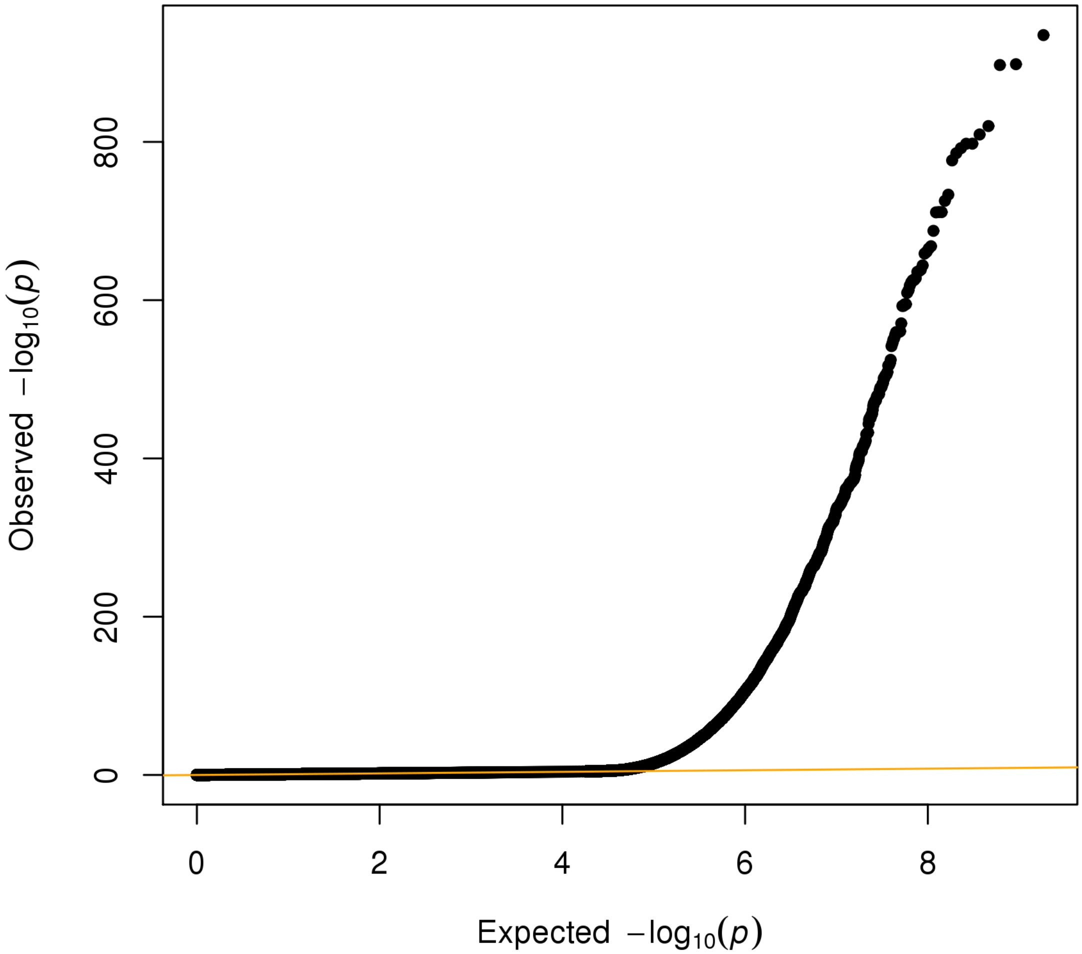

Fig. S2. Example of SNP-in-Probe problem for Transcript Cluster 2617687 (XYLB). A. Gene-level analysis showing lead variant (green) and three eQTLs which fall in certain probesets for this transcript cluster (black). One eQTL has R^2 value within 90% of highest R^2 for this transcript cluster (Green), indicating that this Transcript Cluster is candidate for SNP-in-probe problem. B. Exon-level analysis for 18 probesets within this transcript cluster. The SNPs falling into one of three probesets are indicated (black). One probeset, 2617715 includes eQTL (black) which is above 95% of best R^2 for all probesets, it is a likely cause of a SNP-in-probe problem.

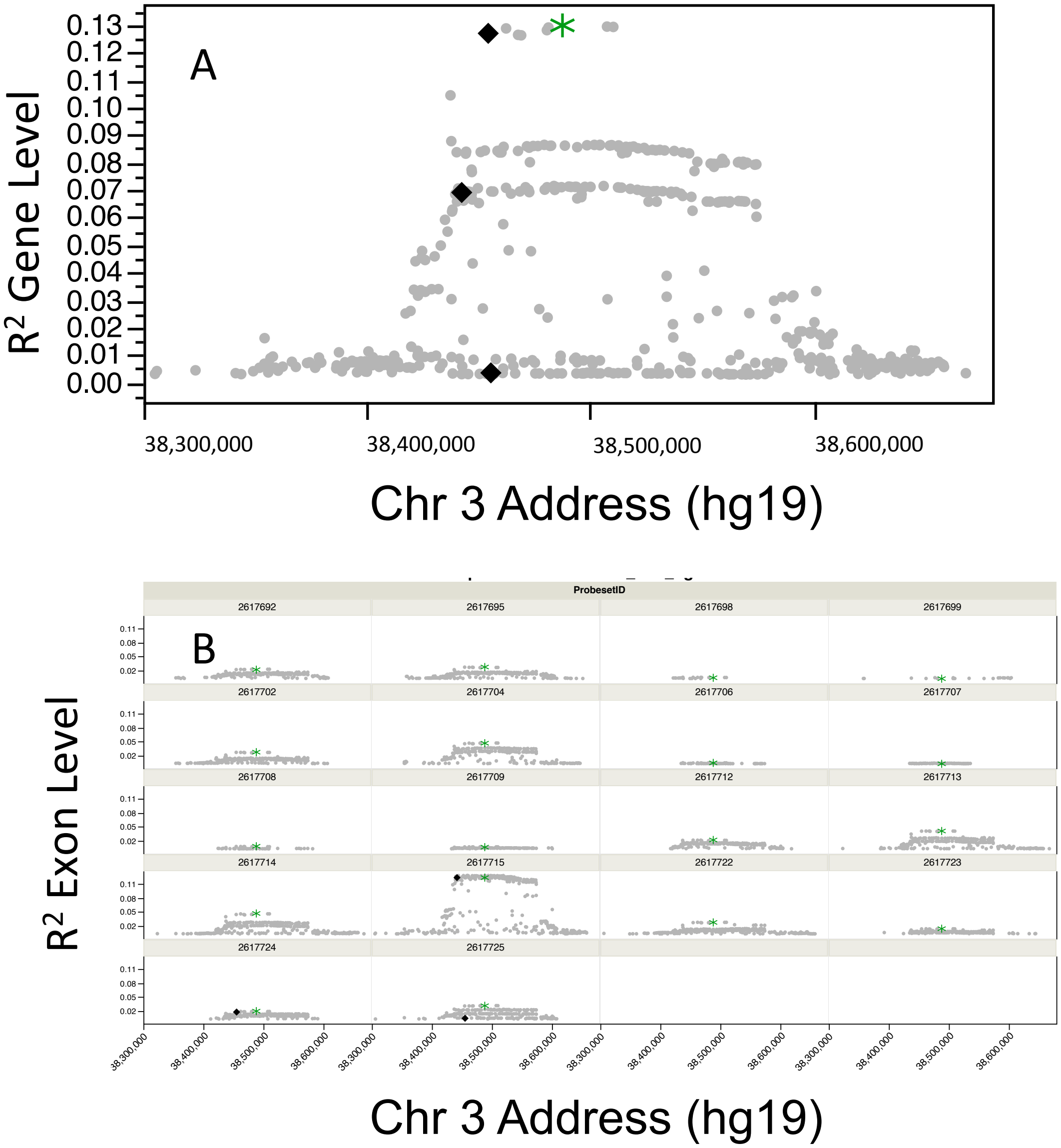

Fig. S3. Dot plot for exon-level expression. The bigger the bubble the smaller the FDR is. The smallest bubble indicates FDR<1e-16. The largest bubble indicates FDR<1e-300.

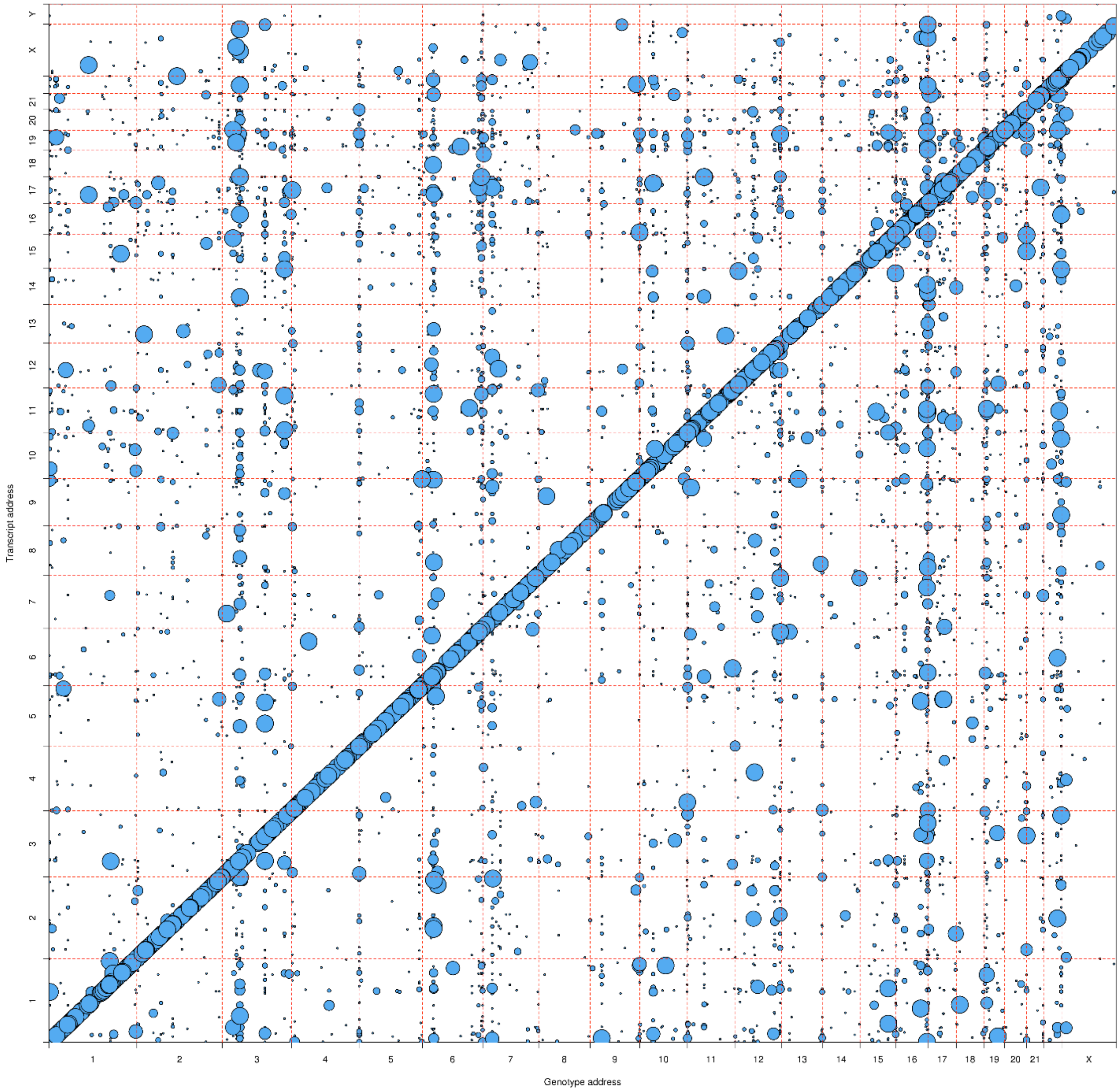

Fig. S4. Mean detection rate of cis-eGenes (represented by transcript cluster IDs, TCIDs) and mean detection rate of validated cis-eGenes vs Number of Probesets per TCID. The error bars show the 95% confidence interval of the mean. The mean detection rate (total, red or validated, blue) rises with number of probesets until about 21 to 25 probesets are available. Validation is counted in any of 5 external databases, for any SNP targeting this eGene. Bins indicate upper bound for number of probesets in bin. TCIDs with > 75 probesets included in last bin. A probeset consists generally of four 25 base probes on the Affy Exon array. A transcript cluster consists of from 1 to several hundred probesets.

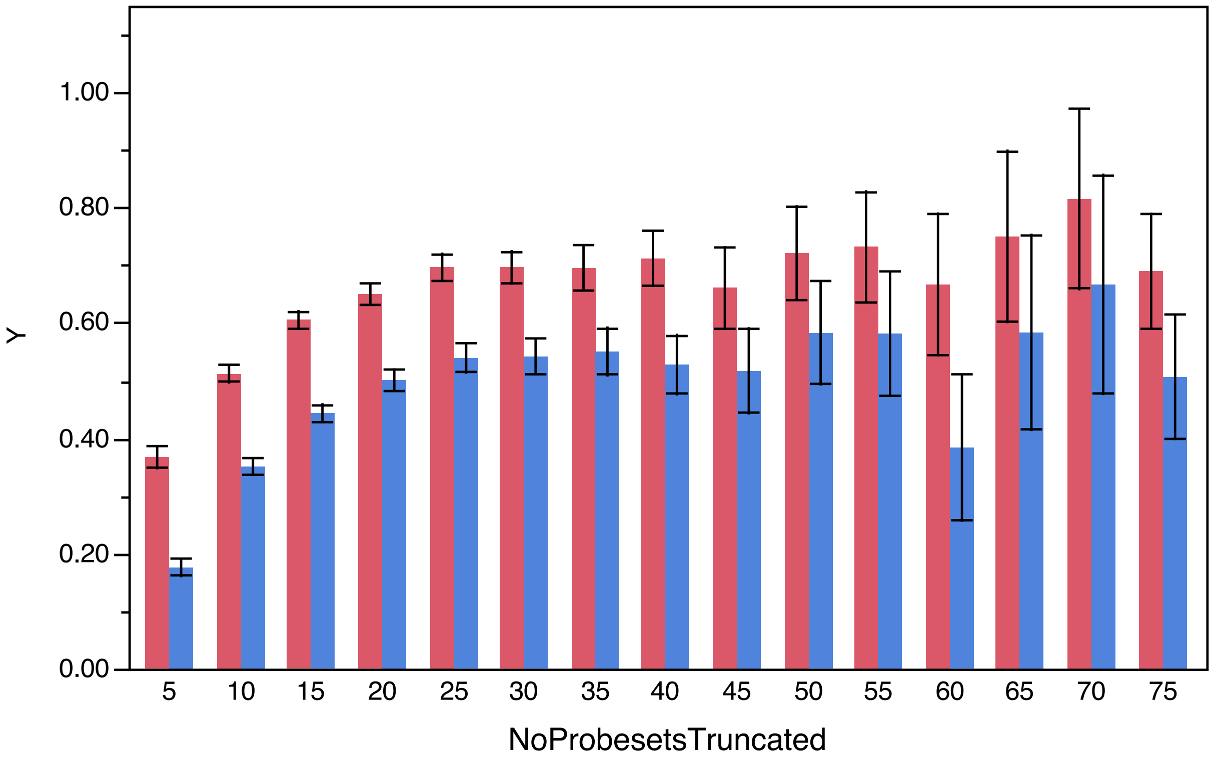

Fig. S5. Distribution of Primary Lead cis-eQTLs Relative to Transcription Start Site (TSS) and Transcription End Site (TES), by Transcript Length (A. Large, >=58 Kb; B. Medium, >=18 Kb and < 58 Kb; C. Small, < 18 Kb). Location of eQTL has been interpolated within [TSS, TES] interval, and weights suitably adjusted. Lead eQTLs exclude those with polymorphism-in-probe issues. 8,475 Transcript clusters are represented, having RefSeq annotations for TSS, TES.

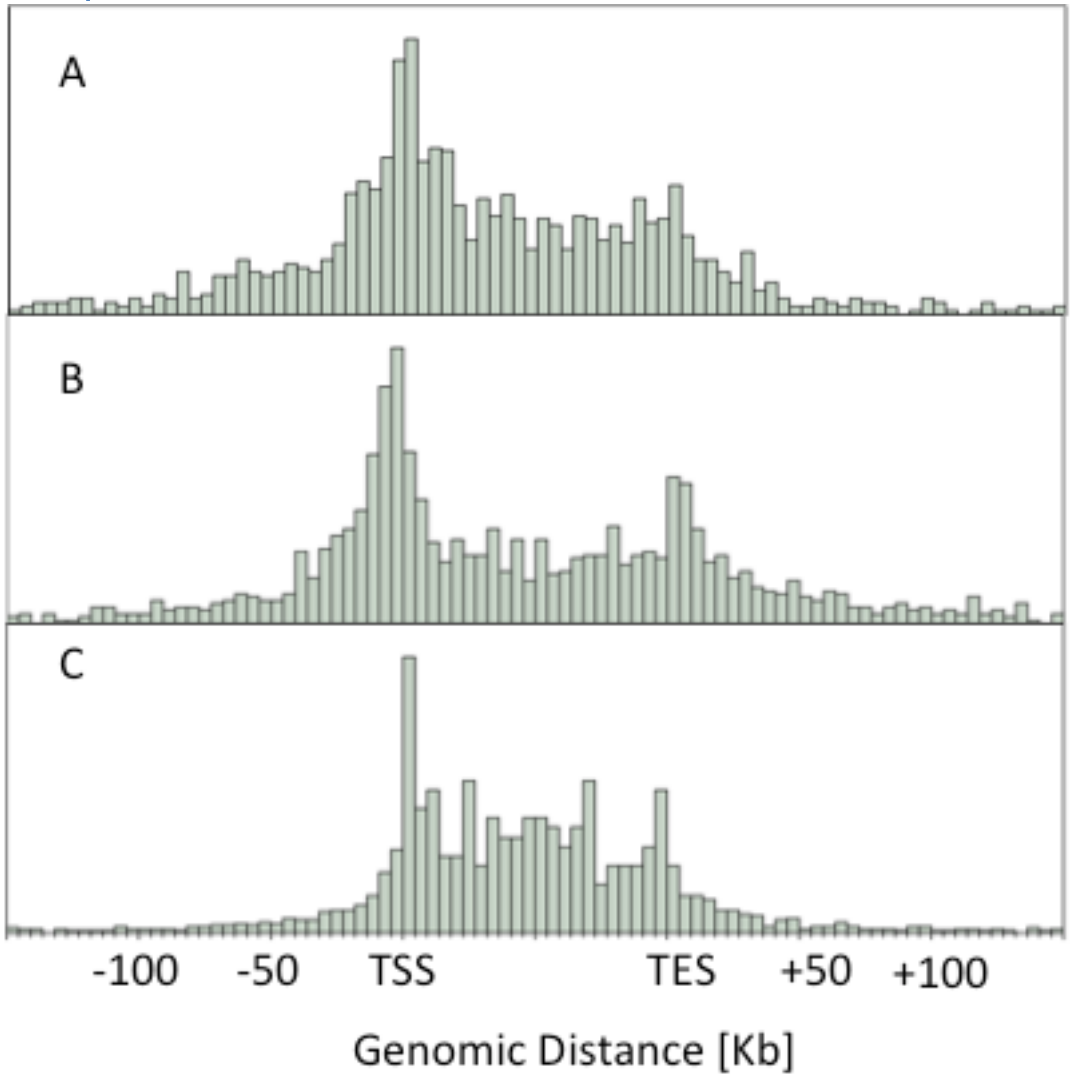

Fig. S6. Number of targets and validated targets of extrachromosomal trans-eQTLs by chromosome. A. Colors indicate trans-eQTLs with 6 or more targets, grouped into clusters and numbered as in Table 4. B. Number of validated targets for same eQTLs. Clusters 4, 6, 10, 11, 17, 18, 25, 29, 33, 34, 35, 38, 39, and 51 include one or more trans-eQTLs with validated targets. Clusters 6, 10 and 39 have validated targets for the strongest "lead variant" trans-eQTL.

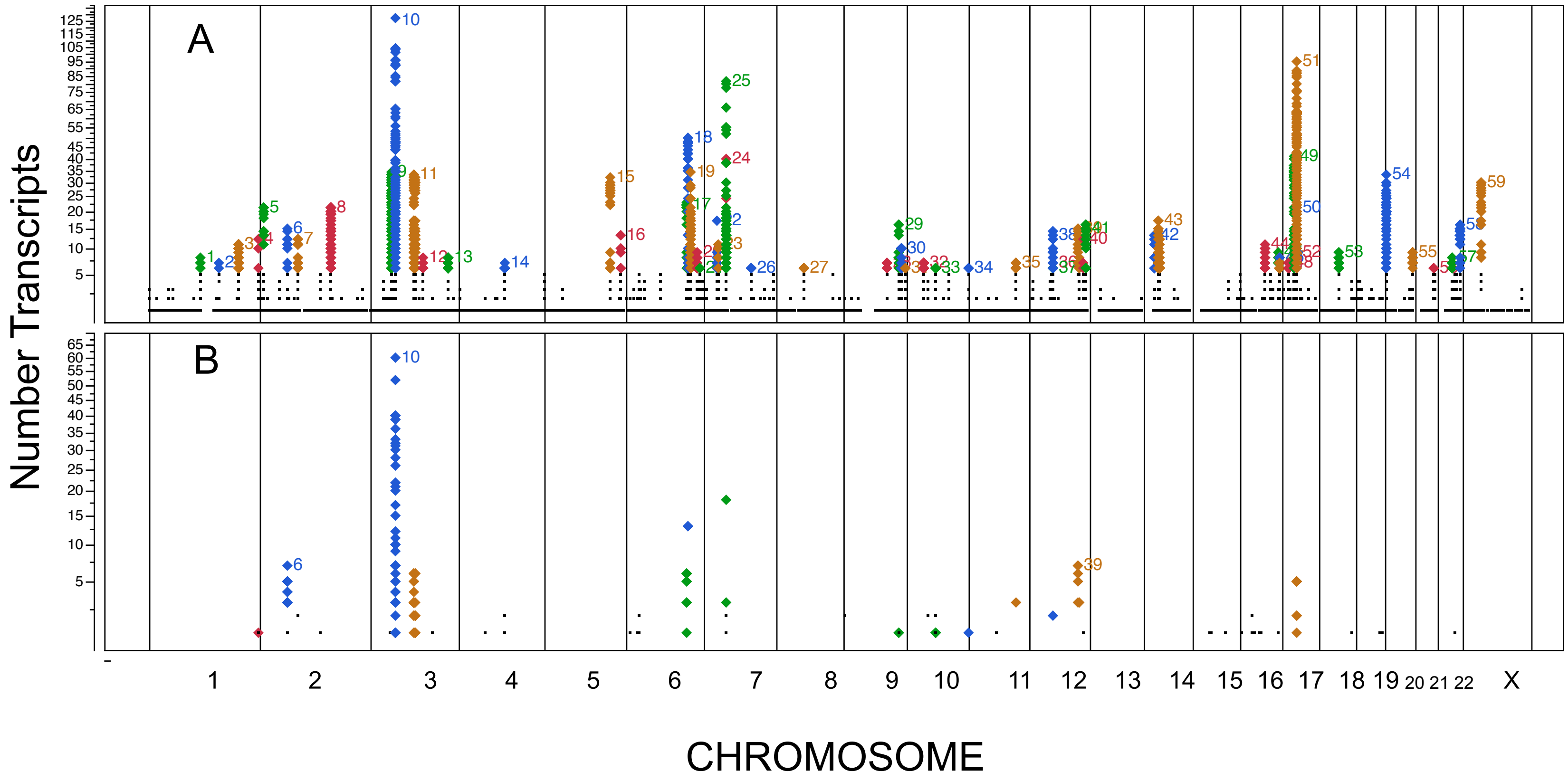

Fig. S7. Principal component analysis of Framingham genetic data. We performed a principal component (PC)[1] analysis with 521 unrelated FHS participants (Green) along with HapMap individuals (CEPH with Northern and Western European ancestry (CEU, Pink), Yoruba from Ibadan, Nigeria (YRI, Red), Han Chinese from Beijing (CHB, Blue) and Japanese from Tokyo (JPT, Blue). The samples which entered the eQTL study are shown in dark Green. Additional FHS participants were projected onto the first two PCs derived from that analysis (See Methods for details.)

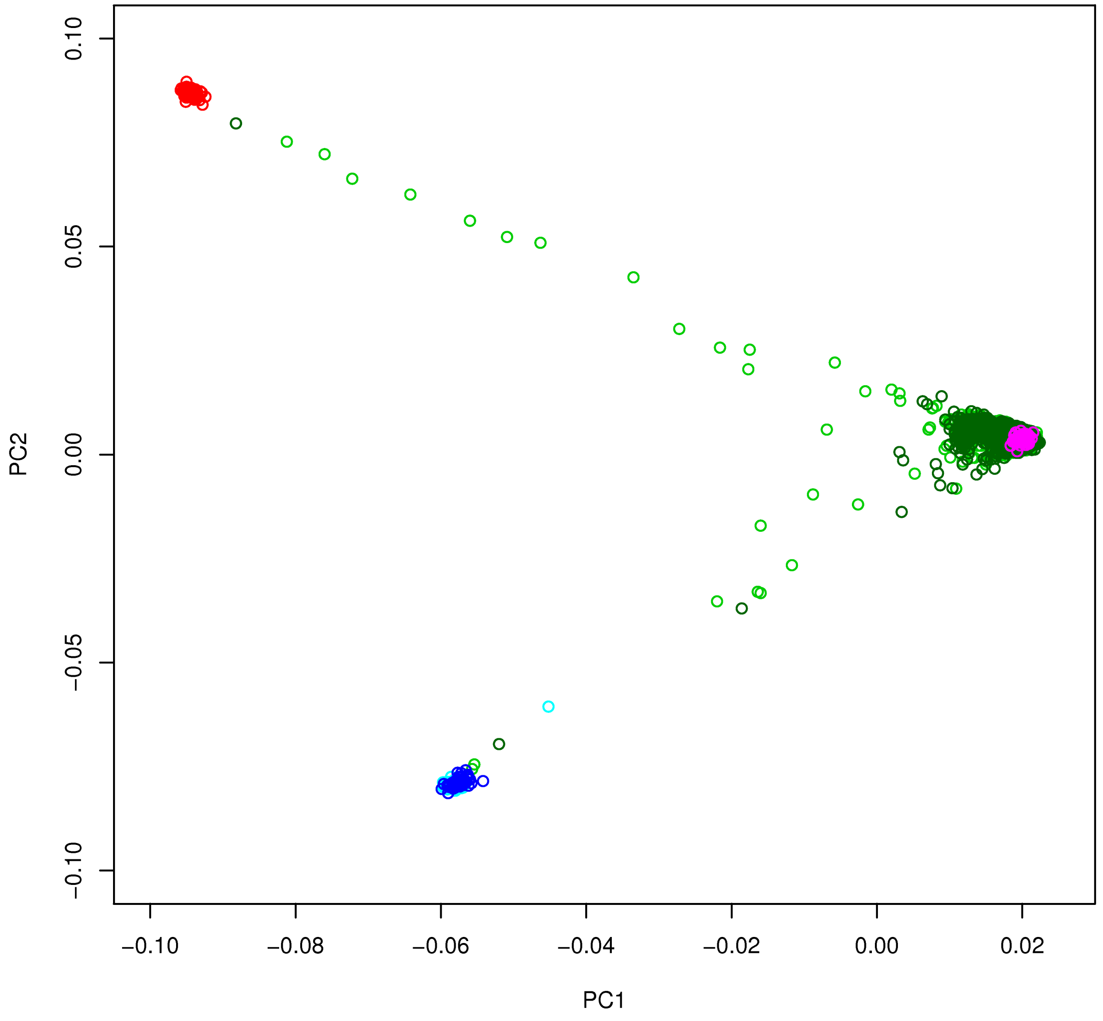

Fig. S8. Effect of Imputation RSq on Validation Rate. For eligible, HapMap cis-eQTLs, validation rate is the maximum of validation for any Transcript Cluster ID, for the Multiple Studies. Average validation rate is 0.69 is shown. 95% Confidence Intervals are shown.

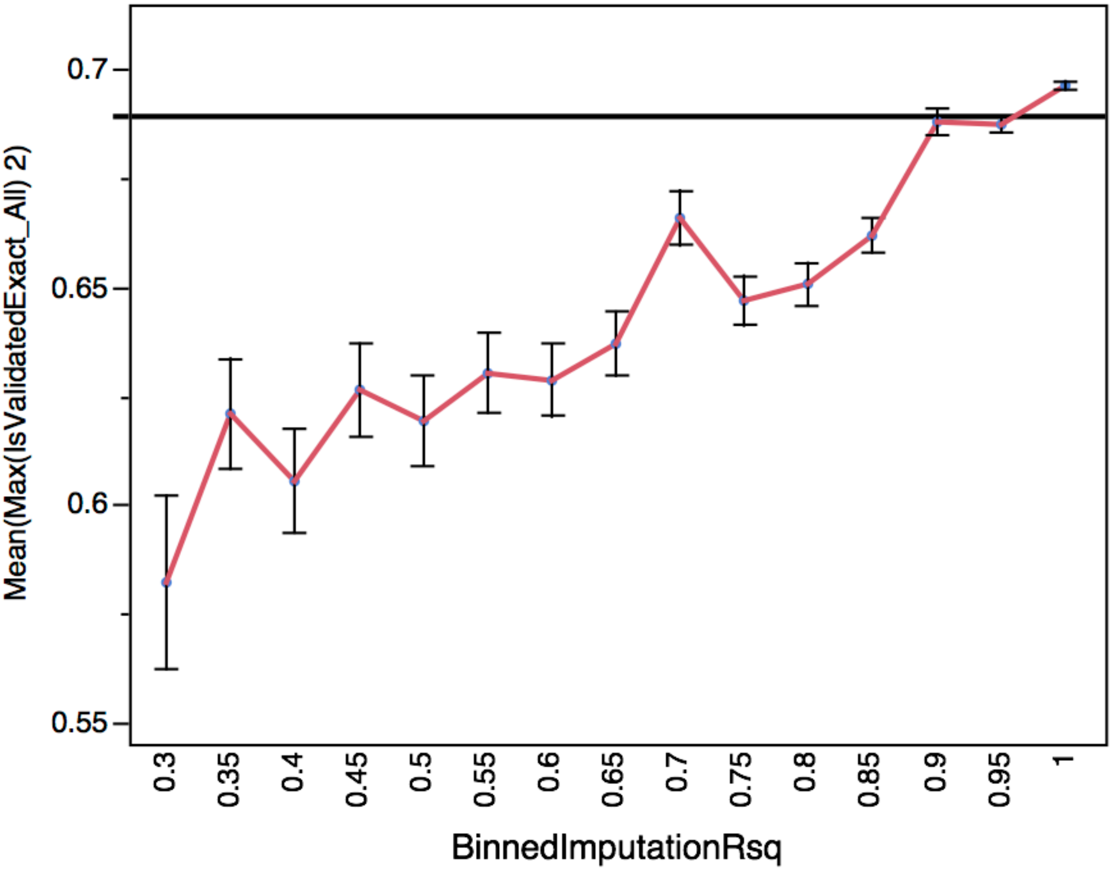

Fig. S9. Mean validation rate of cis-eGenes by Mean Expression Level. Mean expression level (RMA units) was binned. Relative validation rates, along with 95% confidence limits are shown. Validation rates (measured as number of eGenes validated in any of 5 studies for any cis-eQTL, divided by number of detected eGenes) is above the weighted average ratio when the expression level is above 5.

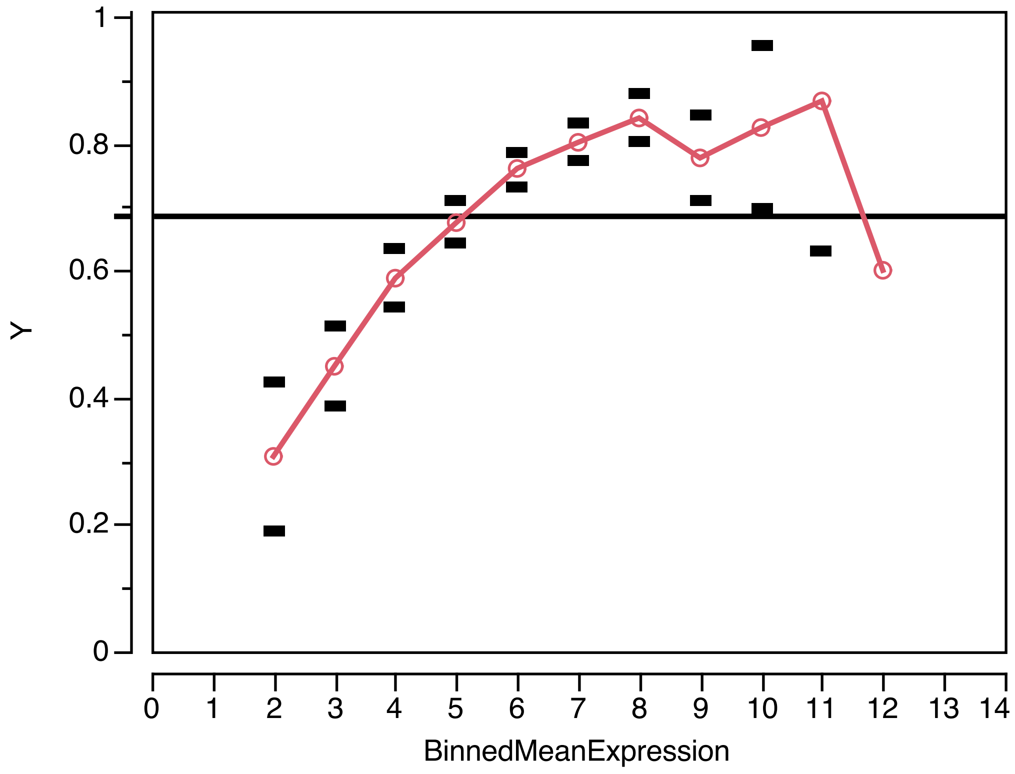

Supplementary methods

Polymorphism-in-probe

Sequence polymorphisms might conceivably affects the hybridization efficiency on the Affymetrix chip when the polymorphism appears within the designed probe sequence. If this situation is the main reason for the apparent significance of an eQTL-gene pair, we would expect that the significance would be close to maximal at the SNP which fell within the probe. Because of linkage-disequilibrium, nearby SNPs might also appear significant but to a lesser degree. Since we have both gene-level and exon-level results for each gene, we would also expect the polymorphism-in-probe signal would be strongest in the particular probeset containing that SNP and weaker or no-existent in other probesets. We constructed a rule to implement this expectation. Specifically, we downloaded the locations of Affymetrix core probes from UCSC Golden Path (http://hgdownload.cse.ucsc.edu/goldenPath/hg19/database/) in the file *affyExonProbeCore.txt.gz*. This file contained the hg19 address for 1,081,123 Probes comprising 283,927 Probesets and 18,693 Transcript Clusters. This file was joined with our file of 8,510,936 eligible SNPs (MAF>=0.01, Imputation quality R2>=0.3) to yield a list of 44,015 Probes, (along with 27,543 corresponding Probesets and 12,032 corresponding Transcript Clusters) which contained one or more eligible SNPs, i.e. the candidates for a polymorphism in problems. Next, we summarized our gene-level results to compute for each gene, the ratio of the maximal R2 over the SNPs in a probe to that over every eligible SNP. We also summarized the corresponding exon-level results for each gene to obtain the ratio of R^2 to the maximum R^2 for that gene. If 1) the first, gene-level ratio was greater than 0.9 and 2) the second, exon-level ratio was greater than 0.95, we determined that the results for that transcript cluster were probably or likely due to the polymorphism-in-probe issue. The number of Transcript Clusters satisfying the first criterion was 1,822. The number which also satisfied the second criterion was 988 or 9.6% of the 10,327 detected eGenes. See illustrative example in Fig. S2.

There is a possibility that a cryptic polymorphism, not included in our set of 8.5 million, fell inside a probe, and had strong LD with a measured SNP outside the probe and thereby caused a false positive eQTL. This should be rare, since if a well-imputed (R2>0.3) polymorphism outside a probe is in strong LD with a cryptic polymorphism in probe, that polymorphism itself should also be well-imputed, and thus already accounted for in our procedure.

Differential blood count imputation

Of the 5,257 samples, 2,181, from the third generation cohort had whole blood complete blood cell counts (CBCs, Beckman Coulter, Brea, California). The cell counts of the remaining samples were imputed using a Partial Least Square (PLS) prediction based on the gene expression data. We trained our model on two thirds of the 2,181 samples with CBC measurements, while reserving the other third as testing data. We used the expression values of all 17,873 genes in log 2 scale after normalized with the robust multi-chip average (RMA) method available in the Affymetrix Power Tools (APT)[2] Software version 1.12.0. The

training was performed in 10-fold validation fashion on the training dataset only, using the plsr function of pls package[3] in R version 3.0.1, to predict 8 CBC components: red blood cell count (RBC), white blood cell count (WBC), platelet count, and percentages of neutrophil, lymphocyte, monocyte, eosinophil, and basophil. We selected the model giving the highest cross-validated R^2 on the training dataset. The model was then applied to the test dataset, giving the following testing R^2: 0.61, 0.41, 0.25, 0.83, 0.83, 0.81, 0.89, and 0.25, for RBC, WBC, platelet count, and percentages of neutrophil, lymphocyte, monocyte, eosinophil, and basophil, respectively. We then applied the selected model to the remaining samples that do not have CBC measurements. We conducted comparisons between results of using imputed cell counts and that of using measured ones and did not find substantial difference. Thus, we used measured cell counts when available and imputed ones when not.

Validation analysis details

For Multiple Studies[4–8], we performed probeset address overlap analysis between Affymetrix HuEx and the other platforms to ensure that the probes or transcripts are measuring some common part of the gene. We did not perform gene symbol-based matching because two transciptome platforms may measure different parts of the gene. Rather, to ensure the accuracy of the transcript annotation, we downloaded manufacturer's latest annotation files: for Affymetrix Human Exon ST 1.0 (HuEx), we used HuEx-1_0-st-v2-na33.1.hg19.probeset.csv and HuEx-1_0-st-v2-na33.1.hg19.transcript.csv; for Affymetrix Human Genome U133 Plus 2, we used HG-U133_Plus_2.na33.annot.csv; for Illumina HumanHT12, we used HumanHT-12_V3_0_R3_11283641_a.zip (for v3) and HumanHT-12_V4_0_R2_15002873_b.zip (for v4). Since Illumina annotation files contain only hg18 coordinates, we performed translation to hg19 using the command line liftOver tool. Probes with addresses that did not translate were discarded. Affymetrix annotation files are already in hg19 coordinates. The annotation columns of published datasets using older coordinate system were then replaced with the new ones, unless when there is no information on the newer annotation files. In the latter case, we assumed that the authors of the published datasets obtained additional annotations elsewhere. We included only probesets that were measured in at least one of the external databases. We also required the SNP RS ID or the SNP genomic address to match exactly and that the probes of the transcript overlap. To ensure the accuracy of matching SNPs, we used dbSNP version 137 and updated all the SNP addresses accordingly (in hg19 coordinates). For SNPs that did not have an rs ID number in dbSNP, but only an hg18 addresses, we performed liftOver translation to hg19 coordinates. We also did not consider linkage disequilibrium for proxy SNPs. Because only one of the external dataset used imputation to the 1000G SNP set while the rest used HapMap SNPs, we limited the validation efforts to HapMap version 3 SNPs only.

For Battle et al[7] , we found eligible SNPs those appearing on both the Illumina HumanOmni1-Quad_v1 BeadChip (which interrogated 1,124,584 SNPs) and the list of 8,502,797 SNPs analyzed by FHS, resulting in 800,134 SNPs. We did not have access to SNP quality data, so no doubt this number is an over-estimate. Since Battle, et al[7] used RNA-Seq instead of microarray for determining expression, we determined the eligible transcripts

by finding the common transcripts defined by both the NCBI v37.2 *H. sapiens* reference genome (25,962 transcripts) and the Affymetrix na33.1 annotations of the HuEx-1_0-st chip (17,471 transcripts). We found 15,596 transcripts to be eligible for validation.

References

1. Price AL, Butler J, Patterson N, Capelli C, Pascali VL, Scarnicci F, et al. Discerning the ancestry of European Americans in genetic association studies. *PLoS Genet*. 2008;4:e236.

2. Affymetrix. Affymetrix Power Tools [Internet]. Affymetrix; Available from: http://www.affymetrix.com/partners_programs/programs/developer/tools/powertools.affx

3. Mevik B, Wehrens R. The pls package: principal component and partial least squares regression in R. *J Stat Soft*. 2007;18:1–24.

4. ENCODE Project Consortium. An integrated encyclopedia of DNA elements in the human genome. *Nature*. 2012;489:57–74.

5. Westra H-J, Peters MJ, Esko T, Yaghootkar H, Schurmann C, Kettunen J, et al. Systematic identification of trans eQTLs as putative drivers of known disease associations. *Nat Genet*. 2013;45:1238–1243.

6. Liang L, Morar N, Dixon AL, Lathrop GM, Abecasis GR, Moffatt MF, et al. A cross-platform analysis of 14,177 expression quantitative trait loci derived from lymphoblastoid cell lines. *Genome Research*. 2013;23:716–726.

7. Battle A, Montgomery SB. Determining causality and consequence of expression quantitative trait loci. *Hum Genet*. 2014;133:727–735.

8. Kirsten H, Al-Hasani H, Holdt L, Gross A, Beutner F, Krohn K, et al. Dissecting the genetics of the human transcriptome identifies novel trait-related trans-eQTLs and corroborates the regulatory relevance of non-protein coding loci†. *Hum Mol Genet*. 2015;24:4746–4763.

9. Stegle O, Parts L, Durbin R, Winn J. A bayesian framework to account for complex non-genetic factors in gene expression levels greatly increases power in eQTL studies. *PLoS Computational Biology*. 2010;6:e1000770.

10. Leek JT, Storey JD. Capturing heterogeneity in gene expression studies by surrogate variable analysis. *PLoS Genetics*. 2007;3:e161.

11. Nikpay M, Goel A, Won H-H, Hall LM, Willenborg C, Kanoni S, et al. A comprehensive 1,000 Genomes-based genome-wide association meta-analysis of coronary artery disease. *Nat Genet*. 2015;47:1121–1130.

12. Schunkert H, König IR, Kathiresan S, Reilly MP, Assimes TL, Holm H, et al. Large-scale association analysis identifies 13 new susceptibility loci for coronary artery disease. *Nat Genet*. 2011;43:333–338.

13. CARDIoGRAMplusC4D Consortium, Deloukas P, Kanoni S, Willenborg C, Farrall M, Assimes TL, et al. Large-scale association analysis identifies new risk loci for coronary artery disease. *Nat Genet*. 2013;45:25–33.

14. Huan T, Esko T, Peters MJ, Pilling LC, Schramm K, Schurmann C, et al. A meta-analysis of gene expression signatures of blood pressure and hypertension. *PLoS Genet*. 2015;11:e1005035.

Table S1. Effect of reducing sample size on detection rates of eQTL-Transcript Pairs, Independent eQTLs or eGenes.

Cohort	Cohort Size		SNP:Transcript Pairs		Independent Lead eQTLs		Unique eGenes		Lead eQTLs per eGene
			Cis						
Full	5,257	100%	4,285,456	100%	19,239	100%	10,327	100%	1.86
Offspring	2,240	43%	1,846,181	42%	10,623	55%	6,289	61%	1.69
Gen3	3,017	57%	2,162,746	49%	11,904	62%	6,850	66%	1.74
			Trans						
Full	5,257	100%	216,169	100%	5,749	100%	4,958	100%	1.15
Off	2,240	43%	78.128	46%	813	14%	854	17%	0.95
Gen3	3,017	57%	115,289	63%	1,482	26%	1,531	31%	0.97

Table S2. Number of eQTL-Gene or eQTL-Exon pairs and Internal Validation rates

	Cis/Trans**	Technical covariate adjustment only	20 PEER factors[9] on unadjusted data	20 PEER factors on adjusted data*	40 Principal Components on unadjusted data	40 Surrogate Variables[10] on unadjusted data
			Gene-Level			
Offspring	Cis	1,352,644	1,675,618	1,729,786	1,795,863	1,463,999
	Trans	275,434	435,880	341,450	462,351	512,512
Generation 3	Cis	1,606,135	2,087,220	2,010,813	2,207,789	1,892,934
	Trans	301,154	667,565	381,382	658,834	618,443
Overlap	Cis	1,172,959	1,478,934	1,503,063	1,580,763	1,293,493
	Trans	126,724	174,566	156,874	157,002	148,860
Validation rate	Cis	73.03%	70.86%	74.75%	71.60%	68.33%
	Trans	42.08%	26.15%	41.13%	23.83%	24.07%
			Exon-Level			
	Cis	-	-	19,843,026	-	-
Offspring	Trans	-	-	2,671,798	-	-
	Cis	-	-	22,503,147	-	-
Generation 3	Trans	-	-	3,325,594	-	-
	Cis	-	-	15,789,096	-	-
Overlap	Trans	-	-	2,040,249	-	-
	Cis	-	-	70.16%	-	-
Validation rate	Trans	-	-	61.34%	-	-

*Chosen method, based on Gene-level results and also used for Exon-level results. Validation Rate is Overlap divided by Generation 3.

**Method of defining cis/trans used here differs from that used in rest of paper. Here, cis-eQTL means transcript start as defined genomically in Affymetrix annotation is within 1 Mb of eQTL.

Table S3. Top 25 Exon-level lead cis and top 25 Exon-level lead trans Probeset-eQTL pairs.

Marker Position	Rs_ID	Transcript Cluster ID	Probeset ID	Trans Chr	Gene Symbol	R^2	Beta	Cluster Number
		Top Cis-eQTL pairs						
12:10118747	rs12231872	3404530	3404536	12	*CLEC12A*	56%	-0.22[G]	
X:24086687	rs7882734	3971877	3971879	X	*EIF2S3*	54%	0.15[G]	
6:32576341	rs9271093	4048241	4048242	6	*HLA-DRB5*	53%	-0.39[A]	
15:48596713	rs74011998	3593065	3593088	15	*SLC12A1*	53%	-0.37[T]	
6:32576341	rs9271093	4048265	4048279	6	*HLA-DRB1*	52%	-1.02[A]	
20:1552430	rs2253427	3894727	3894744	20	*SIRPB1*	51%	-0.61[C]	
12:56435929	rs1131017	3417240	3417244	12		51%	-0.20[G]	
5:171533782	rs2306961	2887048	2887089	5	*STK10*	50%	0.22[T]	
17:44793503	rs199439	3760268	3760285	17	*ARL17A*	49%	-0.33[A]	
1:207280764	rs12063500	2377165	2377174	1	*C4BPA*	49%	-0.78[C]	
5:118811533	rs25640	2825733	2825744	5	*HSD17B4*	48%	-0.14[G]	
1:155209360	rs2075569	2437205	2437227	1	*GBAP1*	47%	-0.20[T]	
19:36230174	rs12459634	3859899	3859901	19	*IGFLR1*	47%	0.24[T]	
17:45438886:CAGTG		3724591	3724617	17	*C17orf57*	47%	0.34[R]	
7:150476888	rs7806458	3079172	3079179	7	*TMEM176B*	47%	-0.26[A]	
8:100896323	rs7812991	3146433	3146443	8	*COX6C*	47%	-0.20[G]	
X:109206541	rs2499412	3987029	3987102	X	*TMEM164*	47%	0.20[G]	
4:6697822	rs3822260	2717078	2717088	4	*S100P*	46%	0.29[C]	
7:150442551	rs10271868	3031573	3031599	7	*GIMAP1-GIMAP5*	46%	0.25[T]	
9:132588337	rs7470675	3227121	3227132	9	*C9orf78*	46%	0.18[T]	
5:96252589	rs2910686	2821347	2821382	5	*ERAP2*	45%	-0.24[T]	
4:10099030	rs3822237	2760371	2760413	4	*WDR1*	45%	-0.17[A]	
22:42498204	rs12157818	3947310	3947316	22	*C22orf32*	45%	0.24[C]	
6:26392515	rs2072803	2899333	2899339	6	*BTN3A2*	45%	0.35[G]	
5:180375312	rs2921513	2844888	2844903	5	*BTNL3*	45%	-0.60[T]	

Marker Position	Rs_ID	Transcript Cluster ID	Probeset ID	Trans Chr	Gene Symbol	R^2	Beta	Cluster Number
		Top Trans-eQTL pairs						
12:56435929	rs1131017	2782504	2782509	4		51%	-0.17[G]	
20:62551785:GC_G		3610982	3610999	15	*SYNM*	47%	-0.19[R]	55
16:69974448	rs3748388	2876543	2876551	5	*TIFAB*	47%	-0.35[A]	
3:176912899	rs12493005	3554360	3554361	14	*ADSSL1*	47%	0.19[C]	13
22:39131727	rs1043312	2572909	2572930	2	*EN1*	45%	-0.34[T]	
20:62551785:GC_G		3623751	3623769	15	*USP50*	45%	-0.27[R]	55
17:44793503	rs199439	2878461	2878467	5	*WDR55*	45%	-0.16[A]	
3:39456928	rs13082342	4014387	4014399	X	*RPSA*	45%	0.12[T]	
3:50039474	rs7628058	3939154	3939164	22	*RAB36*	45%	0.43[G]	9
3:39456928	rs13082342	3827218	3827234	19	*RPSAP58*	45%	0.12[T]	
15:31157666	rs12439909	3614901	3614938	15	*HERC2*	43%	-0.21[A]	
3:176912899	rs12493005	3391029	3391052	11	*PPP2R1B*	43%	0.15[C]	13
3:50039474	rs7628058	3981931	3981932	X	*ZCCHC13*	43%	0.42[G]	9
3:50037940	rs6767381	4024310	4024313	X	*SOX3*	42%	0.20[A]	9
1:182350107	rs10429835	2376168	2376195	1	*NFASC*	42%	0.33[A]	
8:56977485	rs2953922	3103494	3103514	8	*TMEM70*	42%	0.27[G]	
16:87764267	rs9934565	2391425	2391451	1	*DVL1*	41%	-0.22[A]	45
10:43310305	rs11239784	3256221	3256230	10	*AGAP11*	41%	0.22[G]	
16:87764267	rs9934565	3257031	3257071	10	*STAMBPL1*	40%	-0.21[A]	45
3:50037123	rs3774733	3693591	3693605	16	*PRSS54*	40%	0.22[C]	9
2:114453472: A_AT	rs145131572	3951136	3951147	22	*RPL23AP82*	39%	-0.25[R]	
22:50036897	rs5770610	3948590	3948607	22	*RIBC2*	39%	0.38[A]	58
22:40603580	rs6001837	3873389	3873392	20	*PSMF1*	39%	-0.41[A]	
5:179036268	rs60104959	3194470	3194557	9	*EGFL7*	38%	-0.27[A]	
15:80126245	rs4779162	2440117	2440129	1	*PEX19*	38%	0.28[T]	

R^2 - Percentage Variance Explained
Marker position is annotated as chromosome number:location in hg19 coordinate
Beta is regression estimate (log base 2 expression difference per dosage of minor allele), with minor allele in brackets. [R] refers to the reference allele for an indel polymorphism

Table S4. Enrichment of Primary or Secondary Lead cis-eQTLs by Functional Region.

Functional Region	Enrich-ment	Obs.	Exp.	Log10 Pvalue	Enrichment	Obs.	Exp.	Log10 Pvalue
	Primary Lead eQTL				Secondary Lead eQTL			
Intergenic Up	**0.8**	3198	4235.0	-113	**0.8**	3092	3799.3	-59
Transcribed Region	**9.4**	2951	312.3	<-280	**7.2**	2274	314.2	<-280
Intergenic Down	**0.6**	2339	3940.7	-279	**0.6**	2253	3505.5	-190
5'- UTR	**45.0**	108	2.4	-140	**29.7**	65	2.2	-73
All Exons	**25.4**	406	16.0	<-280	**17.7**	266	15.0	-235
First Exon	**44.6**	138	3.1	-178	**26.4**	72	2.7	-77
Other Exons	**20.8**	268	12.9	-251	**15.8**	194	12.3	-161
All Introns	**8.6**	2545	296.3	<-280	**6.7**	2008	299.2	<-280
First Intron	**10.9**	822	75.6	<-280	**8.3**	621	74.8	<-280
Other Introns	**7.8**	1723	220.7	<-280	**6.2**	1387	224.4	<-280
3'- UTR	**19.7**	151	7.7	-138	**13.0**	98	7.6	-73

Enrichment = Observed/Expected frequency. Primary Lead eQTL analysis based on 8,475 transcripts (TCIDs). Secondary Lead eQTL analysis based on 4,188 TCIDs with one or more secondary lead eQTLs. All transcripts with potential Polymorphism-in-Probe issues were excluded. For each TCID, we consider a pruned subset of SNPs having low mutual LD (see Methods) falling in 2 Mb region centered on transcription start site. Each SNP is either in or not in the Functional Region, and is or is not a Lead eQTL, forming a 2 by 2 table, which is then summarized over all TCIDs. P-values are determined using Fisher's exact test.

Table S5. Enrichment of lead eQTLs with strong evidence as regulatory positions.

RegulomeDB Evidence Scores	Primary and Secondary Lead eQTLs (expected)	Primary Lead eQTL only (expected)	SNPs with RegulomeDB Scores	Non-Significant SNPs
		Cis-eQTLs		
Strong (1a & 2a)	121(18)	70(9)	14,236	8,344
None (6 & 7)	8,035	4,118	4,903,615	3,766,681
Enrichment	6.7	7.7		
Log10 pvalue	-89.59	-69.47		
		Trans-eQTLs		
Strong (1a & 2a)	17(8)	16(8)	14,417	14,170
None (6 & 7)	3,077	2,968	5,336,743	5,294,488
Enrichment	2.1	2.0		
Log10 pvalue	-2.3	-2.1		

- RegulomeDB Scores 1a&2a = TF binding & matched TF motif & matched DNase Footprint & DNase peak
- Enrichment (observed/expected) compares All and Primary Lead eQTLs with non-Significant eQTLs
- Log10 of Pearson Chi-Square p-value, with adjustment for LD in non-Significant SNPs
- All lead eQTLs targeting genes with potential polymorphism-in-probe have been excluded here.

Table S6. Platelet-related clusters

Cluster No.	Top GSEA FDR*	Contains platelet GWAS SNPs
1	3.4e-8	No
3	N/S	Yes
4	N/S	Yes
10	7.0e-45	Yes
17	N/S	Yes
26	N/S	Yes
33	0.0007	Yes
34	0.0002	Yes
37	N/S	Yes
38	1.1e-7	Yes
39	N/S	Yes
40	9.7e-17	Yes
51	3.9e-14	Yes
52	1.7e-6	Yes

*There are multiple platelet-related categories in the GSEA results. The number in this column reflects the false discovery rate (FDR) for the top category.

Table S7. Genes targeted by one or more eQTLs in each gene-level cluster.

Cluster	Targeted Genes
1	*C6orf25, CCL5, CLU, KIAA0430, NGFRAP1, PF4, PF4V1, PPBP, PROS1, TIMP1, TREML1*
2	*DNAJC3, HECTD1, IGF1R, IQGAP2, SIGLEC10, ST8SIA4, TECPR2*
3	*ACP1, AMIGO3, C7orf41, EMC3, GMPPB, GYPA, GYPB, MAP2K3, OPA1, PGM2L1, PIM1, RNF123, STOM, TCP11L2, TFDP1, TFDP3, UBAP1, UBE2H*
4	*BEND2, C15orf54, CPEB4, DPM2, GABARAPL2, GCLC, GPR55, HIST1H2AB, HIST1H2AE, JAM3, LOC283788, MAP2K3, OPTN, PLEK2, SLC1A5, ZER1, ZNF542, ZSCAN5A*
5	*ALDH6A1, APOO, ARG2, C14orf45, COX6CP1, CREG1, CSDE1, DCK, ENTPD5, ERCC2, GRINA, GSPT1, GSPT2, ITLN1, KLC3, MCOLN1, MOB1B, PGM2L1, PLEK2, PSMF1, R3HDM4, RNF11, ROGDI, SGIP1, SHARPIN, SLC38A5, TMEM74B, TMOD1, TSTD2, TTC25, UBE2B, VTI1B, VWCE*
6	*C14orf57, C17orf39, DNAJA4, ESPN, ESPNP, LMOD1, MAP2K3, MKRN1, NINJ2, OAZ1, OR51A7, PGM2L1, PHOSPHO1, PPP2R5B, PTMS, RGS6, SHISA4, TNFRSF25, TPM1, VWCE, WNK1*
7	*CEACAM1, CRISPLD2, DPEP3, EXTL3, GNS, HERC3, MAPK14, MME, MRVI1, NAP1L5, NLRP1, PKN2, PRKDC, SDC2, SLC26A8, SNX27, SRPK1*
8	*ACPP, C16orf57, CASS4, CD93, DICER1, DPEP2, EXTL3, FRY, G2E3, HSPA1A, HSPA1B, IMPA2, KARSP3, KIAA0319L, KIAA0754, LOC100505519, LOC100507246, LOC100507570, MACF1, MAN2A2, MANSC1, MGAM, MGRN1, MSL3, NCOA3, NFIL3, NT5C2, RPN1, SIPA1L2, ST6GALNAC2, TIAM2, TRIM22, ZFYVE16, ZMYM4*
9	*ACTR1A, ADAM15, ATP4A, BPIFA3, BTNL2, C12orf61, CCDC113, DCST1, DEPDC1B, DHDH, EGLN1, EIF3E, ENPP7, EXOC8, FAM73A, FOXK2, GABRB3, GDEP, HTR4, IER5, KRT3, KRT76, LIMK1, LOC100505666, MT1F, MT1H, NAP1L5, NPPB, NSRP1, NSRP1P1, PDPN, PRSS54, RAB36, RCAN3, SLC16A4, SLC27A5, SLC9C2, SNX8, SOX3, SPRTN, SRPK2, SRSF6, ST18, TRIM16, TRIM16L, WDR45L, YIF1A, ZCCHC13, ZNF446, ZNF624, ZNF77*
10	*ABCC3, ABLIM3, AQP10, ARHGAP18, ARMC3, ASAP2, ATL1, ATP5E, BET1, C15orf26, C19orf33, C1orf198, C21orf7, C6orf25, C7orf41, CABP5, CALD1, CALM1, CALM2, CALM3, CD226, CD68, CD9, CDC14B, CERS2, CLCN3, CLEC12A, CLEC1B, CLIC4, CLU, CMIP, CMTM5, CREB3, CTSA, CTTN, DAAM1, DENND2C, EGF, EHD3, F13A1, FAM83D, FERMT3, FLNA, FN3K, GNAZ, GNB5, GNG11, GP1BA, GP1BB, GP6, GRAP2, GSTO1, GTPBP2, GUCY1A3, GUCY1B3, HIST1H3A, HIST1H3B, HIST1H3C, HIST1H3D, HIST1H3E, HIST1H3F, HIST1H3G, HIST1H3H, HIST1H3I, HIST1H3J, HOMER2, HSPD1, ILK, ITGA2B, ITGB1, ITGB3, JAM3, KIF2A, LAT, LGALSL, LIMS1, LIMS3, LIMS3L, LMNA, LOC100129617, LOC100129973, LOC100131860, LOC100288366, LOC100505813, LOC100506012, LOC100506387, LOC100507322, LOC100507472, LOC100652804, LOC100653288, LOC645513, LTBP1, LY6G6D, LY6G6E, LY6G6F, MAOB, MEIS1, MFAP3L, MMD, MMRN1, MPL, MYCT1, MYL9, NAA50, NAP1L1, NAT8B, NCKAP1, NEXN, NFIB, NRGN, PARVB, PCSK6, PDE3A, PDE5A, PDLIM1, PEX26, PF4, PF4V1, PKHD1L1, PKM, PLTP, POLH, PPBP, PRKAR2B, PRKG1, PRTFDC1, PRUNE, PTGS1, RAB27B, RBPMS2, RC3H2, RPS3A, RSU1, RUFY1, SAV1, SCFD2, SDC4, SDPR, SELP, SEPT5, SEPT5-GP1BB, SETDB1, SH3BGRL2, SIAE, SLC24A3, SLC40A1, SLC6A4, SNORD73A, SPARC, SPINT2, SSX2IP, STOM, SYTL4, THBS1, TLN1, TMEM45A, TNNC2, TPM1, TREML1, TSC22D1, TSPAN33, TSPAN9, TUBA1B, TUBA8, TUBB1, USP37, VCL, VIL1, VSIG2, WASF3, YIF1B, ZCCHC17*
11	*ABHD3, AIM2, HKR1, KREMEN1, LOC100128252, LOC100128398, LOC100288114, LOC100507342, LOC283788, NAG18, NOL7, PHKB, RANBP9, SND1, SNX27, TAF7, TRAPPC2, TRAPPC2P1, ZFP112, ZNF107, ZNF134, ZNF135, ZNF154, ZNF211, ZNF256, ZNF264, ZNF274, ZNF285, ZNF304, ZNF329, ZNF383, ZNF417, ZNF418, ZNF471, ZNF542, ZNF543, ZNF547, ZNF551, ZNF577, ZNF585B, ZNF586, ZNF587, ZNF587B, ZNF595, ZNF606, ZNF671, ZNF701, ZNF702P, ZNF776, ZNF805, ZNF806, ZNF876P, ZSCAN18, ZSCAN5A*
12	*CCNB1, CD5L, GJC2, GPR68, KLHL35, LCN15, TRIM3, TRIM63*
13	*ADSSL1, BARX1, CCDC142, ITPRIP, MRPL53, OR4C3, PPP2R1B, SDS, SLC25A19, TMEM9B*
14	*APLF, ARAP3, FLVCR1, GHRL, LOC100653018, LOC100653255, MAFG, NELL2, OPTN, SPTLC1, TTC25*

Cluster	Targeted Genes
15	*ADAM17, CDC42EP3, CHIC2, CPEB2, CYTH4, DCBLD2, DENND3, ELF2, F11R, FAM20A, FAM65B, FCAR, FCGR1A, FCGR1B, FCGR1C, FGD4, G0S2, GPR97, HIF1A, HIF1A-AS2, HIST2H2BF, IL13RA1, IVNS1ABP, JMJD1C, KIAA0226L, LOC100506271, LOC441009, PDE4B, PGM2, PLAUR, PRKAR1A, PTGS2, RGS2, RNF175, RPL21, SLC2A14, SLC2A3, SNORA27, SNORD102, ST3GAL6, TBC1D1, TLR2, TMEM71, TNFSF13B, TRPM6, TSTD1, UBE2J1, VNN3*
16	*ABCB10, CLK3, EDC3, GCLC, HEMGN, MAP2K3, MKNK2, PGM2L1, PIM1, RPIA, SCYL2, SLC6A9, SLC7A5, TCP11L2, TFDP1, TFDP3, TRAK2*
17	*C22orf13, C2orf88, CA1, CNOT7, DNAJB2, EPB49, FBXO7, FHOD3, GNAS, GNAS-AS1, GYPA, GYPB, GYPE, HK1, JAZF1, KIAA0430, LOC100291105, PCTP, PDZK1IP1, PPDPF, RBM38, RSRC1, SGIP1, SH3BGRL, SNRPD3, TBCEL, TFPI, TMEM56, TMEM56-RWDD3, TPGS2, VRK1, XK, ZDHHC2*
18	*ACP1, AHSP, AK1, ALDH6A1, ANKH, ARL4A, ARL6IP1, C14orf45, C17orf80, C18orf8, C22orf25, CARHSP1, COG1, CREG1, CTNNAL1, CYSTM1, DCK, DNAJB2, DNAJC6, EIF3B, EIF5, ENTPD5, ESPN, ESPNP, FAM104A, FGFR1OP2, FOXO4, GNA12, GRINA, GYPC, HBM, IGF2BP2, ITLN1, KAT2B, KIAA0430, KRT1, LGALS3, LOC100128164, LOC100130744, LOC100130930, LOC645195, MCOLN1, MKRN1, MOB1B, NEDD4L, NFE2, NPC1, ODC1, OPTN, OSBP2, PDZK1IP1, PIP4K2A, PIP5K1B, PITHD1, PLCL2, PLEK2, POC1B, POLR1D, PRDX5, PSMF1, PTMS, PTPLA, PURA, RAB2B, RANBP10, RIOK3, RNF10, SCYL2, SEC62, SLC14A1, SLC25A37, SLC25A39, SLC38A5, SMOX, SNCA, SPECC1, TAL1, TCP11L2, TESC, TMEM74B, TMOD1, TNFRSF25, TRAK2, TSNAXIP1, TSTD2, UBXN6, USP12, VN1R3, VWCE, YIPF6, ZFAND4*
19	*ACSL4, AGPAT9, AMPD2, ARSG, ATF6, BIN2, C20orf3, CASP2, CD300LF, CERS2, CKAP4, CPD, CYFIP2, DHRS9, DICER1, DOCK2, GAPVD1, GPR65, GUSBP11, HM13, HSDL2, IDI1, IDI2-AS1, JAK3, JMJD1C, MAPKAPK3, MPZL1, OGDH, P2RX1, PADI4, PAQR7, PILRA, PKN1, PRAM1, PSIMCT-1, RAB37, RGL4, RICTOR, SETDB1, SH2D3C, SLC11A1, SLC25A44, SRPK2, TCP11L2, TGM3, VAV1, WAS, WIPI1*
20	*ABCA13, BPI, CEACAM8, DEFA4, DEFA8P, LCN2, LTF, MMP8, OLFM4, OLR1, TCN1*
21	*BMP8A, BMP8B, BTF3, BTF3P9, HSPD1, IGF2BP3, KCNG2, OXCT2, TBC1D16*
22	*ACSL1, ARRB1, BCL2A1, BMX, C15orf37, CSF2RB, DYSF, EXTL3, HNRPDL, KIAA0319, LOC145474, LOC731424, MLPH, MTHFS, NLRC4, PIR, PROK2, PYGL, SIPA1L1, SRPK1, ST20, ST20-MTHFS, SYNE2, TDP2, WWC3*
23	*C11orf58, CDC34, DCAF6, NFIX, SLC25A37, SLC4A1, SLC7A5, STOM, TSPAN5, UBAP1, ZMAT2*
24	*ABHD2, AIF1, AKAP13, C2orf88, CACNB4, CD14, CD93, CHI3L1, CHSY1, CLEC2B, CNEP1R1, CYP4F2, CYP4F3, F11R, FCAR, GLIPR2, HEATR5A, HPSE, HRH2, IGF1R, IGF2R, ITGA5, KIAA0319L, LOC100132495, LOC100652973, LOC729603, MEGF9, MICAL2, MYL4, NOTCH2, NOTCH2NL, PCNX, PI3, PPP2R5B, PTPRJ, SERPING1, SGK1, SLC38A5, SPG21, STK38L, STXBP5, TNFRSF9, TSTD1, VNN2, WDFY3, WDFY3-AS2, YAP1, ZMYM4*
25	*ABCG2, AK1, ALDH5A1, ALDH6A1, AP2S1, APOO, ARL6IP1, ASH2L, BABAM1, BLVRB, C12orf29, C14orf45, C17orf80, C22orf13, C2orf88, C4A, C4B, C5orf4, CAPN5, CCBP2, CCDC90A, CHI3L1, CMAS, COG1, CSDA, CSDAP1, CTDSP2, CYB5A, CYP8B1, CYSTM1, DHFRL1, DNAJC6, DSERG1, EIF1B, EIF4EBP2, ENTPD5, ERCC2, F11R, FAM104A, FAM46C, FGFR1OP2, FLJ33065, FNBP1L, GPLD1, H1F0, HAGH, HEMGN, HK1, HRH2, IGF2R, ITLN1, ITSN1, KAT2B, KIAA0319L, KLC3, LGALS3, LOC100293534, LOC100505828, LOC645195, LOC729603, MAP2K3, MARCH8, MARK3, MBOAT2, MGST3, MICAL2, MYL4, NCOA4, NFIA, NOL7, NSUN3, NTAN1, OPA1, OPTN, OSBP2, PCGF5, PDCD10, PFDN5, PIK3R2, PIP5K1B, PPP2R5B, PTK2B, PTMS, PURA, RAB2B, RAD23A, RANBP9, RASGRP3, RIOK3, ROGDI, RPIA, SCYL2, SFRP2, SH3BGRL3, SHARPIN, SHISA4, SIAH2, SLC38A5, SLC7A5, SNCA, SNRPD3, SPECC1, STOML2, STXBP1, TFDP1, TFDP2, TFDP3, TFRC, TMEM86B, TMEM9B, TRAK2, TSTA3, TSTD1, UBXN11, USP7, VWCE, WDFY3, WDFY3-AS2, ZMYM4*
26	*C15orf54, ENKUR, HSPD1, MAP1B, MYCT1, PPBP, RGS18*
27	*B4GALT5, FAM126B, FRY, HNRNPA1, MSL3, SLC9A8, TXNRD1*
28	*C15orf54, EHD3, ENKUR, HSPD1, LIMS1, LIMS3, LIMS3L, MYCT1, RAB27B, RHOBTB1*

Cluster	Targeted Genes
29	*ADAMTSL4, AHR, ARHGAP26, ARHGEF40, C1orf138, CCDC19, CCPG1, CD97, CREB1, CRIP1, DMXL2, DYX1C1-CCPG1, EMR2, ERGIC2, FAR2, JMJD1C, LOC100506290, LOC100506606, LOC100653153, MCL1, METTL21A, MS4A6A, MYADM, PIGB, RFX2, SERPINI2, TAGAP, TIPARP, TIPARP-AS1, TSPAN2, VIM, WDR49, ZNF219*
30	*ABCA13, BPI, CEACAM3, CEACAM5, CEACAM6, CEACAM8, DEFA4, DEFA8P, ERG, MMP8, OLFM4, OLR1, PCOLCE2*
31	*BMP10, CCDC155, FGF11, LINC00174, SCN1B, VPS37B*
32	*ALMS1, ALMS1P, CCL5, ELSPBP1, FAM71E1, MGC40069, MRPL4, OR4D1, OR4D11, PRELP, TRAV8-3*
33	*AQP10, CLU, CNST, CXCL5, ITGA2B, ITGB3*
34	*ABLIM3, GUCY1B3, ITGB3, LTBP1, MMRN1, PROS1*
35	*HIST1H1C, HIST1H2AC, HIST1H2AD, HIST1H2BD, HIST1H3A, HIST1H3B, HIST1H3C, HIST1H3D, HIST1H3E, HIST1H3F, HIST1H3G, HIST1H3H, HIST1H3I, HIST1H3J, HIST1H4A, HIST1H4B, HIST1H4C, HIST1H4D, HIST1H4E, HIST1H4F, HIST1H4H, HIST1H4I, HIST1H4J, HIST1H4K, HIST1H4L, HIST2H2AA3, HIST2H2AA4, HIST2H2BE, HIST2H2BF, HIST2H3A, HIST2H3C, HIST2H3D, HIST2H4A, HIST2H4B, HIST4H4*
36	*ASPH, DOCK4, HEY1, KIAA0319, RB1, RCBTB2, SHKBP1, TDP2*
37	*ALDH6A1, C14orf45, DCK, ENTPD5, HPS1, MOB1B, PGM2L1, PSME4, PSMF1, TMEM74B, ZFAND4*
38	*ABLIM3, APP, ASPH, BMP6, C15orf54, C1orf198, CREB3, CTDSPL, CTTN, DCLRE1A, DOCK4, HEY1, HSPD1, ITGA2B, ITGB3, KIAA0319, LOC100505813, LOC283788, LTBP1, MPZL3, MYCT1, MYL9, NAT8B, PI3, PTGS1, RAB27B, RB1, RCBTB2, RHOBTB1, SHKBP1, SPARC, TDP2, TLN1, ZNF542, ZSCAN5A*
39	*ARHGEF40, CRIP1, FCGR1A, FCGR1B, FCGR1C, GBP1, GBP2, GBP3, GBP4, GBP5, GBP7, GLS, HIST2H2BF, LOC100131190, LPCAT2, MX2, MYADM, OBFC1, PSTPIP2, RICTOR, SOCS6, STAT1, TAGAP, USP4, UTP18, ZNF219*
40	*APP, C6orf25, CLU, CTTN, ITGA2B, ITGB3, LOC100505813, LTBP1, MYLK, PROS1, SPARC, THBS1, VCL*
41	*ATP5SL, C17orf62, CLDN15, FBXL22, FRAT2, GK, GK3P, GRM3, HERC1, HPS6, LCE3C, LOC100130855, NXF5, PNLDC1, REEP2, SMTNL2, SPSB4, STAP1, ZNF439, ZNF440*
42	*ASPM, C7orf41, CDC7, CTSB, EIF1B, EMC3, FBXO9, FLJ33065, GYPA, GYPB, OPA1, PPDPF, SEPT14, SH3BGRL, WDR67, ZMAT2*
43	*CD58, CXorf65, FLVCR1, GHRL, HCAR1, HCAR2, HCAR3, IFIH1, IL1B, IL2RG, KLHL5, MT1G, NAP1L4, NBN, NFKB2, PI3, PLXNC1, RELB, REPS2, RNF19B, SBF2, TTLL4*
44	*BTN2A1, BTN2A2, BTN3A1, BTN3A2, BTN3A3, HCP5, HLA-E, HLA-F, LOC100507463, LOC100653072, PSMB8, PSMB9, SPG11, TAP1*
45	*AIPL1, CCDC37, CD6, CLK2, CLK2P, CYP2C19, GSK3A, OVOL2, STAMBPL1, ZNF746*
46	*EPS8L1, ERICH1-AS1, FERD3L, IDUA, IRAK1, KCNK5, LOC100286925, LOC401442, OR7E154P, OTOP1, PPM1F, RBMXL3, SLC26A1, ZNF414*
47	*ERICH1-AS1, FBXO17, FERD3L, IRAK1, KCNK5, KIAA1274, LOC401442, RBMXL3, SARS2, ULK3, ZNF414*
48	*BCL6, CCNJL, IL1R1, IL1RAP, IL8, P2RY13, TBL1X, TBL1XR1*
49	*ACP1, ADIPOR1, AHSP, ARG2, ATG9A, C5orf4, C9orf153, CA1, DCAF12, DNAJB4, EMC3, EPB41, FAM46C, GCLC, GMPR, ISCA1, ISCU, KIAA1586, LOC100291105, LUZP6, MAP1LC3B, MAP1LC3B2, MBOAT2, MKRN1, MTPN, NCOA4, ODC1, OPA1, PCGF5, RAB2B, RBM38, RGS10, RNF10, SCYL2, SELK, SFRP2, SIAH2, SLC14A1, SLC25A37, SRRD, SWT1, TBCEL, TCP11L2, TFIP11, TGM2, TMC5, TMEM200B, TRAK2, TSPAN7, VN1R3, VTI1B, YIPF6, ZFAND2B, ZNF23, ZNF451*
50	*AKR1C3, B4GALT6, BNC2, CD160, CD247, CD38, CD8A, GLS, GZMB, GZMH, IGFBP7, IL2RB, KLRF1, LINC00299, LOC100130872, LOC646778, NCAM1, PRF1, RNF165, SH2D1B, SLC4A7, SPON2, STAT1, TBC1D19, TKTL1, TXK*

Cluster	Targeted Genes
51	*ACIN1, ACP1, AHSP, AKT2, ALDH6A1, AMIGO3, ANK1, ANP32B, AP2A1, APP, AQP1, ARF1, ARG2, ARHGEF12, ATP11B, ATP5E, BAG6, BLVRB, BMP6, BNIP3L, BPGM, BST2, C12orf51, C14orf45, C15orf54, C22orf13, C22orf25, C22orf32, C2orf88, C6orf25, C7orf41, CA1, CA2, CARM1, CCNI, CLCN3, CLEC12A, CLEC1B, CLU, CNPPD1, CNST, COPS2, CORO1C, CREB3, CSDE1, CTDSPL, DCAF12, DCLRE1A, DCUN1D1, DDB1, DPM2, EEF2, EIF1B, EIF2C2, EMC3, ENTPD5, EPB49, ESPN, ESPNP, F13A1, FAM134A, FECH, FIS1, FKBP8, FLJ33065, FLNA, FOXO4, FURIN, FUZ, GABARAPL2, GADD45A, GMPPB, GNAZ, GNG12, GYPA, GYPB, GYPE, HBD, HEMGN, HIST1H2AB, HIST1H2AE, HSPD1, ITLN1, KIAA0430, LGALS3, LOC100130930, LOC100505813, LOC100506023, LOC100507472, LOC100631377, LOC653562, LRP10, MAK, MAN2A2, MAOA, MAP1LC3B, MAP1LC3B2, MARCH8, MCOLN1, MICAL2, MICALCL, MIR3620, MYCT1, MYL9, NCOA4, NDUFA3, NDUFA6, NEXN, NRGN, OPA1, OR2AJ1, OR2W3, PCMTD1, PCSK6, PDCD10, PGRMC1, PIM1, PINK1, PITHD1, PKHD1L1, PLEKHG3, PLVAP, PNPLA2, POLL, PPM1A, PPP6R1, PRDX6, PTGS1, RAB11A, RAB27B, RBX1, RGS10, RGS18, RNF10, RNF123, RPL7AP60, RSU1, RXRA, SCYL2, SDPR, SELK, SELP, SEPT11, SH3BGRL2, SLC6A10P, SLC6A8, SNORD37, SNRPD3, SPARC, SPTB, ST3GAL1, STK11, STOM, TAX1BP1, TIMM23, TLN1, TMEM14B, TNFRSF25, TNFSF4, TNS1, TRAK2, TREML1, TRIM23, TRIM58, TSPAN5, TSPAN9, TUBB1, UBXN6, UQCR11, VCL, VN1R3, VTI1B, VWCE, WASF2, WDTC1, WNK1, XPNPEP3, YIPF2, YY1AP1, ZER1, ZMAT2*
52	*APP, C6orf25, C7orf41, CTDSPL, EPB49, FLNA, LOC100505813, MYL9, NRGN, PTGS1, RGS18, SPARC*
53	*ASCC2, BSG, FIS1, HEXIM1, MYL4, PSMF1, RAB2B, ROGDI, TMEM74B, UBAP1, UBB*
54	*ADIPOR1, ALDH6A1, ARF1, BAG1, BPGM, BSG, C14orf45, C5orf4, CA1, CAPRIN2, CARM1, CETN2, COPS2, CTSB, DENND4A, DNAJB14, ENTPD5, FAM214B, GYPA, GYPB, GYPE, LOC100131015, LOC646358, MBNL3, MIR3620, RHBDD1, RIOK3, RNF10, SLC14A1, SLC1A5, SLC25A28, SLC4A1, SLC6A9, SLC7A5, SOX6, SPECC1, STIL, TFRC, TIAL1, TMEM56, TMEM56-RWDD3, TRIP12, UBA52, UBE2O, USP7, VRK1, WBP2, WDR67, YIPF2, ZMAT2*
55	*EHD2, GGT5, GNA11, IZUMO4, KRT86, LILRA4, SYNM, TFDP1, TFDP3*
56	*DGKG, MANSC1, MTOR, MTOR-AS1, NCKAP1L, PI4KB, PRR13, ZNF687*
57	*ABCA13, BPI, CEACAM8, CRISP3, DEFA4, DEFA8P, LCN2, MMP8, OLFM4*
58	*CDH3, DVL3, FAM167A, FAM43A, GPBAR1, HERPUD1, IRX5, LOC100288144, OR51G2, PTGES2, REEP6, RTEL1, RTEL1-TNFRSF6B, SCN2B, SERPINB13, SGCE, TAF1L, THRSP, TMEM179, TNFRSF6B, TUSC3*
59	*ABCC13, AK1, C5orf4, CDC34, DHFRL1, E2F2, EPB41, GDE1, GUK1, GYPC, HAGH, HBM, HDGF, JAZF1, KIAA0430, LOC100291105, MAF1, MAP2K3, NCOA4, NSUN3, PLEKHG3, RBM38, ROGDI, RPIA, SCYL2, SLC14A1, SPTB, ST6GALNAC4, TBCEL, TCP11L2, TFDP1, TFDP3, TMEM200B, TRAK2, UBAP1, UBB, UBE2O*

*Genes listed for each cluster were found to be associated with at least one eQTL in the cluster at FDR≤0.05. All genes targeted by each Affymetrix transcript cluster are listed.

Table S8. Clusters related to transcription factors

Cluster No.	Transcription Factor Motif	Transcription Factor Gene	FDR
1	TTCYNRGAA	*STAT5B*	4.3E-04
1	NAWTTCYNGGAAWTN	*STAT5B*	5.9E-03
10	RYTTCCTG	*ETS2*	1.4E-08
10	GGGCGGR	*SP1*	4.6E-07
10	CAGGTG	*TCF3*	5.7E-06
10	CATTGTYY	*SOX9*	8.2E-06
10	GGGAGGRR	*MAZ*	3.8E-05
18	GGGCGGR	*SP1*	8.2E-04
18	CAGGTG	*TCF3*	1.2E-03
18	GGGAGGRR	*MAZ*	1.6E-03
18	CTTTGA	*LEF1*	5.2E-03
19	RNWMBAGGAART	*ELF1*	2.5E-03
19	MGGAAGTG	*GABPA*	4.6E-03
19	RGAGGAARY	*SPI1*	4.6E-03
19	RYTTCCTG	*ETS2*	4.6E-03
24	CTTTGT	*LEF1*	3.5E-03
25	GGGCGGR	*SP1*	2.3E-03
25	CTTTGA	*LEF1*	2.5E-03
25	TGCCAAR	*NF1*	2.6E-03
25	TTGTTT	*MLLT7*	2.6E-03
25	GGGTGGRR	*PAX4*	3.4E-03
25	TGAMCTTTGNCCN	*HNF4A*	6.3E-03
25	TGAMCTTTGMMCYT	*HNF4A*	6.4E-03
29	TGGAAA	*NFAT/NFATC*	8.5E-03
35	TATAAA	*TAF/TATA*	8.8E-04
35	TATGCAAATN	*POU2F1*	6.8E-03
38	KRCAGGAARTRNKT	*ETS2*	8.6E-04
38	CGTACGTGCNGB	*HIF1A*	8.2E-03
38	WNTAATCCCAR	*PITX2*	9.0E-03
40	DCCWTATATGGNCWN	*SRF*	1.1E-04
40	TGANTCA	*JUN*	1.7E-03
40	CCAWATAWGGMNMNG	*SRF*	4.2E-03
40	SCCAWATAWGGMNMNNNN	*SRF*	4.2E-03
40	GNCCAWATAWGGMN	*SRF*	5.2E-03
40	WNTAATCCCAR	*PITX2*	5.7E-03
40	ATGCCCATATATGGWNNT	*SRF*	8.7E-03
43	NNNGATRNNN	*GATA2*	4.7E-03
44	GAAAAGYGAAASY	*IRF2*	8.4E-04
44	RAARTGAAACTG	*IRF8*	4.0E-03
44	SAAAAGYGAAACC	*IRF1*	4.0E-03
49	GTGACGY	*E4F1*	4.6E-05
51	GGGCGGR	*SP1*	4.6E-12
51	RCGCANGCGY	*NRF1*	8.1E-07
51	KRCAGGAARTRNKT	*ETS2*	2.2E-05
51	GGGAGGRR	*MAZ*	2.3E-05
51	GTGACGY	*E4F1*	1.7E-04

*Specific GSEA gene sets (defined by promoter motifs and corresponding transcriptions factors) were compared to the gene sets targeted by each cluster, and the corresponding enrichment FDRs were calculated.

Table S9. Clusters targeting transcripts that as a group, are significantly enriched with targets of micro RNAs.

Cluster No.	GSEA gene set related to Micro RNA targets**	Number of genes targeted by cluster that are also associated with measured levels of at least one of these miRNA***	Number of genes targeted by the cluster
8	MIR-192, MIR-215*	13	26
17	MIR-136*	N/S	26
18	MIR-515-3P*, MIR-524*, MIR-506*	N/S	73
19	MIR-196A*, MIR-196B, MIR-148A, MIR-152, MIR-148B	7	43
25	MIR-323, MIR-124A*	0	93
29	MIR-142-5P, MIR-506*	6	20
43	MIR-31	0	18
49	MIR-515-3P*, MIR-124A*		44
50	MIR-30, MIR-452*	2	23
51	MIR-15A, MIR-16, MIR-15B, MIR-195, MIR-424, MIR-497*	119	141

* These miRNAs are not available
** To obtain microRNA targets per cluster, we performed GSEA analysis (on microRNA target category) on transcripts targeted by each cluster. We filtered the GSEA results at FDR<0.05. GSEA may output multiple microRNAs for one cluster. We list all the microRNA results for each cluster in this column.
*** After the GSEA analysis, we confirmed our findings to see if the microRNA-transcript pair are indeed observed in our microRNA-mRNA coexpression database above. We reported the number of confirmed transcripts per cluster in this column.

Table S10. GWAS-associated cis- and trans-eQTLs, that overlap with the NHGRI GWAS catalog (downloaded on June 5, 2016, filtered by association p<5E-8).

Available as a separate spreadsheet in Additional file 2.xlsx. Sheet 1: 2359 rows; Overlaps with lead eQTLs or with >80% R2 of lead eQTL. Sheet 2: 13,354 rows; Overlaps with all significant eQTLs.

Table S11. 58 Coronary Artery Disease GWAS Loci

Locus Name	New Lead variant	Chr	Position	Cytoband	Reported p-value	Position Relative to Nearest Gene(s)	Published eGenes
7q22	rs10953541	7	107244545	7q22.3	1.0E-05	Intronic to BCAP29	
9p21	rs2891168	9	22098619	9p21.3	2.3E-98	Intronic to CDKN2B-AS1, upstream of CDKN2B	*CDKN2B*
ABCG5-ABCG8	chr2:44074126:D or alias rs6544713	2	44074126	2p21	2.6E-08	Intronic to ABCG8.	
ABO	rs2519093	9	136141870	9q34.2	1.2E-11	intronic to ABO	*ABO*
ADAMTS7	rs4468572	15	79124475	15q25.1	4.4E-16	upstream (20kb) of ADAMTS7	
ADTRP-C6orf105	rs6903956	6	11774583	6p24.1	9.6E-01	Intronic to ADTRP	
AK097927	rs16986953	2	19942473	2p24.1	1.5E-08	downstream (150kb) of WDR35	
ANKS1A	rs17609940	6	35034800	6p21.31		intronic to ANKS1A	
APOB	rs192011340	2	21378433	2p24.1	2.9E-08	downstream of TDRD15	
APOE-APOC1	rs4420638	19	45422946	19q13.32	7.1E-11	downstream of APOC1	*TOMM40*
ATP2B1	rs2681472	12	90008959	12q21.33	6.2E-11	intronic to ATP2B1	
BCAS3	rs7212798	17	59013488	17q23.2	1.9E-08	intronic to BCAS3	
COL4A1/A2	rs11838776	13	111040681	13q34	1.8E-10	intronic to COL4A2, upstream of COL4A1	
CXCL12	rs1870634	10	44480811	10q11.21	5.6E-15	downstream of LINC00841, LINC00840	
CYP17A1-CNNM2-NT5C2	rs11191416	10	104604916	10q24.32	4.7E-09	upstream of CYP17A1, BORCS7	
EDNRA	rs4593108	4	148281001	4q31.22	8.8E-10	upstream (200kb) of EDNRA	
FLT1	rs9319428	13	28973621	13q12.3	7.1E-05	intronic to FLT1	
FURIN-FES	rs17514846	15	91416300	15q26.1	3.1E-07	intronic to FURIN, upstream of FES	*FES, FURIN*
GUCY1A3	rs72689147	4	156639888	4q32.1	6.1E-09	intronic to GUCY1A3	

Locus Name	New Lead variant	Chr	Position	Cytoband	Reported p-value	Position Relative to Nearest Gene(s)	Published eGenes
HDAC9	rs2107595	7	19049388	7p21.1	8.1E-11	downstream of HDAC9	
HHIPL1	rs10139550	14	100145710	14q32.2	1.4E-08	downstream of HHIPL1, upstream of CYP46A1	
IL6R	rs6689306	1	154395946	1q21.3	2.6E-09	first intron of IL6R	*IL6R**
KCNE2	rs28451064	21	35593827	21q22.11	1.3E-15	downstream (100kb) of SLC5A3, downstream of LINC00310, upstream (100kb) of KCNE2	*MRPS6*
KCNK5	rs56336142	6	39134099	6p21.2	1.9E-08	downstream of KCNK5, upstream (50kb) of SAYSD1	
KIAA1462	rs2487928	10	30323892	10p11.23	4.4E-11	intronic to KIAA1462	
KSR2	rs11830157	12	118265441	12q24.23	3.9E-04	intronic to KSR2	*RFC5*
LDLR	rs56289821 (old lead variant rs1122608 used instead)	19	11187997	19p13.2	4.4E-15	downstream of SMARCA4, upstream of LDLR, downstream (200 kb) of DNM2, ILF3	*SMARCA4**
LIPA	rs1412444	10	91002927	10q23.31	5.2E-12	intronic to LIPA	
LPL	rs264	8	19813180	8p21.3	1.1E-5	intronic to LPL	*LPL**
MFGE8-ABHD2	rs8042271	15	89574218	15q26.1	3.7E-08	upstream of MFGE8, ABHD2.	
MIA3	rs67180937	1	222823743	1q41	1.0E-12	intronic to MIA3	
MRAS	rs139016349	3	138099161	3q22.3	2.9E-09	intronic to MRAS	
NOS3	rs3918226	7	150690176	7q36.1	1.7E-09	intronic to or upstream of NOS3	
PCSK9	rs11206510	1	55496039	1p32.3	2.3E-08	upstream of PCSK9	
PDGFD	rs2128739	11	103673277	11q22.3	7.1E-11	downstream (100kb) of PDGFD	
PHACTR1	rs9349379	6	12903957	6p24.1	1.8E-42	intronic to PHACTR1	
PLG	rs4252185	6	161123451	6q26	1.6E-32	intronic (first intron) to PLG	*PLG*
PMAIP1-MC4R	rs663129	18	57838401	18q21.32	3.2E-08	nearest genes (200kb) are MC4R and PMAIP1	

Locus Name	New Lead variant	Chr	Position	Cytoband	Reported p-value	Position Relative to Nearest Gene(s)	Published eGenes
POM121L9P-ADORA2A	rs180803	22	24658858	22q11.23	1.6E-10	intronic to POM121L9P	
PPAP2B	rs9970807	1	56965664	1p32.2	5.0E-14	Intronic (last intron) to PLPP3	
RAI1-PEMT-RASD1	rs12936587	17	17543722	17p11.2	8.2E-04	120 kb downstream of SREBF1, closest gene is RAI1, then upstream of PEMT	*RASD1, SMCR3, PEMT*
REST-NOA1	rs17087335	4	57838583	4q12	4.6E-08	Intronic to NOA1, upstream to POLR2B, 40kb downstream of REST	*LOC285453, IGFBP7, REST**
SH2B3	rs3184504	12	111884608	12q24.12	1.0E-09	exonic to SH2B3 (2nd exon, in translated region). Downstream of ATXN2. 200kb upstream of ALDH2, 500 kb downstream of TMEM116	
SLC22A3-LPAL2-LPA	rs55730499	6	161005610	6q25.3	5.4E-39	intronic to LPA, 120kb upstream of PLG, 100 kb downstream of SLC22A3	
SLC22A4-SLC22A5	rs273909	5	131667353	5q31.1	1.2E-04	intronic to SLC22A4, 40 kb upstream of SLC22A5, 120 kb upstream of P4HA2, 120 kb downstream of IRF1	*SLC22A4-SLC22A5*
SMAD3	rs56062135	15	67455630	15q22.33	4.5E-09	intronic (first intron) to or upstream of SMAD3. PIAS1 is 700 kb distant	
SMG6	rs216172	17	2126504	17p13.3	5.1E-07	intronic to SMG6	*SMG6*

Locus Name	New Lead variant	Chr	Position	Cytoband	Reported p-value	Position Relative to Nearest Gene(s)	Published eGenes
SORT1	rs7528419	1	109817192	1p13.3	2.0E-23	Exonic (3' UTR) to CELSR2, downstrem off PSRC1, 25 kb downstream of SORT1. 300kb upstream of GSTM4, GSTM3	*CELSR2, PSRC1*, SORT1*
SWAP70	rs10840293	11	9751196	11p15.4	1.3E-08	intronic to SWAP70	*SWAP70*, ADM, AC026250.16*
TCF21	rs12202017	6	134173151	6q23.2	2.0E-11	Intronic to TARID, 3' end of LINC01312	*TCF21*
TRIB1	rs2954029	8	126490972	8q24.13	2.6E-06	downstream 40kb of TRIB1	
UBE2Z	rs46522	17	46988597	17q21.32	1.8E-05	Intronic to UBE2Z, downstream of SNF8 and ATP5G1	*UBE2Z**
VAMP5-VAMP8-GGCX	rs7568458	2	85788175	2p11.2	3.6E-10	Intronic to GGCX, upstream of VAMP8, VAMP5	*VAMP8*, GGCX*, VAMP5*
WDR12	chr2:203828796 :I or perfect alias rs6725887	2	203828796	2q33.1	2.2E-18	Intronic to CARF(aka ALS2CR8)	
ZC3HC1	rs11556924	7	129663496	7q32.2	5.3E-11	exonic to ZC3HC1, 40 kb upstream of KLHDC10	
ZEB2-ACO74093.1	rs17678683	2	145286559	2q22.3	3.0E-09	intronic to LINC01412	
ZNF259-APOA5-APOA1	rs964184	11	116648917	11q23.3	5.6E-05	exonic to ZPR1 (3' UTR),200kb distant from SIDT2, TAGLN, PCSK7	
ZNF507-LOC400684	rs12976411	19	32882020	19q13.11	9.1E-03	intronic to LOC400684, downstream of ZNF507	

-Locus Name, New Lead variant, Reported p-value as described in reference[12]
-Published eGenes[11–13]

Table S12. Targets of eQTLs in strong LD (R^2>=90%) with Published Risk Lead Variant by Locus.

Locus Name	Risk Lead Variant	Best eQTL	LD R2	Target Gene Symbol	Transcript Cluster ID	Trans?	Lead eQTL[C]	P-value
ABO	rs2519093	rs600038	97%	*ABO* [a]	3228582		LV	2.2E-12
ADAMTS7	rs4468572	rs7166764	100%	*CTSH*	3634811			2.5E-39
ANKS1A	rs17609940	rs12201824	92%	*SNRPC*	2904248			2.9E-07
ATP2B1	rs2681472	rs11105337	100%	*POC1B-GALNT4*	3464967			2.5E-06
"	"	rs111478946	100%	*ATP2B1* [d]	3464983			3.8E-05
CYP17A1-CNNM2-NT5C2	rs11191416	rs11191582	92%	*NT5C2*	3304624			8.7E-54
"	"	rs4409766	92%	*AS3MT*	3261923		LV2	8.1E-07
"	"	rs17115100	100%	*WBP1L*	3261886			8.7E-05
"	"	rs7475853	92%	*RNF103-CHMP3*	2562821	TRANS	LV	4.7E-08
IL6R	rs6689306	rs11265612	94%	*IL6R* [a]	2360257		LV	4.8E-24
KCNE2	rs28451064	**rs28451064**	100%	*SLC5A3*	3919033			5.2E-06
LDLR	rs1122608	rs112186070	100%	*SMARCA4* [a]	3820921			4.5E-20
LIPA	rs1412444	rs1412445	100%	*LIPA*	3299585		LV	<1E-450
LPL	rs264	**rs264**	100%	*LPL* [a]	3088486			6.0E-41
MIA3	rs67180937	rs17163358	100%	*FAM177B*	2381903			4.7E-27
MRAS	rs139016349	rs2306374	100%	*MRAS* [b]	2644565		LV	1.0E-11
"	"	rs6807945	97%	*CEP70*	2697490			3.4E-06
"	"	rs6807945	97%	*DBR1*	2697331			7.0E-05
REST-NOA1	rs17087335	rs6554401	100%	*REST* [a]	2728408			4.4E-05
"	"	rs56281640	97%	*GDAP1*	3103607	TRANS	LV	1.6E-21

Locus Name	Risk Lead Variant	Best eQTL	LD R^2	Target Gene Symbol	Transcript Cluster ID	Trans?	Lead eQTL[c]	P-value
"	"	rs6852182	97%	*CACNA1E*	2370433	TRANS	LV	1.5E-08
SH2B3	rs3184504	**rs3184504**	100%	*OAS2*	3432514		LV	7.8E-11
"	"	rs7137828	100%	*SH2B3*	3431892			2.7E-06
"	"	**rs3184504**	100%	*GBP5*	2422035	TRANS	LV	7.9E-25
"	"	**rs3184504**	100%	*STAT1*	2592268	TRANS	LV	4.0E-17
"	"	**rs3184504**	100%	*GBP7*	2421925	TRANS	LV	3.0E-13
"	"	**rs3184504**	100%	*GBP1*	2421883	TRANS	LV	2.4E-12
"	"	rs7310615	100%	*GBP4*	2421995	TRANS	LV	4.6E-12
"	"	**rs3184504**	100%	*PSTPIP2*	3806211	TRANS	LV	3.2E-11
"	"	rs597808	98%	*ATP2B1* [d]	3464983	TRANS	LV	5.1E-11
"	"	**rs3184504**	100%	*MYADM* [d]	3841076	TRANS	LV	1.3E-10
"	"	**rs3184504**	100%	*FCGR1B*	2357845	TRANS	LV	2.6E-10
"	"	**rs3184504**	100%	*TAGAP* [d]	2982076	TRANS		4.0E-10
"	"	rs7310615	100%	*USP4*	2674179	TRANS	LV	2.2E-08
"	"	rs597808	98%	*CRIP1* [d]	3554851	TRANS	LV	3.3E-08
"	"	rs7310615	100%	*RICTOR*	2854241	TRANS		3.8E-08
"	"	rs7310615	100%	*OBFC1*	3305017	TRANS	LV	3.8E-08
"	"	rs597808	98%	*MX2*	3922037	TRANS	LV	4.2E-08
"	"	rs7310615	100%	*ARHGEF40* [d]	3527864	TRANS	LV	6.7E-08
SMG6	rs216172	rs216185	91%	*SRR*	3706219			1.5E-24
SORT1	rs7528419	rs646776	100%	*PSRC1* [a]	2426951		LV	1.1E-12
SWAP70	rs10840293	**rs10840293**	100%	*SWAP70* [a]	3319997			1.5E-25
UBE2Z	rs46522	rs9747646	100%	*UBE2Z* [a b]	3725481		LV	3.2E-116
"	"	rs12601672	100%	*SNF8*	3761632		LV	2.4E-13
"	"	rs524808	96%	*CALCOCO2*	3725392			1.0E-05

Locus Name	Risk Lead Variant	Best eQTL	LD R^2	Target Gene Symbol	Transcript Cluster ID	Trans?	Lead eQTL[c]	P-value
UBE2Z	rs46522	rs1973408	94%	*ATP5G1*	3725456		LV	9.0E-05
VAMP5-VAMP8-GGCX	rs7568458	rs12714145	90%	*VAMP8* [a b]	2491661		LV	9.0E-304
"	"	rs12714145	90%	*GGCX* [a]	2562343		LV	2.54E-51
"	"	**rs7568458**	100%	*RNF181*	2491686			5.7E-08
"	"	**rs7568458**	100%	*DPEP3*	3696115	TRANS		2.9E-12
"	"	rs12714145	90%	*CRISPLD2*	3671935	TRANS		2.8E-08
"	"	rs12714145	90%	*SLC26A8*	2951730	TRANS		1.1E-07
"	"	rs10172544	90%	*PKN2*	2345617	TRANS		1.1E-07
"	"	rs10198569	90%	*CASP5* [d]	3389330	TRANS	LV	1.3E-07
WDR12	rs6725887	rs149268645	100%	*FAM117B*	2523354			1.7E-81
"	"	rs149846585	100%	*CARF(ALS2CR8)*	2523419		LV	9.8E-41
"	"	rs80087860	100%	*NBEAL1*	2523478			1.8E-08
ZNF507-LOC400684	rs12976411	rs16966931	100%	*ZNF507*	3828887			1.5E-09

- Locus Name, Risk Lead Variant from reference[11]
- LD R^2 – measured in FHS population between Risk Lead Variant and Best eQTL
[a] eGene is mentioned in recent papers[11–13]
[b] Polymorphism-in-Probe may affect cis-eQTL results (see Methods)
[c] LV means variance explained (R^2) for Best eQTL as fraction of R^2 for lead eQTL for gene is >=90%.
[d] Genes found in Huan, et al[14] as significantly associated with blood pressure.
